# Lactate dynamics in Parkinson’s disease: striatal circuit vulnerability, biomarker potential, and therapeutic windows

**DOI:** 10.1186/s10020-026-01551-z

**Published:** 2026-07-06

**Authors:** Jianda Kong, Zizheng Yang, Yinhao Wang, Lingyun Liu, Yang Liu, Wei Chen

**Affiliations:** 1 School of Physical Education, Hebei Normal University, Shijazhuang, China; 2 Key Laboratory of Measurement and Evaluation in Exercise Bioinformation of Hebei Province, Shijazhuang, China

**Keywords:** Parkinson’s disease, Lactate, Striatum, D2 medium spiny neurons, Biomarker, Monocarboxylate transporters, HCAR1, Precision rehabilitation

## Abstract

Lactate is a mobile carbon substrate, extracellular signal, and candidate mediator of metabolism-to-gene coupling. These properties make lactate relevant to Parkinson’s disease (PD), but current evidence does not support a simple protective or toxic interpretation. In this Review, we synthesize evidence that lactate dynamics may influence PD-relevant striatal biology through substrate provision, redox-sensitive NMDAR–ERK/MAPK–CREB signaling, HCAR1-mediated receptor signaling, and the emerging lactylation-linked modification of inflammatory or transcriptional states. We explicitly distinguish established non-PD mechanisms from direct PD-model evidence and from hypotheses that remain untested in D2 medium spiny neurons (D2-MSNs). We propose that the apparent lactate paradox in PD is best understood as a kinetic and compartmental problem: transient, well-cleared lactate pulses, such as those produced during exercise, may support metabolic flexibility, neurovascular adaptation, and selected forms of plasticity, whereas sustained lactate accumulation in mitochondrially constrained or inflammatory environments may promote acid–ion stress, oxidative injury, neuroimmune activation, and impaired dopaminergic signaling. The D2-MSN axis is therefore presented not as a proven site of lactate-mediated rescue, but as a high-priority experimental test bed for linking lactate dynamics to hypokinetic network states. Translationally, lactate is unlikely to serve as a stand-alone diagnostic biomarker. Its more realistic near-term uses are as a pharmacodynamic readout, a challenge–response variable for exercise dosing, and a target-engagement marker for interventions that modify lactate transport, signaling, or inflammatory metabolic state. This framework defines lactate kinetics, compartment, and disease context as the critical variables for biomarker-guided rehabilitation and future PD trials.

## Background

Parkinson’s disease (PD) poses a growing socioeconomic burden, driven mainly by population aging and rising prevalence across regions (Global, regional, and national burden of Parkinson’s disease, 1990–2016: a systematic analysis for the Global Burden of Disease Study 2016 2018). However, the therapeutic landscape remains dominated by approaches that compensate for dopaminergic loss rather than addressing the broader pathophysiological milieu in which degeneration unfolds (Global, regional, and national burden of Parkinson’s disease, 1990–2016: a systematic analysis for the Global Burden of Disease Study 2016 2018). This dissonance is especially evident in the motor system: levodopa and associated dopaminergic therapies can substantially improve symptoms, but often lose efficacy as the disease progresses and can introduce additional liabilities through pathological plasticity and dyskinesia (Global, regional, and national burden of Parkinson’s disease, 1990–2016: a systematic analysis for the Global Burden of Disease Study 2016 2018). Against this backdrop, PD is increasingly being reconceptualized as a disorder of state control within cortico–basal ganglia (BG) loops, in which neuromodulatory signals, synaptic learning rules, and cellular energetic bottlenecks jointly shape motor output rather than the disease being viewed as purely dopaminergic.

The striatum is the computational hub of the BG motor circuit, combining dense glutamatergic input from cortex and thalamus and routing these inputs into direct and indirect pathways that gate the probability, vigor and selection of actions. The classical dichotomy between “movement-facilitating” direct pathways and “movement-suppressing” indirect pathways has been nuanced by cell-type-resolved recordings demonstrating that both pathways are phasically engaged around action initiation, indicating coordinated facilitation and suppression rather than a simple dichotomous switch (Cui et al. 2013). In addition, causal circuit manipulations illustrate that rebalancing these pathways can bidirectionally modulate parkinsonian motor behaviors, corroborating pathway-level imbalance as a pathophysiological substrate of hypokinesia (Kravitz et al. 2010). These circuit-level insights sharpen a central question: which endogenous variables can stabilize striatal computation when dopaminergic buffering fails?

Metabolic insufficiency is an increasingly plausible part of the answer. Mitochondrial dysfunction—notably complex I–linked abnormalities in dopaminergic neurons—has long been implicated in PD, although its causal ordering relative to α-synuclein (α-Syn) pathology and inflammation remains unsettled (Wright 2022). Irrespective of primacy, mitochondrial constraint decreases the safety margin for ionic homeostasis, vesicular cycling, transmitter clearance and activity-dependent plasticity—mechanisms that are bioenergetically expensive and centrally expressed in striatal microcircuits. Under such conditions, metabolites that report and remodel cellular energy state are positioned to become rate-limiting determinants of circuit stability.

Lactate is a plausible candidate for such a metabolite. Once framed as a terminal “waste” of anaerobic metabolism, lactate is now acknowledged as a mobile carbon substrate and signaling molecule in the nervous system, engaging in neuron–glia metabolic coupling, excitability tuning and plasticity-linked transcriptional programs (Magistretti and Allaman 2018). Beyond receptor-mediated and redox-dependent effects, lactate may also influence longer-timescale cellular states through lysine lactylation. This mechanism should be treated as emerging rather than established in PD striatal circuits (Mao et al. 2024). Histone and non-histone lactylation provide a plausible route by which sustained glycolytic or inflammatory lactate states could alter transcriptional and proteomic programs, but cell-type-resolved lactylome maps and causal perturbation studies in PD-relevant striatal microenvironments remain limited (Mao et al. 2024). This extended lactate repertoire is especially pivotal to PD because disease progression integrates energetic stress with chronic neuroinflammation—particularly under conditions in which lactate levels, lactate/pyruvate redox state, and lactylation-linked transcriptional “memory” may be altered.

Meanwhile, lactate biology is intrinsically bidirectional. Lactate can underpin tissue resilience via substrate delivery and adaptive signaling, but it can also engage in pathological regimes when associated with acidification, ionic instability and inflammatory metabolic reprogramming. Recent PD-model studies sharpen this duality by suggesting that excess brain lactate can contribute to parkinsonian phenotypes, whereas restoration of lactate homeostasis through upstream glycolytic control may be protective (Lian et al. 2024). Complementary work implicates glycolysis-derived lactate and microglial histone lactylation as potential contributors to pro-inflammatory activation in PD models, conceptualizing lactate not only as a marker of stress, but also as a possible determinant of cellular state in the neuroimmune compartment (Qin et al. 2025). These findings challenge any simplistic “lactate is beneficial” narrative and motivate a more rigorous, kinetics- and compartment-aware framework.

Exercise provides a particularly informative translational setting within this framework because it is among the best-supported interventions in PD and the most reproducible physiological perturbation of systemic lactate currently available. Early clinical trials, including SPARX and the ongoing SPARX3 program, have reinforced the idea that exercise intensity is not merely a training descriptor but a biologically meaningful signaling variable (Moore et al. 2013; Schenkman et al. 2018; Patterson et al. 2022). At the same time, lactate–HCAR1 signaling, angiogenic adaptation, and brain metabolic uptake studies provide a plausible mechanistic bridge between peripheral exercise physiology and central circuit regulation (Morland et al. 2017). Yet the central translational question remains unresolved: when exercise benefits PD, does lactate function as a causal mediator, a permissive metabolic context signal, or simply a correlated by-product of broader training adaptation?

This Review addresses that question by treating lactate not as a uniformly beneficial metabolite, but as a state-dependent variable whose significance depends on concentration, kinetics, compartment, and disease context. We integrate lactate generation and shuttling (Magistretti and Allaman 2018), D2-MSN-relevant striatal circuitry (Kravitz et al. 2010), adaptive lactate-linked substrate, HCAR1 and ERK/MAPK–CREB mechanisms (Magistretti and Allaman 2018), and maladaptive regimes involving acid–ion stress, neuroinflammation, dopamine disruption and possible lactylation-linked state locking (Lian et al. 2024). Importantly, we frame these mechanisms through a translational lens. Specifically, we consider whether lactate-related measures could be developed as biomarker-informed readouts of metabolic state, whether lactate kinetics could improve exercise dosing and mechanistic interpretation, and whether MCT- or HCAR1-directed strategies could provide actionable intervention nodes in PD.

**Fig. 1:**
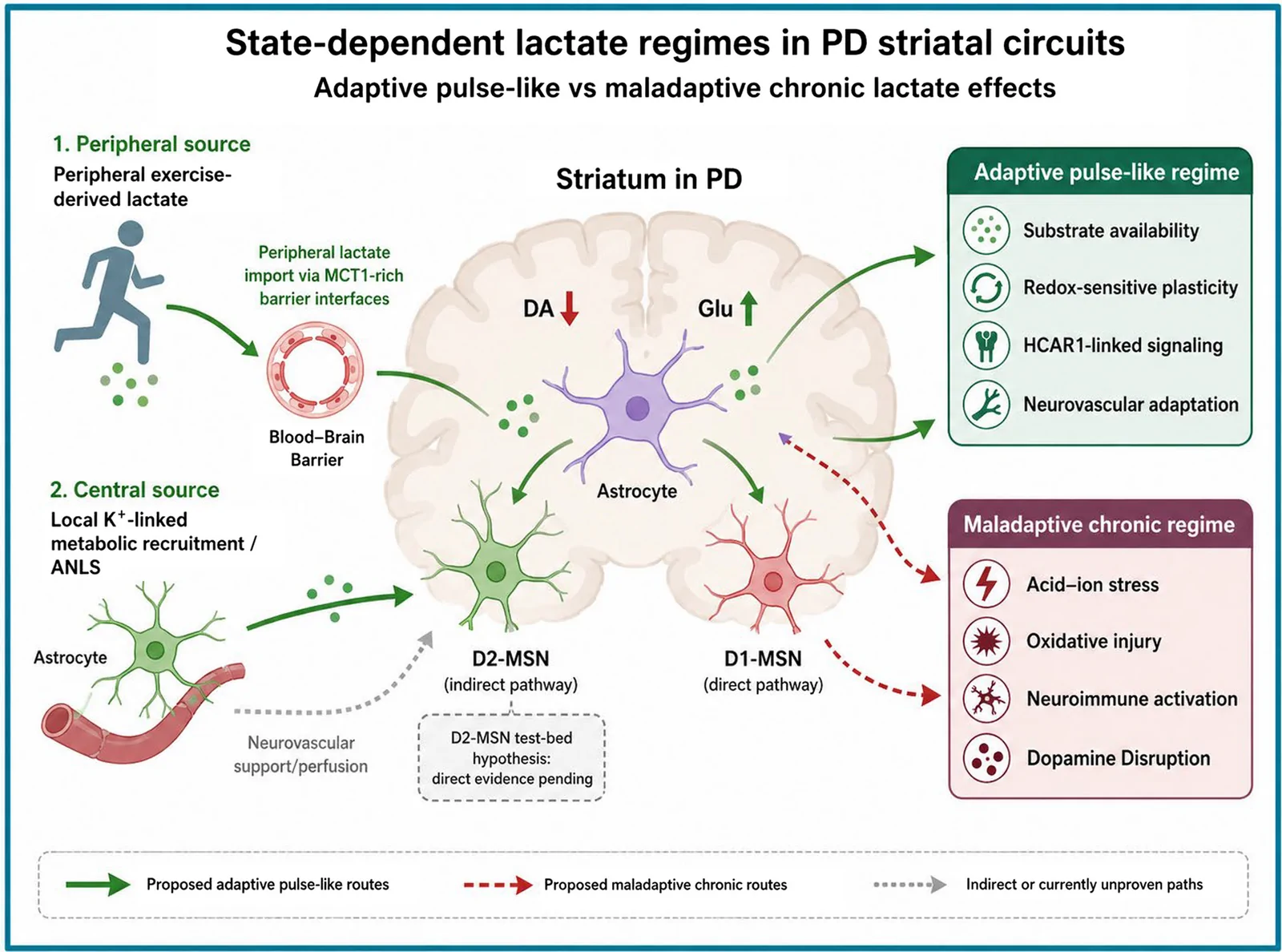
State-dependent lactate regimes in PD striatal circuits. Peripheral exercise-derived lactate and locally produced brain lactate are shown as distinct, potentially interacting sources. Peripheral lactate may enter the brain through MCT-rich blood–brain barrier interfaces, whereas local lactate production may arise from K + -linked astrocytic metabolic recruitment, glycogenolysis and neuron–glia exchange. In adaptive pulse-like regimes, transient and well-cleared lactate elevations may support substrate availability, redox-sensitive plasticity, HCAR1-linked receptor signaling and neurovascular adaptation. In maladaptive chronic regimes, sustained lactate accumulation coupled to mitochondrial constraint, impaired clearance, acid–base disequilibrium or inflammatory glycolytic reprogramming may promote ionic stress, oxidative injury, neuroimmune activation and disruption of dopaminergic signaling. The D2-MSN axis is shown as a prioritized experimental test bed rather than as a proven site of lactate-mediated rescue. Solid green arrows indicate proposed adaptive pulse-like routes; dashed red arrows indicate proposed maladaptive chronic routes; grey dotted arrows indicate indirect or currently unproven paths

A central theme running through the review is that translational progress will depend less on asking whether lactate is “good” or “bad” than on defining when lactate-related signals are adaptive, when they are destabilizing, and how these states can be measured and manipulated. This requires cell-type- and compartment-resolved causal testing in basal ganglia circuits—especially along the D2-MSN axis—as well as standardized perturbation paradigms capable of linking lactate dynamics to clinically meaningful endpoints. In that sense, lactate should be evaluated not only as a mechanistic concept, but also as a candidate bridge between metabolic theory, biomarker development, and precision rehabilitation in PD (Fig. 1).

For this narrative review, the literature was surveyed using PubMed, Web of Science, and Google Scholar up to January 2026. Search terms included combinations of “Parkinson’s disease”, “lactate”, “striatum”, “D2 medium spiny neurons”, “monocarboxylate transporter”, “HCAR1”, “exercise”, “biomarker”, and related terms. Priority was given to mechanistic studies, PD-relevant preclinical models, human translational studies, and articles directly informing lactate transport, signaling, metabolism, and biomarker development in PD. Evidence-tier convention. To avoid overinterpreting inferential links, mechanistic claims are labeled throughout using the following hierarchy: Tier 1, direct human PD evidence; Tier 2, direct PD-model evidence; Tier 3, non-PD neural or brain-slice evidence with plausible PD relevance; Tier 4, peripheral/exercise physiology evidence that defines exposure or feasibility but not PD causality; and Tier 5, hypothesis-generating inference. Unless explicitly labeled Tier 1 or Tier 2, claims about lactate effects on D2-MSNs, basal ganglia HCAR1 signaling, and lactylation-linked striatal remodeling should be read as testable hypotheses rather than established mechanisms.

## Lactate generation, shuttling, and cellular functions

### Sources of lactate: peripheral exercise versus central glycolysis

Lactate is produced via the near-equilibrium interconversion of pyruvate by lactate dehydrogenase (LDH), a reaction that integrates carbon flux to cytosolic redox balance by regenerating NAD⁺ from NADH (Ren et al. 2025). In this view, lactate is not an accidental end-product, but a programmable metabolite that enables high glycolytic throughput, supports rapid redistribution of reducing equivalents, and preserves metabolic homeostasis across cellular compartments and tissues (Ren et al. 2025). Accordingly, any discussion of lactate sources must be explicitly dynamic: lactate is produced continuously in many cell types even under aerobic conditions and rises when glycolytic flux exceeds local oxidative capacity or when metabolic signaling actively promotes glycolysis (Ren et al. 2025).

#### Peripheral lactate: systemic excursions induced by exercise

At the whole-body level, acute lactate excursions are driven predominantly by exercise, during which contracting skeletal muscle markedly accelerates glycolysis and releases lactate into the circulation (Ren et al. 2025). Contemporary exercise physiology emphasizes that the rise in blood lactate with increasing workload primarily reflects accelerated carbohydrate turnover and glycolytic flux, rather than serving as a simple proxy for tissue hypoxia (Mandadzhiev 2025). During maximal or near-maximal bouts, blood lactate can reach very high concentrations (commonly ~ 15–25 mM shortly after brief all-out efforts), illustrating how rapidly peripheral tissues can saturate clearance pathways and generate transient systemic hyperlactatemia (Goodwin et al. 2007).

The systemic lactate pool is also shaped by ubiquitous non-muscle sources that are constitutively glycolytic (for instance, erythrocytes), and by organs that remove lactate via oxidation and gluconeogenesis, particularly liver and kidney (Dashty 2013). Within the modern “lactate shuttle” framework, lactate acts as a mobile currency exchanged between producer (“driver”) and consumer (“recipient”) tissues—including skeletal muscle subtypes, heart, liver, kidneys, and brain—thereby linking glycolytic and oxidative metabolism into a coupled network instead of opposing modes (Brooks 2009). This systemic perspective is particularly important for neurodegeneration research because exercise generates precisely timed lactate pulses in the circulation that can be delivered to the brain, potentially engaging central circuits and gene programs that are otherwise difficult to modulate pharmacologically.

#### Entry of peripheral lactate into the brain: transport and oxidation

Peripheral lactate impacts the brain only insofar as it crosses the blood–brain barrier (BBB) and is metabolically or signaling-competent within neural tissue. Lactate transport across membranes is mediated by proton-linked monocarboxylate transporters (MCTs), with MCT1 functioning as a principal route at the BBB and additional isoforms contributing to parenchymal uptake and exchange (Halestrap and Wilson 2012). A human study provides direct evidence that the brain can switch from net lactate generation under basal conditions to net lactate uptake when plasma lactate is elevated, and that this uptake increases further during exercise (Hall et al. 2009). In an integrated lactate-infusion plus cycling paradigm, arterial lactate was experimentally clamped from ~ 0.9 mM at rest to ~ 3.9 mM during infusion and ~ 6.9 mM during exercise, accompanied by a shift toward cerebral lactate uptake and substantial oxidation of lactate-derived carbon (Hall et al. 2009). Complementing arterio-venous approaches, dynamic 13C MRS studies in humans have quantified plasma lactate transport and utilization in cortex, supporting the conclusion that circulating lactate can contribute measurably to oxidative brain metabolism, especially under conditions of elevated plasma lactate (Boumezbeur et al. 2010). Together, these data link peripheral lactate surges during high-intensity exercise to central lactate availability, a key premise for interpreting exercise effects on BG function in PD.

#### Central lactate: activity-coupled glycolysis and astrocytic glycogenolysis

Alongside peripheral delivery, the brain possesses robust endogenous lactate production capacity. Cellular-resolution models of neuroenergetics emphasize metabolic cooperation between neurons and astrocytes, with astrocytes functioning as dynamic managers of glucose uptake, glycolysis, and energy substrate distribution (Bélanger et al. 2011). A seminal finding supporting this view is the demonstration that glutamate uptake into astrocytes cues glycolysis and lactate production via Na⁺-dependent transport and activation of the Na⁺/K⁺-ATPase—offering a direct coupling between excitatory synaptic activity and astrocytic lactate generation (Pellerin and Magistretti 1994). Subsequent reviews have combined this concept into a comprehensive framework where lactate is formed primarily in astrocytes from glucose and, importantly, from astrocytic glycogen stores in response to neuronal activity signals (Magistretti and Allaman 2018).

Astrocytic glycogen constitutes a uniquely rapid, locally deployable carbohydrate reserve, and its mobilization can be functionally decisive during high-demand states. Experimental work has reported that astrocyte-specific glycogenolysis and lactate generation can be necessary for specific neurophysiological outputs, reinforcing the concept that lactate generation is not only a metabolic by-product, but can be an active node of circuit function (Hertz and Chen 2018). More broadly, central lactate transients track synaptic activity: pathophysiological syntheses stress that neuronal signals, such as K⁺ and glutamate can acutely stimulate astrocytic glycolysis, generating a transient lactate rise that can propagate beyond the activated microdomain (Barros 2013). This central source differs from peripheral lactate in two decisive ways: (i) it is spatially microdomain-structured (synapse- and circuit-adjacent), and (ii) it is temporally phase-locked to neurotransmission and ion-homeostatic work performed by glia, instead of to whole-body work rate.

#### Convergence during exercise: distinguishing import from local production

Exercise offers a natural experiment where peripheral and central lactate sources rise concurrently. In humans, 1H-MRS studies have detected enhanced brain lactate following vigorous exercise (for instance, a demonstrated ~ 19% increase in visual cortex lactate after graded exercise near ~ 85% predicted maximal heart rate), consistent with increased central lactate availability in the post-exercise period (Maddock et al. 2011). Additional ultra-high-field MRS work supports the detectability of post-exercise brain lactate changes, while also stressing methodological sensitivity as a limiting factor in quantifying small, transient lactate signals in vivo (Dennis et al. 2015). Importantly, these findings do not, on their own, resolve the relative contributions of BBB import versus local generation. A mechanistic interpretation therefore requires integrating plasma lactate kinetics, BBB transport capacity including MCT expression and trafficking, evidence for cerebral lactate uptake and oxidation under hyperlactatemia, and activity-dependent astrocytic lactate generation induced by synaptic signaling (Halestrap and Wilson 2012; Hall et al. 2009; Pellerin and Magistretti 1994). A further refinement comes from recent genetically encoded sensor work showing that mitochondrial lactate in neurons can track extracellular and circulating lactate with high fidelity. This finding strengthens the interpretation that peripheral lactate excursions can be represented within neuronal metabolic compartments, but it also complicates a simple “lactate import equals respiratory fuel” model. Mitochondrial lactate signals may report a combination of import, matrix redox state, lactate venting and mitochondrial protein lactylation rather than oxidation alone (Mao et al. 2024; Rauseo et al. 2026).

#### Implications for PD-focused striatal neuroenergetics

For PD, systemic lactate is a global exercise-linked signal, whereas central lactate is a local, circuit-synchronous substrate and signaling hub. These two sources may act synergistically—exercise elevates plasma lactate while simultaneously increasing neural activity and astrocytic metabolic engagement—but they may also diverge in disease states if BBB transport, astrocytic glycolytic responsiveness, or glycogen mobilization is impaired (Bélanger et al. 2011). Determining which source predominates in the striatum across PD stages and exercise intensities is therefore not a technical detail but a mechanistically central question: the same increase in tissue lactate could reflect beneficial substrate delivery, compensatory glial glycolysis, or a shift in redox state, each with distinct downstream consequences.

### The astrocyte–neuron lactate shuttle (ANLS) and its regulation

The astrocyte–neuron lactate shuttle (ANLS) is best treated here not as a universal rule of brain energy metabolism, but as a regulated capacity for activity-coupled lactate exchange. In the canonical model, astrocytes take up glucose, engage glycolysis or glycogenolysis, export lactate, and provide neurons with a substrate that can support oxidation and signaling programs (Magistretti and Allaman 2018). For PD, the key issue is not whether ANLS operates everywhere, but whether lactate flux becomes limiting or maladaptive in striatal microdomains exposed to dopamine loss, mitochondrial constraint, and altered synaptic demand.

Mechanistically, ANLS should be framed as activity-coupled metabolic recruitment rather than a strictly glutamate-driven shuttle. Glutamate-coupled astrocytic glycolysis remains relevant, but high-temporal-resolution work indicates that extracellular K⁺ is a dominant fast trigger, with glutamate and related signals acting more slowly or modulatory (Bittner et al. 2011; Barros et al. 2023; Madrer et al. 2025). This places the present Review within a broader metabolism-to-network framework in which ion-homeostatic work, not only transmitter recycling, drives local metabolic responses (Barros et al. 2025). Lactate exchange is then shaped by MCT isoform kinetics and localization: MCT1 can support exchange at barrier and glial interfaces, MCT2 is suited for high-affinity neuronal uptake, and MCT4 is adapted for lactate export under high-lactate conditions (Contreras-Baeza et al 2019). In PD striatum, therefore, a rise in tissue lactate could reflect peripheral import, K⁺-linked astrocytic recruitment, glial export, neuronal uptake, impaired oxidation, or defective clearance rather than a single directional shuttle.

Several experimental observations justify retaining ANLS as a functional mechanism. Disrupting astrocytic lactate production or transport impairs long-term potentiation and memory, and these deficits can be rescued by lactate, supporting a role for astrocyte-derived lactate in plasticity-linked programs (Suzuki et al. 2011). In vivo lactate sensor studies further show cell-resolved gradients and dynamics compatible with astrocyte-to-neuron lactate transfer under defined condition (Mächler et al. 2016). These data establish ANLS as a real physiological capacity, but they do not prove that astrocyte-to-neuron lactate transfer dominates in every brain region, stimulation regime, or disease state.

Regulation of this capacity occurs at several levels. Synaptic activity can recruit astrocytic glycolysis through glutamate uptake and ion-homeostatic work, linking local lactate generation to the energetic cost of transmission (Pellerin and Magistretti 1994). Astrocytic glycogenolysis provides a rapid reserve, and noradrenergic signaling can promote glycogen breakdown and lactate generation, making lactate supply sensitive to behavioral state and arousal (Jiang et al. 2020). Integrative models similarly emphasize that neuromodulatory inputs can mobilize astrocytic lactate export during heightened neural activity (Coggan et al. 2018). This is relevant to basal ganglia circuits because arousal, vigor, and neuromodulatory tone shape corticostriatal gain. Human transcriptomic–imaging associations also identify MCT2 expression as a candidate link between lactate handling and brain-state organization, supporting the idea that lactate transport capacity is not merely housekeeping metabolism (Medel et al. 2022).

Stress pathways and transporter trafficking add further control points. Astrocytic AMPK activity is required for normal astrocytic glycolysis and lactate generation, and its disruption can produce non-cell-autonomous neuronal dysfunction, implying that glial lactate support may become limiting during energetic challenge (Muraleedharan et al. 2020). At the membrane level, MCT abundance and localization constrain whether lactate is exported, imported, or allowed to accumulate (Pierre and Pellerin 2005). Basigin/CD147 is required for proper MCT surface expression and transport activity, meaning that lactate-shuttle competence depends not only on metabolic enzymes, but also on transporter trafficking (Muramatsu 2016). A critical refinement is that ANLS should not be interpreted as obligate neuronal lactate fueling. Neurons can increase their own glycolysis, lactate flux can reverse depending on gradients and redox state, and bulk lactate signals can reflect production, import, oxidation, export, or impaired clearance (Díaz-García et al. 2017). Evidence from awake-mouse glucose-imaging studies should also be interpreted cautiously because some approaches rely on bulky glucose analogs rather than native glucose transport (Lundgaard et al. 2015; Kovar et al. 2009). The most defensible position is therefore flux-based and context-dependent: astrocyte-to-neuron lactate transfer is a biologically meaningful capacity, but its dominance must be demonstrated for each circuit, disease state, and measurement paradigm rather than assumed or dismissed a priori (Magistretti and Allaman 2018).

For PD-focused striatal vulnerability, this framework has two implications. First, lactate delivery may be gated by neuromodulatory state and energy-sensing pathways, including AMPK, both of which are likely to shift during neurodegeneration (Muraleedharan et al. 2020). Second, “ANLS impairment” could arise from several distinct failures: altered activity-coupled astrocytic glycolysis, defective glycogen mobilization, impaired MCT trafficking, reduced neuronal lactate oxidation, or poor clearance (Pierre and Pellerin 2005). These mechanisms predict different downstream consequences for excitability, plasticity, inflammation, and metabolic stress. Accordingly, ANLS is retained in this Review as a context-dependent lactate-handling framework for testing PD striatal mechanisms, not as a settled explanation for lactate flux in D2-MSN-centered circuits.

### Lactate as fuel, signal, and epigenetic modifier

Across mammalian tissues, lactate is best recognized as a multifunctional intermediate instead of a terminal by-product: it is a mobile carbon substrate, a context-sensitive signaling cue and, increasingly, a precursor for covalent protein modifications that can remodel transcriptional programs. In the brain, these functions converge because lactate generation and exchange are embedded in activity-dependent neuroenergetics, while lactate sensing and downstream pathways map directly onto excitability and plasticity mechanisms. A useful organizing principle is hence not whether lactate is “good” or “bad”, but which molecular interface it engages—mitochondrial oxidation (fuel), membrane receptors and redox-sensitive ion channels (signal), or chromatin and protein lactylation (epigenetic/proteomic)—and under what temporal and concentration regimes. A substantial body of work supports lactate oxidation as a neuronal substrate and highlights neuroprotective actions in metabolic stress contexts (Magistretti and Allaman 2018). Meanwhile, both the magnitude and even the perspective of lactate flux between astrocytes and neurons appear state- and region-dependent, motivating a framework where lactate supports brain function via parallel metabolic and signaling routes instead of a single obligatory shuttle (Dienel et al. 2025).

#### HCAR1 (GPR81): a dedicated lactate receptor with limited basal ganglia evidence

Hydroxycarboxylic acid receptor 1 (HCAR1; GPR81) provides a receptor-defined route through which extracellular lactate can act as a signal rather than only as a carbon substrate. HCAR1 is canonically coupled to Gi/o proteins, inhibits adenylyl cyclase, and lowers cAMP-dependent signaling, making it a plausible sensor of elevated extracellular lactate during high-glycolytic, high-activity, or exercise-linked states (Colucci et al. 2023). However, its relevance to PD striatal circuitry should be interpreted cautiously because the strongest CNS evidence comes from general neuronal and neurovascular contexts rather than from basal ganglia-specific studies.

In vivo work indicates that lactate–HCAR1 signaling can promote VEGFA expression and cerebral angiogenesis, providing one mechanistic link between systemic lactate elevations and longer-term neurovascular adaptation (Morland et al. 2017). Neuronal studies further show that HCAR1 activation can dampen network activity and excitatory transmission, supporting the view that lactate can recruit receptor-defined inhibitory signaling when extracellular lactate is elevated (de Castro Abrantes et al. 2019). Structural studies have also clarified ligand recognition and receptor activation mechanisms, strengthening the pharmacological tractability of HCAR1 (Pan et al. 2025).

For this Review, HCAR1 is therefore treated as a validated lactate-sensing mechanism with plausible but unproven relevance to PD striatal circuits. Direct evidence that HCAR1 regulates basal ganglia output, D2-MSN excitability, beta synchronization, or parkinsonian motor behavior remains limited. Accordingly, HCAR1 should be viewed as a candidate signaling route for future testing, not as an established mechanism of lactate-mediated rescue in PD.

#### Lactate potentiation of NMDARs and activation of the ERK/MAPK pathway

Alongside GPCR signaling, lactate can engage synaptic plasticity machinery via redox-sensitive regulation of glutamatergic transmission. A seminal study demonstrated that L-lactate induces the expression of activity- and plasticity-related genes (including immediate-early genes, such as Arc, c-Fos and Zif268) through a mechanism requiring NMDA receptor (NMDAR) activity and the downstream ERK1/2 (MAPK) cascade; importantly, these effects were uncovered in neuronal cultures and in vivo (Yang et al. 2014). Pathophysiological dissection in this line of work supports a model where lactate modifies intracellular redox state—via its conversion to pyruvate and concomitant NADH production—thereby potentiating NMDAR function and amplifying Ca^2^⁺-dependent signaling into ERK activation (Yang et al. 2014).

This redox–NMDAR coupling offers an elegant bridge between metabolism and information processing: lactate availability, which reflects local glycolytic engagement and/or substrate delivery, becomes capable of tuning a receptor class that is central to synaptic integration and plasticity. A complementary transcriptomic approach demonstrated that lactate can induce broad gene-expression changes in neurons, with enrichment of plasticity-linked pathways and dependence on NMDAR activity (for instance, blockade by MK-801), reinforcing the view that lactate is not only permissive fuel, but can be instructive for neuronal gene programs (Margineanu et al. 2018).

An important nuance is that NMDAR potentiation and downstream signaling are not expected to be monotonic. Experimental work supports the idea that lactate may operate via separable mechanisms whose dominance depends on the strength and pattern of glutamatergic stimulation—consistent with a regime where lactate can enhance biological plasticity signaling at moderate activity, but may cause pathological amplification when excitatory drive is excessive (Jourdain et al. 2018). This duality is particularly relevant for BG circuits in PD, where glutamatergic drive, DA-dependent plasticity rules and ERK pathway engagement are all perturbed. In later sections, this framework will be used to interpret how lactate-dependent ERK/MAPK–CREB programs might support beneficial plasticity under exercise interventions yet also intersect with pathological plasticity phenotypes (for instance, dyskinesia-related signaling states).

#### Lactate-induced reactive oxygen species and hormesis

Because lactate oxidation feeds reducing equivalents into mitochondrial metabolism, it can modulate reactive oxygen species (ROS) production—sometimes as a liability, but also as a signaling intermediate. A landmark study in neuronal cell models and C. elegans showed that L-lactate can induce a mild ROS increase that activates cellular defense pathways (including NRF2-linked antioxidant responses and proteostasis programs, such as the unfolded protein response), thereby increasing resistance to subsequent oxidative insults—a classic hormetic logic where subtoxic stress primes resilience (Tauffenberger et al. 2019).

This hormesis framing aids reconcile seemingly contradictory findings in the literature. In several settings, lactate pretreatment is associated with decreased oxidative injury and improved mitochondrial integrity, consistent with either adaptive induction of protective programs or improved energetic support (Zou et al. 2023). In other settings—notably when lactate accumulates alongside acidification, weakened clearance or mitochondrial dysfunction—lactate-linked redox shifts could amplify oxidative stress instead of mitigate it, especially in cell types already operating near bioenergetic limits. Current syntheses in the lactylation–neurobiology literature emphasize this bidirectionality and argue that lactate can engage in either protective signaling loops or vicious cycles depending on cellular metabolic state and inflammatory context (Cao et al. 2025).

For PD, these distinctions matter because nigrostriatal degeneration and circuit dysfunction are accompanied by mitochondrial stress and neuroinflammatory activation, conditions that can shift the redox significance of lactate from adaptive messenger to damaging amplifier. The critical point for the present review is not that lactate is intrinsically antioxidative or pro-oxidative, but that it can set redox tone and engage hormetic programs—an effect that may be beneficial when appropriately dosed (for instance during controlled exercise) yet potentially maladaptive when lactate rises in parallel with ionic homeostatic collapse and inflammatory priming.

#### Lactate-induced epigenetic regulation: histone lactylation

The discovery of histone lysine lactylation (Kla) established a direct route whereby lactate metabolism can be incorporated into chromatin state. In the original report, lactate-derived lactylation sites were identified on core histones, and histone lactylation was reported to stimulate gene transcription, linking glycolytic metabolism to epigenetic regulation in a manner conceptually analogous to acetylation, but metabolically distinct (Zhang et al. 2019). This finding has two immediate implications for neurobiology. First, it expands lactate’s repertoire beyond short-lived signaling into possibly longer-lasting transcriptional reprogramming. Second, because lactate levels can rise under hypoxia, inflammation and high glycolytic states, lactylation provides a plausible mechanism whereby metabolic context can bias cellular identity and function.

Mechanistic details of “writing” and “erasing” lactylation marks remain an active area of exploration. Several reviews integrate evidence that canonical acyltransferase machinery, including p300/CBP, may engage in catalyzing lactylation in several contexts, although the current framework is still defining substrate forms, compartmental bottlenecks and the extent to which lactylation is enzymatically directed versus chemically permissive under high lactate conditions (Shi et al. 2025). In addition, the lactylation landscape is extending beyond histones to non-histone proteins, raising the possibility that lactate can remodel cellular function at multiple regulatory layers simultaneously (Peng and Du 2025).

In the central nervous system (CNS), evidence for functional histone lactylation is emerging, but remains comparatively sparse in comparison with immunology and cancer biology. Current CNS-focused reviews emphasize that lactate metabolism and lactylation are likely to influence neuroinflammation, glial state transitions and neuronal gene programs, but they also stress major gaps: cell-type-resolved maps of lactylation in vivo, causal tests linking lactylation to circuit-level phenotypes, and pathophysiological dissection of lactylation writers/erasers in brain-resident cells are still limited (Wang et al. 2024). These limitations are not only technical; they define the boundary between a plausible conceptual model (metabolism-to-chromatin coupling) and actionable therapeutic hypotheses.

For PD, lactylation is best framed as an emerging mechanism that may matter most during sustained metabolic and inflammatory dysregulation. The strongest PD-relevant evidence currently concerns microglial activation and inflammatory transcriptional programs, not direct lactylation-dependent remodeling of D1- or D2-MSN function. Accordingly, lactylation is retained in this Review as a lower-evidence, hypothesis-generating interface linking chronic lactate dysregulation to persistent cell-state changes. It should not be interpreted as co-equal in evidentiary strength with lactate transport, lactate oxidation, or redox-sensitive NMDAR–ERK signaling. The principal mechanistic interfaces through which lactate may influence striatal function in Parkinson’s disease are summarized in Table 1.

**Table 1:** Mechanistic interfaces through which lactate may influence striatal function in PD

Mechanistic interface	Main compartment / cell type	Adaptive regime	Maladaptive regime	Evidence tier for the claim in this Review	Decisive experiment	References
Peripheral lactate delivery to brain	Blood, BBB endothelium, brain parenchyma	Exercise-induced lactate pulses may increase central substrate availability and support active circuits when uptake and clearance are intact	Peripheral lactate elevation alone does not prove benefit; poor clearance or disease-state metabolic constraint may convert exposure into nonspecific stress	**Tier 3** for human brain lactate uptake/oxidation; **Tier 4** for exercise exposure; **Tier 5** for PD-specific mediation	Test whether blocking or clamping exercise-induced lactate selectively alters striatal circuit and motor benefits in PD models or patients	van Hall et al. (2009); Brooks (2018)
Astrocyte–neuron lactate shuttle / metabolic recruitment	Astrocytes, neurons, perisynaptic processes	K⁺-linked astrocytic metabolic recruitment, glycogenolysis, and MCT-dependent exchange may provide local energetic support during synaptic demand	Failure of glial glycolysis, glycogen mobilization, transporter trafficking, or neuronal oxidation may produce local energetic mismatch and vulnerability	**Tier 3** for non-PD neural mechanisms; **Tier 5** for PD striatal microdomain causality	Use cell-type-resolved lactate sensors and MCT perturbation to determine net lactate flux in PD striatum during defined activity states	Pellerin & Magistretti (1994); Bittner et al. (2011); Sotelo-Hitschfeld et al. (2015); Barros et al. (2023)
MCT-dependent lactate transport	BBB, astrocytes, neurons, endothelial cells	MCT1/MCT2/MCT4 expression and kinetics may enable lactate import, local exchange, and cell-type-specific substrate routing	Transporter mismatch, impaired trafficking, or excessive lactate export/import may promote local accumulation, pH stress, or inefficient substrate use	**Tier 3** for transporter biology; **Tier 5** for PD striatal causality	Manipulate MCT1, MCT2, or MCT4 selectively in PD-relevant striatal cell types while measuring lactate flux, pH, and motor output	Bergersen (2007); Contreras-Baeza et al. (2019)
Lactate as oxidative substrate	Neurons, astrocytes, mitochondria	Lactate conversion to pyruvate may support ATP generation and preserve neural activity during acute energetic constraint	Under mitochondrial dysfunction, lactate availability may fail to rescue energy demand and may instead track redox stress or impaired compensation	**Tier 3** for general neural energy support; **Tier 5** for identified PD striatal cell types	Demonstrate that physiological lactate transients improve ATP/redox resilience in identified D2-MSNs or striatal ensembles in PD models	Wyss et al. (2011); van Hall et al. (2009)
HCAR1-mediated signaling	Neurons, vascular-associated cells, possibly glia	Transient HCAR1 activation may dampen excessive activity, reduce cAMP signaling, and support neurovascular adaptation	Persistent or mislocalized HCAR1 engagement may reduce network gain, impair flexibility, or reinforce maladaptive hypokinetic states	**Tier 3** for receptor biology; **Tier 5** for basal ganglia, D2-MSN, and PD-specific causality	Test Hcar1 loss or activation in striatal cell types during PD-relevant lactate exposure, separating neuronal, vascular, and glial effects	Lauritzen et al. (2014); Morland et al. (2017); de Castro Abrantes et al. (2019)
Redox-sensitive NMDAR–ERK/MAPK–CREB signaling	Neurons, especially striatal MSNs	Lactate-linked redox shifts may potentiate NMDAR–ERK/MAPK–CREB programs and support activity-dependent plasticity when appropriately timed	Excessive glutamatergic drive or dyskinesia-prone D1-MSN ERK activation may convert lactate-sensitive plasticity into maladaptive signaling	**Tier 3** for non-PD neuronal mechanisms; **Tier 5** for D2-MSN-specific PD causality	Determine whether realistic lactate transients alter ERK timing, localization, and transcriptional output in genetically identified D2-MSNs without worsening D1-MSN dyskinetic signaling	Yang et al., (2014); Kravitz et al. (2010)
Lactate-linked mitochondrial redox / hormetic ROS signaling	Neurons, mitochondria, stress-response pathways	Mild lactate-linked redox changes or mitochondrial lactate venting may activate protective antioxidant and proteostasis programs	In impaired clearance or mitochondrial dysfunction, redox shifts may amplify oxidative injury rather than precondition against it	**Tier 3** for general redox biology; **Tier 5** for PD striatal causality	Map the boundary between protective redox signaling and injurious ROS amplification in PD-relevant striatal neurons and glia	Tauffenberger et al. (2019); Rauseo et al. (2026); Mao et al. (2024)
Histone / protein lactylation	Chromatin, glia, possibly neurons and mitochondria	Controlled lactate-linked lactylation may contribute to adaptive transcriptional or proteomic remodeling under defined metabolic states	Chronic glycolytic/lactate stress may stabilize inflammatory glial programs or other maladaptive cell-state transitions	**Tier 3** for general lactylation biology; **Tier 5** for PD striatal neuron or D2-MSN remodeling	Perform cell-type-resolved lactylome mapping and causal perturbation of lactylation writers/erasers in PD-relevant striatal microenvironments	Mao et al. (2024); Qin et al. (2025); Zhang et al. (2019); (Qiao et al. 2019)
Microglial inflammatory reprogramming	Microglia, neuroimmune compartment	Transient metabolic signaling may participate in stress adaptation, but adaptive roles remain poorly defined	Lactate excess and lactylation-linked pathways may reinforce pro-inflammatory microglial states and worsen circuit stability	**Tier 2** for PD-model microglial inflammatory relevance; **Tier 5** for human PD validation	Test whether altering lactate flux or lactylation directly changes microglial state, dopaminergic injury, and motor phenotype in PD models	Qin et al. (2025); Lian et al. (2024)
D2-MSN-centered metabolic neuromodulation	Indirect-pathway D2-MSNs, corticostriatal synapses	Lactate may support controlled inhibition, adaptive plasticity, and stabilization of indirect-pathway function in hypokinetic states	Depending on disease stage and signaling bias, lactate-associated mechanisms may weaken network flexibility or promote pathological synchronization	**Tier 2** for D2-MSN involvement in hypokinesia; **Tier 5** for lactate-specific D2-MSN modulation	Directly measure and manipulate lactate dynamics in genetically identified D2-MSNs while recording excitability, ERK signaling, beta coupling, and motor output	Kravitz et al. (2010); Parker et al. (2018) (Lucas et al. 2018)
Interaction with dopamine-dependent striatal energetics	Dopamine terminals, SPNs, ion pumps, second-messenger systems	Lactate may help sustain striatal computation when dopaminergic control is weakened but not absent	Dopamine depletion may lower the threshold at which lactate-related pH/redox perturbations destabilize circuit state	**Tier 3** for integrative striatal energetics; **Tier 5** for direct lactate–dopamine causal interaction in PD	Map how dopamine loss redistributes substrate use, lactate responsiveness, and pH/redox vulnerability across direct- and indirect-pathway SPNs	Lian et al. (2024); Kravitz et al. (2010)

Evidence-tier definitions: Tier 1, direct human PD evidence; Tier 2, direct PD-model evidence; Tier 3, non-PD neural or brain-slice evidence with plausible PD relevance; Tier 4, peripheral/exercise physiology evidence that defines exposure or feasibility but not PD causality; Tier 5, hypothesis-generating inference. When more than one tier is listed, the first tier refers to the established source evidence, whereas the higher-numbered tier refers to the PD-specific or D2-MSN-specific causal claim

Bold type within the table body indicates keyclassification labels or descriptors used to organize the table and does not indicate statistical significance

*Abbreviations*: *BBB *Blood–brain barrier, *BG* Basal ganglia, *CREB* cAMP response element-binding protein, *D2-MSN* D2 receptor-expressing medium spiny neuron, *ERK* Extracellular signal-regulated kinase, *HCAR1* Hydroxycarboxylic acid receptor 1, *MCT* Monocarboxylate transporter, *MSN* Medium spiny neuron, *NMDAR* N-methyl-D-aspartate receptor, *PD* Parkinson’s disease, *ROS* Reactive oxygen species, *SPN* Spiny projection neuron

## The striatum as a hub in PD motor circuitry

### Direct and indirect pathways and their imbalance in PD

The basal ganglia (BG) are embedded within recurrent cortico–BG–thalamo–cortical loops that regulate action selection, vigor, and motor scaling. In the classical pathway model, direct-pathway spiny projection neurons facilitate movement by reducing inhibitory output from GPi/SNr, whereas indirect-pathway neurons suppress movement through the GPe–STN–GPi/SNr route; the hyperdirect cortico–STN pathway provides an additional fast braking mechanism (Albin et al. 1989; Nambu et al. 2002). This circuit architecture remains essential for understanding PD, but it should not be reduced to a simple “Go/NoGo” switch.

Dopamine depletion alters the gain, plasticity, and temporal coordination of these pathways. Optogenetic and recording studies show that direct- and indirect-pathway neurons can be co-engaged during movement, while causal manipulations demonstrate that pathway bias can bidirectionally modulate parkinsonian motor behaviors (Cui et al. 2013; Kravitz et al. 2010). PD-related imbalance is therefore better viewed as a layered network disturbance involving altered pathway recruitment, hyperdirect braking, beta-band synchronization, and dopamine-dependent remodeling of corticostriatal synapses (Hammond et al. 2007; Mallet et al. 2008; Day et al. 2006).

For the present Review, the key implication is that striatal motor dysfunction is not only a dopaminergic signaling problem. It is also a state-control problem in which synaptic load, oscillatory coupling, neuromodulatory tone, and local energetic constraints interact. This provides the circuit rationale for asking whether lactate-related metabolic signals can stabilize or destabilize striatal computation under PD-relevant conditions.

### D2-MSNs as a high-priority cellular axis for understanding hypokinesia in PD

Medium spiny neurons (MSNs; also termed spiny projection neurons, SPNs) constitute the principal computational and output cell class of the striatum, transforming convergent cortical and thalamic excitation into BG output that finally gates movement. Within this population, D2 dopamine receptor–enriched MSNs (D2-MSNs) form the canonical striatopallidal node of the indirect pathway, projecting to the external globus pallidus (GPe) and thereby influencing subthalamic nucleus (STN) drive to the GPi/SNr output nuclei. This anatomical position is where a hypokinetic phenotype can be expressed: increased striatopallidal recruitment increases downstream inhibitory output and decreases thalamocortical enhancement of action, offering a parsimonious circuit substrate for bradykinesia and akinesia (Zhai et al. 2018).

Several established observations justify using D2-MSNs as the main cellular entry point for this Review. First, indirect-pathway D2-MSNs are positioned to suppress or constrain motor output, but they do so as part of coordinated action selection rather than as a passive “NoGo” channel. Cell-type-resolved recordings show that direct- and indirect-pathway neurons can be co-active around action initiation, indicating that D2-MSNs help sculpt motor output, competing actions, and movement vigor rather than simply blocking movement (Cui et al. 2013).

In addition, D2-MSNs are causally linked to hypokinetic behavior. Selective activation of indirect-pathway MSNs can suppress movement, whereas direct-pathway activation can facilitate movement and rescue motor deficits in dopamine-depleted mice (Kravitz et al. 2010). Dopamine loss also shifts D2-MSN plasticity rules and corticostriatal synaptic organization, including altered D2 receptor–dependent cAMP/PKA signaling, impaired endocannabinoid-dependent LTD, and cell-type-biased synaptic remodeling (Day et al. 2006; Shen et al. 2008; Kreitzer and Malenka 2007; Lerner and Kreitzer 2012).

Moreover, D2-MSNs connect molecular plasticity to network dynamics and clinical leverage. Parkinsonian models show abnormal recruitment of indirect-pathway neurons into beta-linked hypokinetic states, while A2A receptor antagonism provides a clinically actionable non-dopaminergic route converging on striatopallidal neurons (Sharott et al. 2017; Zemel et al. 2022; Yu et al. 2009; Isaacson et al. 2022; Chen and Cunha 2020). These points justify the D2-MSN focus, while caveats remain: D1/D2 boundaries are not absolute, hybrid SPN populations exist, and dopamine depletion remodels both pathways (Zhai et al. 2018; Bonnavion et al. 2024). Thus, D2-MSNs are emphasized here because their role in hypokinetic BG output is well supported, not because lactate-specific mechanisms have already been demonstrated in this cell type.

This D2-MSN-centered framework provides a concrete experimental substrate for testing the review’s central hypothesis: that lactate-related mechanisms could influence the same dimensions through which D2-MSNs gate hypokinesia, including intrinsic excitability, synaptic plasticity thresholds, and state-dependent recruitment into synchronized network regimes (Shen et al. 2008). Accordingly, later sections will treat D2-MSNs not only as an anatomical “indirect pathway component”, but as a cell-type-defined metabolic decision point where the same lactate signal could buffer circuit computation (adaptive plasticity and controlled inhibition) or weaken pathological inhibition (pathological synchronization and rigid network states), depending on disease stage and metabolic context (Zhai et al. 2018).

### Striatal energy metabolism and dopaminergic control

The striatum sits at a metabolic crossroads: it integrates dense excitatory input from cortex and thalamus into sparse but behaviorally powerful inhibitory output, while continuously adjusting synaptic weights and excitability in a dopamine-dependent manner. This computation is energetically demanding because a large fraction of brain ATP use is devoted to restoring ion gradients perturbed by synaptic transmission and action potentials (Rumpf et al. 2023). triatal spiny projection neurons (SPNs/MSNs) amplify this constraint: they usually operate near a hyperpolarized down-state, but must rapidly transition to spiking during convergent synaptic barrages on densely spiny dendrites (Plenz and Wickens 2016). Thus, striatal energy demand is driven less by average firing rate than by transient episodes of synaptic integration, dendritic electrogenesis, plasticity induction, K⁺ handling, and transmitter cycling (Carter et al. 2007). The striatum is also metabolically distinctive, with high resting glucose utilization but comparatively low extracellular glucose availability, implying a narrow safety margin when synaptic activity, dopamine terminal work, or pathological synchronization increases local demand (Lowry et al. 1998).

This energetic load is compartmentalized. Dendritic spines, shafts, soma, dopamine terminals, and glial microdomains experience different patterns of Na⁺, K⁺, and Ca^2^⁺ flux and therefore may rely on different mixtures of oxidative phosphorylation, glycolysis, and metabolite exchange. Although current in vivo tools cannot yet resolve all of these subcellular fluxes in behaving PD models, mitochondrial perturbation studies support the view that striatal circuits are particularly vulnerable when oxidative capacity is compromised (Pickrell et al. 2011). This matters for PD because mitochondrial stress may reduce the reserve needed to support ion pumps, vesicle cycling, glutamate handling, and activity-dependent plasticity during high-demand network states.

2Dopamine signaling itself is metabolically embedded. Dopamine release, vesicle recycling, and dopamine transporter-mediated clearance depend on ion gradients and ATP-consuming membrane processes (Sulzer et al. 2016). Slice studies further suggest that dorsal and ventral striatal dopamine release may differ in their reliance on oxidative versus glycolytic support, although these ex vivo findings should be interpreted as capacity constraints rather than direct measures of in vivo flux (Msackyi et al. 2024). Dopamine also shapes energy demand indirectly by controlling SPN excitability and synaptic integration: D1 receptor signaling tends to enhance dendritic excitability and glutamatergic integration in direct-pathway SPNs, whereas D2 receptor signaling exerts opposing effects in indirect-pathway SPN (Surmeier et al. 2007). Because excitatory conductance and Ca^2^⁺ signaling are major drivers of ATP use, these electrophysiological effects necessarily redistribute metabolic burden. More directly, dopamine can suppress Na⁺/K⁺-ATPase activity in neostriatal neurons through coordinated D1 and D2 receptor involvement, linking dopaminergic tone to one of the dominant neuronal ATP-consuming processes (Bertorello et al. 1990).

Recent in vivo work further shows that dopamine-dependent second-messenger signaling is coupled to metabolic state. Locomotion can activate PKA in SPNs through interactions between dopamine release and acute adenosine accumulation, effectively linking neuromodulatory control to a purinergic signal that reflects ATP turnover and local energetic demand (Ma et al. 2022). This is relevant to exercise and PD because the same dopamine transient may have different circuit consequences depending on concurrent metabolic load, adenosine tone, and substrate availability.

Human metabolic imaging provides useful but limited insight into these processes. FDG-PET studies identify reproducible PD-associated metabolic patterns and relate them to symptoms and treatment responses (Meles et al. 2017). Contemporary reviews note that PD often shows relative hypermetabolism in subcortical nodes, including basal ganglia and thalamus, although results depend on normalization strategy, disease stage, and comorbidity (Nerattini et al. 2025). Two interpretive limits are essential. First, FDG-PET measures glucose uptake and phosphorylation, not ATP production, mitochondrial efficiency, or partitioning among glucose, lactate, and other substrates (Meles et al. 2017). Second, basal ganglia hypermetabolism should not be equated with better function; it may reflect compensatory drive, pathological synchronization, gliosis, heightened inhibitory output, or inefficient network states that consume more energy while transmitting less useful information (Matthews et al. 2018).

The current evidence therefore does not yet define how dopamine depletion redistributes metabolic flux across D1- and D2-SPNs, striatal subregions, glial compartments, or subcellular domains. Links among dopamine-controlled cAMP/PKA signaling, ion pump regulation, mitochondrial performance, adenosine tone, and substrate choice remain partly inferred and require testing in intact behaving systems (Ma et al. 2022). For this Review, the cautious synthesis is that striatal energetic bottlenecks are computationally coupled, mitochondrially limited, and dopamine-shaped (Pickrell et al. 2011). This provides the rationale for considering lactate in PD: as a fuel, redox modulator, and receptor ligand, lactate could influence substrate availability, redox tone, and neuromodulatory signaling in circuits already operating close to energetic and dynamical limits (Ma et al. 2022).

## Lactate-dependent support of striatal function: beneficial mechanisms

### Lactate as an alternative energy substrate

A central premise of lactate biology in the brain is that lactate is not only a glycolytic overflow product, but a circulating and locally produced carbon source that can be oxidized by neural tissue when demand, availability, or metabolic state shifts. Human biological studies establish that the brain is capable of substantial lactate uptake and oxidation when plasma lactate is elevated, shifting from net lactate generation at rest to net uptake during hyperlactatemia and exercise. This capacity is mechanistically plausible given high-capacity monocarboxylate transport across the BBB and into parenchyma, and it is functionally relevant because lactate can rise swiftly in the circulation during high-intensity exercise and in the brain interstitium during intense synaptic activity or stress (Hall et al. 2009).

For striatal circuits, the alternative-substrate framing is especially consequential. Striatal output is shaped by brief epochs of high synaptic integration and plasticity induction superimposed on low baseline spiking, meaning that “energetic sufficiency” is often decided on short time scales and within limited microdomains. In such regimes, lactate may act as a buffer substrate that preserves oxidative ATP production when glucose delivery, glycolytic throughput, or mitochondrial capacity becomes rate-limiting—yet this benefit is expected to be conditional on intact transport, redox handling, and mitochondrial function, and hence must be argued with evidence-tier transparency.

#### Metabolic flexibility and energetic rescue under stress

Two complementary human approaches offer direct support that circulating lactate contributes to oxidative brain metabolism. First, arterio–venous balance integrated with lactate infusion and exercise shows that the brain takes up lactate and oxidizes it, with uptake increasing markedly as arterial lactate is experimentally raised (to ~ 4 mM) and further during exercise (reported ~ 7 mM) (Hall et al. 2009). Second, dynamic 13C MRS utilizing labeled lactate demonstrates brain entry and metabolic incorporation of lactate-derived carbon into amino acid pools linked to the TCA cycle, allowing quantification of transport and oxidative utilization in humans (Boumezbeur et al. 2010). Together these studies build a capacity for lactate-supported oxidation in vivo, especially during systemic lactate elevations.

The most conceptually rigorous illustrations of lactate as an “alternative substrate” come from paradigms where glucose availability is experimentally constrained. In healthy humans, lactate infusion during insulin-induced hypoglycemia protected aspects of cerebral function and modified counterregulatory and symptomatic responses, consistent with lactate functioning as an auxiliary fuel when glucose is insufficient (Maran et al. 1994). A strong in vivo demonstration of lactate’s ability to support neural function under energy crisis comes from severe hypoglycemia experiments in rodents, where lactate infusion preserved neuronal activity when glucose availability was critically reduced (Wyss et al. 2011). However, this evidence also requires mechanistic caution: modeling and spectroscopy indicate that lactate’s benefit may derive not only, or not primarily, from replacing glucose oxidation, but from maintaining or restoring glucose oxidation and overall metabolic activity under hypoglycemic constraints (Wyss et al. 2011). This evidence does not prove that lactate mediates exercise benefit in PD, but it establishes that circulating lactate can become functionally relevant to neural activity when glucose-dependent metabolism is constrained.

These illustrations are strong, but they are not automatically transferable to the striatum or to PD. Most human lactate-oxidation measurements are cortical, and ex vivo/rodent models often involve systemic manipulations that do not map neatly onto chronic neurodegeneration (Boumezbeur et al. 2010). In addition, lactate oxidation depends on mitochondrial competence; hence, in PD states defined by mitochondrial stress (especially complex I–linked vulnerabilities), the marginal ATP gain from lactate may be decreased or may be altered toward redox imbalance instead of net rescue. This motivates a cautious conclusion: lactate is a viable alternative oxidative substrate for brain under specific conditions, but whether it rescues striatal computation in PD is a testable hypothesis, not a settled inference.

#### Neuroprotective mechanisms linked to lactate metabolism

An especially instructive study tested lactate in both in vitro and in vivo ischemia models. In rat organotypic hippocampal slices, adding 4 mM L-lactate immediately after oxygen–glucose deprivation decreased neuronal death, in contrast to 20 mM was toxic—providing direct evidence of a narrow therapeutic window even in acute settings (Berthet et al. 2009). In mice subjected to middle cerebral artery occlusion and reperfusion, intracerebroventricular lactate administered immediately after reperfusion decreased lesion size and improved neurologic outcome, with a more pronounced reduction observed in the striatum—a notable point for PD because it highlights that striatal tissue can be lactate-responsive in vivo under energetic crisis (Berthet et al. 2009). The same study also demonstrated timing sensitivity: delayed administration (1 h after reperfusion) did not reduce lesion size, but still improved neurological outcome, meaning separable lesion-volume and functional mechanisms (Berthet et al. 2009). These findings support a pathophysiological interpretation where lactate oxidation preserves ATP availability during a critical post-insult window, thereby encouraging ion pumps (notably Na⁺/K⁺-ATPase), limiting depolarization-driven glutamate release, and preserving glial glutamate clearance—mechanisms that are plausible, but importantly, not uniquely attributable to lactate (other swiftly oxidizable substrates might achieve similar effects). The dose-dependent toxicity further cautions against a simplistic “more lactate is better” narrative (Berthet et al. 2009).

Translational interest in lactate as a metabolic support therapy is reinforced by controlled clinical work in traumatic brain injury (TBI). A randomized controlled trial compared half-molar sodium lactate with saline in severe TBI and evaluated prevention of raised intracranial pressure episodes, confirming that exogenous lactate can be deployed as a brain-directed metabolic/osmotherapy strategy in humans (Ichai et al. 2013). Nevertheless, interpretation needs care: sodium lactate is hypertonic and modifies osmolarity and sodium load, so beneficial effects cannot be cleanly assigned to lactate oxidation alone. The pathophysiological signal is nonetheless important for neurodegeneration: these data report that increasing systemic lactate availability can modify brain physiology in humans, encouraging—but not proving—the plausibility of lactate-supported energetic stabilization under stress (Ichai et al. 2013).

Neuroprotection by lactate metabolism must be evaluated against emerging PD-specific data suggesting that lactate homeostasis can itself become pathological. A recent PD model study reports that increasing “lactate homeostasis” via SIRT1-induced inhibition of PKM2 delayed parkinsonian phenotypes, and that elevating lactate in brain could recapitulate PD-like features—an observation that proposes for a potential detrimental regime of excess lactate in chronic disease contexts (Lian et al. 2024). This is not a contradiction of acute neuroprotection; rather, it underscores context dependence: lactate may rescue during acute energetic failure yet cause chronic maladaptation when production, clearance, or downstream signaling become dysregulated. The implication for this review’s framework is that “lactate-as-fuel” similarly has a therapeutic window set by concentration, timing, and the integrity of oxidative metabolism.

#### Relevance to exercise interventions in PD

Exercise is one of the most reproducible non-pharmacological interventions for PD motor function, and high-intensity endurance exercise is feasible and has reported signal for motor benefit in de novo PD in a phase 2 randomized clinical trial (SPARX) (Schenkman et al. 2018). High-intensity exercise is also the biological condition that most consistently elevates circulating lactate, and human physiology shows that the brain increases lactate uptake and oxidation as plasma lactate rises, including during exercise (Hall et al. 2009). This convergence offers a coherent hypothesis: exercise-generated lactate pulses could phasically augment cerebral oxidative substrate supply, possibly encouraging striatal circuits during high-demand states and engaging lactate-sensitive signaling pathways discussed in Sects. "Lactate as fuel, signal, and epigenetic modifier" through "D2-MSNs as a high-priority test bed for lactate-sensitive striatal mechanisms".

The evidence chain remains incomplete in PD specifically. SPARX establishes feasibility and clinical signal for high-intensity exercise, but it does not identify lactate as a mediator (Schenkman et al. 2018). Meanwhile, PD models provide mixed guidance on lactate handling: one study reports unaltered levels of MCT1/MCT2 and GLUT1 at the BBB and in striatum/SN in the MPTP mouse model, suggesting that monocarboxylate transport capacity may be preserved—at least in that model and timeframe—supporting feasibility of lactate utilization (Puchades et al. 2013). In contrast, the SIRT1–PKM2 study suggests that excessive lactate production/accumulation may aggravate PD-like pathology, raising the possibility that exercise-linked lactate signaling must be dosed and interpreted within disease stage and metabolic state (Lian et al. 2024).

The most defensible position, given current data, is that lactate is a credible candidate contributor to exercise benefits, but not a proven mediator in PD. The near-term agenda is to quantify striatal lactate kinetics during exercise, test whether MCT or oxidation blockade attenuates exercise benefit, and separate substrate, vascular, and signaling mechanisms because each predicts different risk profiles. The comprehensive literature already shows that lactate can be neuroprotective in acute metabolic crisis with a narrow dose window and that systemic lactate can alter brain physiology in humans; PD-specific work now suggests that chronic lactate homeostatic collapse could be pathogenic (Berthet et al. 2009). Reconciling these strands is specifically why lactate is best framed as a dynamic substrate: beneficial when it increases energetic flexibility, harmful when it signals or enforces pathological metabolic states.

### Lactate-driven gene programs and synaptic plasticity via ERK/MAPK–CREB

A recurring theme in contemporary neuroenergetics is that lactate can become instructive—not only permissive—by engaging signaling pathways that couple synaptic activity to transcriptional and translational programs. Among these, the ERK/MAPK–CREB axis is a plausible convergence point because it is (i) exquisitely sensitive to Ca^2^⁺ entry and redox state, (ii) a canonical gateway to activity-dependent immediate-early gene expression, and (iii) a known determinant of plasticity and memory-related consolidation processes. The most direct experimental support comes from primary neuronal systems where L-lactate cues plasticity-related gene expression via NMDAR-dependent ERK1/2 activation, uncovered both in culture and in vivo cortex (Yang et al. 2014). Importantly, the current framework also includes data suggesting that lactate effects can be context-dependent and, in several paradigms, separable into fuel/redox versus receptor-induced components—an ambiguity that should be kept explicit when extrapolating to striatal pathophysiology.

#### Mechanisms of ERK/MAPK activation by lactate

The most mechanistically resolved pathway linking lactate to ERK activation is redox-induced potentiation of NMDAR signaling. In the study by Yang and colleagues, L-lactate enhanced expression of multiple immediate-early genes (for instance Arc, c-Fos, Zif268/Egr1) through a cascade that required NMDAR activity and ERK1/2 signaling (Yang et al. 2014). A core inference from these experiments is that lactate metabolism (lactate → pyruvate) shifts intracellular redox balance (NADH/NAD⁺), thereby potentiating NMDAR-induced currents and Ca^2^⁺ entry upstream of ERK activation (Yang et al. 2014). This model is attractive because it provides a direct molecular route from “metabolic state” to a receptor class that is central to synaptic integration and plasticity.

Alongside redox-coupled mechanisms, lactate can signal via HCAR1/GPR81 and possibly recruit ERK signaling via non-canonical GPCR effectors (including β-arrestin scaffolds), at least in several cell contexts. for instance, lactate has been demonstrated to enhance Arc/Arg3.1 expression in astrocytes through an HCAR1–β-arrestin2 pathway, meaning that lactate can engage ERK-adjacent signaling architectures beyond Gi–cAMP suppression (Ma et al. 2020). Nevertheless, evidence for a dominant HCAR1 → ERK → CREB route in neurons remains less consistent than the NMDAR/redox route, and the relative contribution of these mechanisms probably varies with lactate concentration, cell type, and stimulation regime. A cautious synthesis is that lactate can access ERK via multiple entry points, and current data do not justify treating any single mechanism as universal.

A persistent interpretive challenge is that exogenous lactate manipulations can co-vary with pH, osmolarity and Na⁺ load (for instance, sodium lactate preparations), and that lactate’s metabolic conversion is tightly associated with redox state. Hence, a pathophysiological claim of “lactate signaling” needs explicit controls that delineate lactate per se from (i) acid–base effects and (ii) redox-driven modulation by downstream metabolites (such as pyruvate or NADH). The literature itself reflects this: transcriptomic analyses and follow-up experiments emphasize that NADH can mimic lactate’s gene-induction effects and that NMDAR blockade abrogates them (Margineanu et al. 2018).

#### CREB activation and activity-dependent gene expression

CREB phosphorylation and CRE-dependent transcription are classical components of long-term plasticity and memory consolidation, acting downstream of Ca^2^⁺- and kinase-driven signaling cascades. Lactate intersects this axis in two complementary ways. First, when lactate amplifies NMDAR/Ca^2^⁺ signaling and ERK activation, CREB becomes an expected downstream integrator via ERK-dependent kinases (for instance MSK/RSK) that target Ser133 phosphorylation. Second, lactate availability can become necessary for the molecular signatures of consolidation that include CREB activation. An especially influential demonstration of necessity comes from the astrocyte–neuron lactate transport work by Suzuki and colleagues: blocking glycogenolysis or lactate transport weakened long-term memory and disrupted associated molecular changes, including induction of phospho-CREB and Arc (Suzuki et al. 2011). While these data do not prove that lactate’s only role is CREB regulation, they provide strong evidence that lactate supply is upstream of CREB-linked consolidation programs in the condition tested.

Even when CREB phosphorylation is uncovered, transcriptional engagement depends on the spatiotemporal profile of ERK signaling. Current work on synapse-to-nucleus signaling supports the idea that maintained ERK signaling is required to induce CREB-dependent transcription after neuronal stimulation, beyond initial phosphorylation events that can be supported by multiple kinase pathways (Zent and Dell'acqua 2025). This principle is important when interpreting lactate effects: lactate may not simply “turn on” ERK/CREB, but may remodel the duration and nuclear coupling of ERK signals, thereby biasing whether transient activity is converted into lasting gene expression.

#### Plasticity- and survival-related gene networks regulated by lactate

The most direct evidence that lactate induces a synchronized plasticity gene program is the observation that L-lactate cues multiple activity-regulated genes (including Arc, c-Fos, Zif268/Egr1) via NMDAR-ERK1/2 dependence in neurons and in vivo cortex (Yang et al. 2014). These genes sit at the interface of synaptic tagging, structural remodeling and circuit consolidation, offering a credible route whereby lactate could modulate plasticity beyond acute energetics. Genome-wide approaches support this perspective while also sharpening its limits. An RNA-seq study in cortical neurons demonstrated that lactate induces a transcriptomic response enriched for immediate-early genes and MAPK-related pathways, reinforcing the concept of a lactate-sensitive transcriptional module (Margineanu et al. 2018). Yet these data are mainly derived from simplified systems (cultures; acute exposure), and hence cannot by themselves establish that the same network is engaged in vivo in a region- and cell-type-specific manner (for instance within striatal D2-MSNs in PD).

A second, highly visible lactate-responsive module involves BDNF signaling. In a mouse study examining exercise biology, lactate was identified as an exercise-associated metabolite that crosses the BBB and promotes hippocampus-dependent learning and memory in a BDNF-dependent manner (Hayek et al. 2019). Mechanistically, this work implicated SIRT1 and downstream transcriptional coactivator pathways (PGC-1α/FNDC5) as part of lactate’s route to Bdnf induction (Hayek et al. 2019). Here, critical caution is warranted. BDNF induction is a convergent endpoint for multiple exercise-linked mediators and neural activity patterns; lactate’s ability to facilitate Bdnf in one experimental setting does not imply that lactate is the sole—or even dominant—mediator of exercise-induced plasticity across brain regions or disease states. In addition, numerous lactate paradigms elevate lactate to millimolar levels; whether biological microdomain lactate transients achieve comparable signaling in vivo, especially in BG circuits, remains incompletely resolved.

The ERK pathway is a well-established determinant of striatum-dependent learning and plasticity. For instance, genetic perturbation of ERK1 modifies striatum-related memory and potentiation in nucleus accumbens, stressing that ERK-family signaling has causal leverage in BG plasticity (Mazzucchelli et al. 2002). This does not illustrate lactate → ERK signaling in striatum directly, but it strengthens the logic for why lactate-sensitive ERK engagement could plausibly influence striatal learning rules—an issue that becomes central when discussing PD and dysregulated corticostriatal plasticity.

#### Synapse-specific modulation and energy-demanding cellular processes

Lactate’s influence on plasticity is unlikely to be spatially uniform: synapses differ in receptor composition, metabolic coupling, and astrocytic coverage. A main electrophysiological study demonstrated that extracellular lactate (1–2 mM) can induce potentiation at specific hippocampal synapses (recurrent collateral synapses of CA3 pyramidal neurons), supporting the idea that lactate can bias plasticity in a circuit- and synapse-dependent manner (Herrera-López et al. 2020). While hippocampal, this is an important proof-of-principle: lactate effects can be synapse-selective instead of global, consistent with microdomain lactate signaling.

Long-term plasticity needs not only transcription, but also bioenergetically expensive de novo protein synthesis. One study directly tested whether lactate derived from astrocytic glycogenolysis fuels learning-induced neuronal translation. It demonstrated that lactate shuttling into neurons through MCT2 supports learning-induced de novo translation and Arc/Arg3.1 induction, and that alternative oxidative substrates (pyruvate or β-hydroxybutyrate) can rescue memory deficits caused by blocking glycogenolysis or astrocytic lactate transport—whereas equicaloric glucose could not, in that paradigm (Descalzi et al. 2019). This work is notable because it reframes lactate’s “plasticity support” as, at least in part, an energetic enabling of translational machinery instead of exclusively receptor-level signaling.

Collectively, the main evidence supports a model where lactate can (i) amplify NMDAR-dependent ERK signaling via redox coupling, (ii) engage in consolidation-linked CREB activation and immediate-early gene induction, and (iii) enable energy-intensive transcription/translation programs required for durable plasticity (Descalzi et al. 2019). Meanwhile, three limitations must be carried forward explicitly: much of the pathophysiological work is hippocampal/cortical instead of striatal; lactate manipulations can conflate lactate with pH/redox/metabolite effects; and the balance between “fuel” and “signal” mechanisms is not yet quantitatively resolved in vivo (Margineanu et al. 2018). These bottlenecks do not weaken the lactate–ERK/MAPK–CREB hypothesis; rather, they define the critical experiments needed to establish its relevance to PD, namely, cell-type-resolved demonstrations in striatal SPNs (especially D2-MSNs) that lactate transients engage ERK/CREB within specific concentration–time windows and with measurable consequences for corticostriatal plasticity.

### HCAR1 signaling in PD-relevant striatal circuits: plausible but unproven

Lactate can rise rapidly in extracellular space during high glycolytic demand, positioning it as a potential activity-linked signal rather than only a metabolic substrate. One receptor-defined route for this signaling is HCAR1/GPR81/HCA1. Direct evidence from non-basal-ganglia preparations shows that HCAR1 activation can suppress excitatory transmission and decrease neuronal firing in an HCAR1-dependent manner (Lauritzen et al. 2014). Mechanistically, HCAR1 is a class A GPCR canonically coupled to Gi/o proteins, leading to inhibition of adenylyl cyclase and reduced intracellular cAMP–PKA signaling. In primary cortical neurons, HCAR1-dependent downmodulation of activity requires Giα signaling and involves the adenylyl cyclase–cAMP–PKA axis (Lauritzen et al. 2014).

Anatomically, HCAR1 has been mapped in relation to synaptic and neurovascular compartments, supporting the idea that lactate may function as a volume-transmitted metabolic signal linking neuronal activity, extracellular lactate, and vascular responses (Lauritzen et al. 2014).Exercise-related work further shows that HCAR1 is enriched in vessel-associated cell populations and that lactate–HCAR1 activation can elevate VEGFA and induce cerebral angiogenesis, suggesting a role in longer-timescale neurovascular adaptation rather than only acute excitability control (Morland et al. 2017). Recent cryo-EM structures of HCAR1–Gi complexes have also clarified ligand recognition and agonist selectivity, strengthening the pharmacological tractability of the receptor (Pan et al. 2025). However, receptor druggability should not be equated with demonstrated PD relevance.

The best-supported functional output of HCAR1 is inhibitory modulation. In cortical neurons, selective HCAR1 activation decreases miniature EPSC frequency and increases paired-pulse ratio, consistent with reduced presynaptic glutamate release probability (Alessandri et al. 2024). Seizure-relevant studies similarly show that HCA1R activation decreases spontaneous EPSC frequency and alters paired-pulse metrics in wild-type but not knockout mice (Skwarzynska et al. 2023). HCAR1 activation can also reduce intrinsic firing responsiveness and spontaneous calcium spiking, while HCAR1 knockout neurons show higher basal activity (de Castro Abrantes et al. 2019). These findings support a general CNS model in which extracellular lactate can recruit receptor-mediated negative feedback during elevated activity states.

HCAR1 does not operate as an isolated switch. It can interact functionally with other Gi/o-coupled inhibitory systems, including adenosine A1, GABA_B and α2A-adrenergic receptors, suggesting that lactate signaling may reinforce existing inhibitory tone under metabolically demanding conditions (de Castro Abrantes et al. 2019). In hyperexcitable networks, this has been proposed as a lactate-dependent feedback loop: intense activity accelerates glycolysis, extracellular lactate rises, and HCA1R activation suppresses firing and excitatory transmission (Skwarzynska et al. 2023). This provides useful proof-of-principle for lactate–HCAR1 circuit stabilization, but epilepsy-like hyperexcitability should not be directly mapped onto PD basal ganglia dysfunction.

Several limitations are critical. Lactate manipulations can co-vary with pH and osmolarity, making genetic and receptor-selective approaches essential for assigning effects specifically to HCAR1 (de Castro Abrantes et al. 2019). In addition, HCAR1 localization and function are cell-type and region dependent. Some studies support neuronal receptor expression, whereas others emphasize astrocytic or vascular localization; hypothalamic work further indicates that lactate can alter neuronal activity even when HCAR1 is primarily expressed in astrocytes (López et al. 2023). Thus, not all lactate effects should be interpreted as direct neuronal HCAR1 actions.

For PD-relevant basal ganglia circuits, HCAR1 should therefore be treated as a candidate mechanism, not an established pathophysiological pathway. The rationale for retaining it is coherent but indirect: HCAR1 can reduce cAMP-linked signaling and excitability in non-BG preparations; it is present in brain synaptic and vascular-associated compartments; and lactate–HCAR1 signaling can mediate selected exercise-linked neurovascular adaptations (Morland et al. 2017; Colucci et al. 2023; de Castro Abrantes et al. 2019). These findings justify testing HCAR1 in striatal circuits, but they do not demonstrate that HCAR1 regulates basal ganglia output, D2-MSN excitability, beta synchronization, or parkinsonian motor behavior.

A conservative PD working model is therefore conditional. Transient lactate pulses could, in principle, engage HCAR1 to modulate corticostriatal gain, limit excessive excitation, or support neurovascular adaptation during metabolically demanding states. Conversely, persistent receptor engagement during chronic lactate elevation could reduce network gain, impair flexibility, or reinforce maladaptive hypokinetic states. Decisive evidence will require in vivo measurement showing that striatal lactate reaches receptor-relevant concentrations in PD models, cell-type-specific manipulation of Hcar1 in identified striatal populations, and behavioral readouts capable of separating D2-MSN, D1-MSN, vascular, and glial mechanisms.

### Exercise-induced lactate and PD striatal function: an indirect causal chain

The exercise–lactate–PD link should be read as an indirect causal chain with a missing mediator step. The established links are: exercise improves PD-relevant motor and striatal phenotypes; higher-intensity exercise produces larger systemic lactate excursions; circulating lactate can enter and be oxidized by the human brain; and lactate can engage substrate, receptor, vascular and redox-sensitive signaling mechanisms. The missing link is direct proof that lactate elevations are necessary or sufficient for exercise-induced striatal rescue in PD. Therefore, the evidence supports lactate as a candidate contributor and exposure marker, not as an established mediator.

#### Associations among exercise, lactate, and symptom improvement

In de novo PD, the SPARX phase 2 randomized trial established feasibility and safety of treadmill exercise at 80–85% of measured maximal heart rate and demonstrated that the high-intensity arm met a predefined nonfutility threshold for motor outcomes in comparison with usual care (Schenkman et al. 2018). While lactate was not measured, the design is informative for lactate biology because maintained high-intensity intervals are the condition most consistently associated with substantial systemic lactate excursions in humans; Hence, SPARX supports a dose–response premise compatible with lactate-induced mechanisms without proving them.

In the Park-in-Shape randomized controlled trial (neuroimaging subset), aerobic exercise (stationary cycling) enhanced functional connectivity of the anterior putamen with sensorimotor cortex in comparison with posterior putamen, and improved cognitive control, in contrast to stretching did not (Johansson et al. 2022). This is a crucial association for the present review because it anchors “exercise benefit” in a circuit-relevant striatal node (putamen) that is central to motor impairment in PD. Again, lactate was not quantified; the pathophysiological inference is hence indirect, but the anatomical specificity strengthens the case for searching for metabolic mediators that can influence synaptic gain and plasticity in this loop.

In a PD mouse model, aerobic training decreased striatal beta-band power and decreased MSN firing frequency, with an especially prominent normalization in D2-MSNs; the same study demonstrated increased striatal D2R expression after training (Wang et al. 2024). In pathophysiological terms, these outcomes map tightly onto the hypothesized striatal bottleneck for hypokinesia (pathological indirect-pathway recruitment and beta synchronization). The limitation is interpretive: the study demonstrates a striatal circuit rescue with exercise, but does not identify which exercise-evoked molecular signals (lactate among them) are causal.

In MPTP mice, intensive treadmill training increased striatal D2 receptor expression, as measured by synaptoneurosome immunoblotting and radioligand-based approaches (Vučković et al. 2010). In humans with early/mild PD, six months of high-intensity exercise enhanced DA transporter (DAT) availability in substantia nigra in most participants and demonstrated a more variable increase in putamen; the authors interpret these changes as improved function of remaining dopaminergic neurons, while acknowledging the need for randomized trials to validate and optimize regimens (Laat et al. 2024). These dopaminergic associations are relevant because lactate is positioned to influence DA release energetics and striatal excitability states (Sects. "The striatum as a hub in PD motor circuitry" through "D2-MSNs as a high-priority test bed for lactate-sensitive striatal mechanisms"), but the present data do not yet delineates lactate effects from other “exerkines” or from activity-dependent plasticity induced by repeated motor practice.

In humans, arterial–venous balance with tracer approaches shows that the brain increases lactate uptake and oxidation as blood lactate rises, including during exercise, with a concomitant decrease in net glucose uptake (Hall et al. 2009). This is an essential enabling observation: if exercise-derived lactate is to engage in striatal regulation, it must access the brain in meaningful flux, and human physiology confirms that it can.

#### Evidence compatible with lactate contribution, but not PD mediation

The most rigorous causal evidence in the “exercise → lactate → brain change” chain comes from HCAR1 genetics. Morland and colleagues demonstrated that exercise or systemic L-lactate enhanced hippocampal VEGFA levels in wild-type mice, in contrast to neither intervention elevated VEGFA above baseline in Hcar1 knockout mice—supporting a direct requirement for lactate receptor signaling in this exercise-linked angiogenic program (Morland et al. 2017). They further demonstrated that lactate/HCAR1 stimulation engages intracellular signaling (including ERK1/2 and Akt phosphorylation) in wild-type, but not knockout tissue, providing pathophysiological continuity from receptor engagement to growth-factor output (Morland et al. 2017). Although the readout was hippocampal, the principle generalizes: lactate can serve as an upstream causal signal for brain structural support mechanisms that plausibly benefit metabolically limited circuits, including BG.

In mice, work identifying lactate as an exercise-generated metabolite demonstrated that lactate crosses the BBB and promotes hippocampus-dependent learning and memory via BDNF-dependent signaling (Hayek et al. 2019). This establishes sufficiency for a central plasticity-relevant endpoint (BDNF signaling) in at least one brain system. The core caveat for PD is anatomical: hippocampal sufficiency does not automatically imply striatal sufficiency, because striatal plasticity rules and neuromodulatory regimes differ and PD introduces DA-dependent bottlenecks that may alter how lactate is interpreted.

To date, the strongest PD-relevant evidence remains associative: exercise rescues striatal D2-MSNs hyperactivity and beta signatures, and changes striatal DA receptor expression (Wang et al. 2024). What is mainly absent are experiments that selectively block lactate entry (MCT inhibition/conditional knockdown), lactate sensing (cell-type-specific Hcar1 deletion), or lactate oxidation in striatal neurons during exercise training and then measure whether striatal electrophysiology and motor outcomes still improve. Without these necessity tests, it is premature to label lactate as “the” mediator of exercise benefits in PD; the most defensible interpretation is that lactate is a high-priority candidate mediator with validated causal functions in other brain adaptations.

#### Multi-mechanistic synergy encouraging striatal performance

A lactate-centric model is most plausible when framed as **synergy** instead of monocausality. Exercise simultaneously perturbs substrate availability, neuromodulator tone, vascular function and synaptic activity patterns; lactate may intersect each layer.

Striatal computation is shaped by synaptic integration and ionic homeostasis instead of maintained firing. Lactate can buffer oxidative metabolism during high-demand epochs (Sect. " Lactate as an alternative energy substrate"), and the striatum shows swift extracellular glucose and lactate fluctuations tightly associated with neuronal activation, demonstrating that lactate availability is dynamically linked to synaptic demand in this region (Forderhase et al. 2020). In this framing, exercise-induced lactate pulses could reduce the probability that energy bottlenecks push striatal microdomains into pathological states (depolarization burden, weakened pump function, or redox stress), thereby indirectly supporting DA-dependent plasticity.

Lactate’s receptor-induced inhibitory logic (HCAR1/Gi) provides a plausible negative-feedback mechanism to dampen hyperexcitability and constrain pathological synchronization (Sect. " HCAR1 signaling in PD-relevant striatal circuits: plausible but unproven"). This is conceptually aligned with the observation that aerobic training can reduce striatal beta power and preferentially normalize D2-MSNs firing in PD models (Wang et al. 2024). Whether HCAR1 is the operative brake in striatum during exercise remains to be shown, but the pathophysiological “shape” matches the phenotype: a metabolite that rises during high effort, capable of recruiting inhibitory signaling, could help re-establish a healthier dynamic range in cortico-striatal transmission.

Exercise reshapes striatal signaling pathways implicated in pathological plasticity in PD and dyskinesia, including ERK/MAPK modules in MSNs (Sects. "Lactate-driven gene programs and synaptic plasticity via ERK/MAPK–CREB" and "D2-MSNs as a high-priority test bed for lactate-sensitive striatal mechanisms"). In a 6-OHDA PD mouse study, aerobic exercise reversed elevated Erk1/2 and p-Erk1/2 signals in dorsal striatum with greater effects in PENK-associated (indirect-pathway–linked) MSN signatures than PDYN-associated signatures (Wang et al. 2022). Separately, lactate/HCAR1 signaling can engage ERK1/2 and Akt in brain tissue in an Hcar1-dependent manner (Morland et al. 2017). These findings motivate a testable synergy model: lactate pulses could contribute to the tuning of ERK-dependent transcriptional responses while repeated motor practice offers synapse-specific activity patterns that “select” which synapses consolidate.

The lactate–HCAR1–VEGFA axis provides one of the cleanest molecular bridges from exercise to durable improvements in substrate delivery (Morland et al. 2017). For BG circuits, which are vulnerable to mitochondrial stress and demand–supply mismatch, incremental improvements in microvascular support could enhance resilience and improve the metabolic headroom needed for plasticity and movement scaling.

Exercise can upregulate dopaminergic markers and/or release capacity in PD models and humans (D2R in MPTP mice; DAT and putamen signals in early PD cohorts) (Vučković et al. 2010). Lactate could contribute by encouraging the energetic cost of DA release/clearance and by shaping striatal excitability states that gate DA effectiveness, but the perspective of causality remains open because DA circuits themselves can alter glycolysis and lactate generation.

Several PD model studies suggest that maintained lactate accumulation or enhanced glycolysis can be pathogenic—promoting dopaminergic vulnerability and neuroinflammation—such that decreasing lactate accumulation alleviates pathology in several settings (Qin et al. 2025). This literature forces an essential refinement of the “exercise-lactate benefit” hypothesis: benefit is likely to depend on lactate kinetics (transient pulses vs chronic elevation), cellular compartment (astrocytic export vs inflammatory microglial glycolysis), and the integrity of oxidative metabolism. A mature model hence predicts a therapeutic window: lactate signaling/substrate support may be adaptive when associated with intact clearance and mitochondrial handling, but pathological when lactate becomes a persistent marker of metabolic reprogramming and inflammatory activation.

The most defensible current conclusion is that exercise improves PD-relevant striatal outcomes, including putaminal connectivity and D2-MSNs-linked circuit state (Johansson et al. 2022; Wang et al. 2024), and that lactate is a biologically credible candidate contributor and exposure marker rather than a proven mediator. The supporting evidence is indirect: the human brain can import and oxidize lactate during exercise (Hall et al. 2009), and lactate receptor signaling is required for selected exercise-induced cerebral adaptations (Morland et al. 2017), but no PD study has yet shown that lactate is necessary or sufficient for exercise-induced striatal rescue. The decisive next step is to close the causality gap in BG utilizing cell-type-resolved lactate sensors and targeted blockade (MCTs/HCAR1/oxidation) during exercise, with readouts that match PD-relevant striatal phenotypes (D2-MSN excitability, beta synchronization, corticostriatal plasticity rules, and motor vigor). This unresolved mediator step leads directly to an apparent paradox: lactate may support neural function during acute exercise-like metabolic demand, yet sustained lactate elevation has also been implicated in PD-like pathology.

### The lactate paradox in PD: adaptive pulses versus chronic homeostatic collapse

A central tension in this field is that lactate can support neural function under acute metabolic demand, yet PD-model studies report that excessive brain lactate and disrupted lactate homeostasis can worsen parkinsonian phenotypes. In severe hypoglycemia models, circulating lactate can preserve neuronal activity when glucose availability is critically reduced, supporting the idea that lactate can become functionally relevant during acute energetic crisis (Wyss et al. 2011). By contrast, in PD model mice, SIRT1-mediated inhibition of PKM2 reduces lactate production and glial activation, whereas intracerebral lactate administration can recapitulate PD-like phenotypes, indicating that excessive brain lactate may participate in parkinsonian progression under pathological conditions (Lian et al. 2024). This is not a contradiction if lactate is treated as a kinetic and compartmental variable rather than as a molecule with a fixed biological sign.

Adaptive lactate regimes are typically pulse-like, time-limited and coupled to intact clearance. During vigorous exercise, blood lactate can rise over minutes and is subsequently cleared through oxidation, gluconeogenesis and inter-organ lactate shuttling (Brooks 2018). Under hyperlactataemic conditions, the human brain can shift toward lactate uptake and oxidation, as shown by lactate-infusion and exercise paradigms in which systemic lactate availability increased cerebral lactate utilization (Hall et al. 2009). In this regime, lactate may act as an oxidizable substrate, a redox-sensitive plasticity signal, and a trigger for vascular or receptor-mediated adaptation. Lactate can induce plasticity-related gene expression through NMDAR–ERK1/2 signaling in neurons, supporting its role as a redox-sensitive plasticity signal (Yang et al. 2014). Lactate receptor activation can also promote cerebral VEGFA expression and angiogenesis in mice, supporting a receptor-mediated neurovascular adaptation route (Morland et al. 2017). These effects remain indirect for PD unless lactate manipulation is shown to mimic, block or scale the exercise effect in PD models or patients.

Maladaptive lactate regimes are different. In PD-relevant models, sustained lactate accumulation appears in the setting of glycolytic reprogramming, mitochondrial constraint, inflammatory activation or impaired clearance. The SIRT1–PKM2 study directly supports this pathological side of the paradox by showing that reducing PKM2-driven lactate production attenuates glial activation and parkinsonian phenotypes, whereas brain lactate administration worsens PD-like features (Lian et al. 2024). In a complementary PD model, enhanced glycolysis-derived lactate promoted microglial activation through a P300/CBP-dependent H3K9 lactylation–SLC7A11 pathway, linking lactate excess to neuroinflammatory persistence rather than adaptive substrate support (Qin et al. 2025). Here, lactate is less likely to represent useful substrate availability and more likely to mark a collapsed redox/pH/ion-homeostatic state. Such a state may promote ROS–inflammasome coupling, microglial activation, lactylation-linked inflammatory persistence and dysregulated dopaminergic signaling. Recent sensor-based work further complicates the interpretation of lactate accumulation by showing that neuronal mitochondria can contain a dynamic lactate pool and that mitochondrial lactate venting may limit oxidative stress, indicating that lactate signals may reflect redox pressure relief as well as substrate flux (Rauseo et al. 2026).

The therapeutic window therefore cannot be defined by lactate concentration alone. It requires at least four dimensions: magnitude, duration, compartment and accompanying stress context. The key unresolved transition zone is the point at which repeated beneficial pulses become poorly cleared accumulation. Future PD studies should report lactate peak, area under the curve, clearance slope, lactate/pyruvate ratio, pH or bicarbonate context, and cell-type-resolved brain readouts where feasible. Without these variables, “high lactate” and “lactate benefit” are under-defined categories. Table 2 outlines Resolving the lactate paradox in PD.

**Table 2:** Resolving the lactate paradox in PD

Regime	Lactate kinetics	Likely source	Context	Expected effect	Evidence tier	Representative supporting evidence
Exercise-like pulse	Minutes-to-hours, cleared during recovery	Skeletal muscle import plus local activity-coupled production	Preserved perfusion, intact clearance, training adaptation	Candidate substrate/signaling support	Tier 3–4; indirect for PD mediation	Blood lactate can rise during exercise and serve as brain energy source; supports neuronal activity via NMDAR–ERK and HCAR1–VEGFA pathways (Morland et al. 2017; Hall et al. 2009; Yang et al. 2014; Brooks 2018; Wyss et al. 2011)
Pathological accumulation	Sustained or poorly cleared	Mitochondrial constraint, glycolytic reprogramming, inflammatory activation	Acid–ion stress, ROS, impaired dopamine handling	Maladaptive inflammatory and circuit state	Tier 2 in PD models	PD-model studies link excessive lactate to glial activation and dopaminergic injury; lactate promotes microglial activation via histone lactylation (Lian et al. 2024; Qin et al. 2025; Rauseo et al. 2026)
Transition zone	Repeated pulses with impaired clearance	Mixed peripheral and central sources	Stage-, age-, and comorbidity-dependent	Therapeutic window unknown	Tier 5; needs direct testing	Inference from contrast between acute lactate-support studies and chronic PD-model lactate dysregulation studies; no direct PD study has yet defined the transition from adaptive pulses to maladaptive accumulation

Evidence tiers: Tier 1 = direct human PD evidence; Tier 2 = direct PD-model evidence; Tier 3 = non-PD neural evidence with plausible PD relevance; Tier 4 = peripheral or exercise physiology evidence defining exposure or feasibility but not PD causality; Tier 5 = hypothesis-generating inference

## Lactate-related disruption of striatal function: detrimental mechanisms

### Acidification and disruption of ionic homeostasis

The most common pathophysiological slippage in “lactate toxicity” narratives is the implicit equation of lactate with acidosis. Biochemically, lactate production per se is not synonymous with proton generation; rather, proton accumulation during metabolic stress is shaped by ATP hydrolysis and by failures of proton-consuming oxidative phosphorylation, while lactate often rises Meanwhile as glycolytic flux increases (Robergs et al. 2004). Meanwhile, lactate and pH are tightly linked at the membrane level because monocarboxylate transporters (MCT1–4) move lactate together with a proton, allowing lactate flux to redistribute acid equivalents across cellular compartments and between cells (Geistlinger et al. 2023). In the striatum—where excitability depends on steep ionic gradients and where DA handling imposes additional redox and metabolic constraints—pathological acidification can hence become functionally dominant even when lactate itself is not the main acid source.

#### Pathological acidification: formation and consequences

Tissue acidosis emerges when glycolytic ATP turnover outpaces mitochondrial proton consumption (or when mitochondrial capacity is weakened), causing net proton accumulation (Robergs et al. 2004). In acute energy failure states, such as cerebral ischemia, brain pH can fall into a regime that is well within the activation range of proton-sensitive effectors (including ASIC channels), with demonstrated tissue pH values reaching ~ 6.0 in the ischemic core and ~ 6.5–6.9 in penumbral regions in animal stroke models (Larkin et al. 2021). Classic microelectrode work demonstrated that extracellular pH changes during complete ischemia covary with tissue lactate burden, encouraging the view that severe glycolytic states couple lactate accumulation to extracellular acidification (Katsura et al. 1991).

Acidification is not a single “toxin”, but a broad systems perturbation. Shifts in extracellular and intracellular pH alter (i) ion channel gating and driving forces, (ii) vesicular and transporter kinetics, and (iii) enzyme activities that maintain homeostasis (Larkin et al. 2021). Importantly, pH effects are often compartment-specific: extracellular protons can directly gate proton-sensing channels and modulate synaptic transmission, in contrast to intracellular protons remodel metabolic enzyme function and redox chemistry and can constrain ROS-generating pathways (Wang et al. 2015). This duality matters for pathophysiological interpretation: the same “acidification” event can phasically suppress several excitotoxic cascades while simultaneously impairing the pumps and transporters that prevent catastrophic ionic collapse.

#### Impairment of Na⁺/K⁺-ATPase and ion transport

The Na⁺/K⁺-ATPase (NKA) is the principal energetic sink that preserves neuronal ionic gradients and thereby preserves resting membrane potential, action potential repolarization, and secondary transport (including neurotransmitter uptake). Small changes in its function can hence produce disproportionate circuit consequences. Two pathophysiological routes connect lactate-associated acidification to NKA compromise:

NKA ion binding and selectivity are pH-sensitive, because protonation state changes core coordinating residues and modifies Na⁺/K⁺ handling at the cytoplasmic and extracellular faces of the enzyme (Cornelius et al. 2018). While such biophysical studies are not designed as disease models, they establish that acid–base shifts are sufficient to reparameterize pump behavior in principle.

In a striatum-focused study utilizing synaptosomal preparations, experimentally induced “lactic acidosis” at ischemia-relevant pH produced a progressive decrease in high-affinity DA uptake accompanied by decreased NKA activity; antioxidant treatment prevented the NKA loss, implicating oxidative mechanisms as contributors to pump impairment under acid stress (Barrier et al. 2004). This is conceptually important for PD, because it ties acidification not only to generic ionic stress, but also to DA handling—a uniquely striatal vulnerability axis.

A rigorous review must also acknowledge a methodological caveat: numerous “lactate” manipulations in vitro are operationally acid load manipulations (lactic acid titration or sodium lactate with buffering effects), so attributing outcomes to lactate signaling instead of pH needs explicit controls (matched pH, matched osmolarity) and, ideally, genetic or pharmacological dissection of lactate transport versus proton effects (Geistlinger et al. 2023).

#### Ionic imbalance, excitotoxicity, and downstream injury cascades

Once NKA capacity is reduced—or ATP supply is insufficient to sustain it—ionic homeostasis fails in a predictable, but destructive sequence: intracellular Na⁺ rises, membrane potential depolarizes, voltage-gated Ca^2^⁺ entry increases, and glutamate clearance and vesicular cycling become weakened, increasing the probability of excitotoxic signaling and oxidative injury. In striatum, an experimentally tractable demonstration comes from in vivo microdialysis studies where lactic-acid perfusion was used to induce a local “lactacidosis” resembling ischemic pH; this manipulation produced a biphasic rise in extracellular DA accompanied by elevations in extracellular glutamate and hydroxyl radical signals, and these effects were prevented by NMDA receptor blockade (MK-801) and by an antioxidant (Trolox), directly linking acid stress to glutamatergic excitotoxicity and oxidative cascades in striatal tissue (Remblier et al. 1999).

Acidosis can also facilitate injury via proton-gated ion pathways that bypass glutamate receptors. ASIC1a is activated by extracellular acidosis and can cause Ca^2^⁺ loading and neuronal injury; in a seminal in vivo ischemia study, pharmacological blockade of ASIC1a and ASIC1a knockout were protective even in the presence of NMDA receptor blockade, encouraging an acidotoxic pathway that is at least partly independent of classical excitotoxicity (Xiong et al. 2004). Mechanistically, extracellular protons can also trigger regulated cell-death programs through ASIC1a-associated signaling; for instance, an eLife study demonstrated that tissue acidosis can induce neuronal necroptosis through ASIC1a-linked mechanisms (Wang et al. 2015).

A critical nuance for pathophysiological honesty is that acidification is not uniformly pro-injury. Intracellular acidification can suppress NMDA-induced superoxide generation and neuronal death in culture by inhibiting pH-sensitive ROS-generating processes, indicating that modest pH shifts can be acutely protective in some contexts (Lam et al. 2013). The net outcome hence depends on where protons accumulate (extra- vs intracellular), how severe the pH fall is, and how long it persists—variables that differ substantially between acute ischemia models and chronic neurodegeneration.

#### Specific vulnerability of the Parkinsonian striatum

Several convergent considerations make the PD striatum plausibly more susceptible to acid–ion homeostasis interactions.

A recent human 31P-MRS study in early PD demonstrated that elevated putaminal Pi/ATP ratios—interpreted as weakened oxidative phosphorylation—were prominent in the posterior putamen and suggested that bioenergetic dysfunction at striatal dopaminergic terminals may exceed that in midbrain at early disease stages (Payne et al. 2024). Independent work has emphasized mitochondrial dysfunction (including complex I-linked abnormalities) as a core feature of PD pathobiology (Wright 2022). Even modest reductions in ATP supply can disproportionately endanger ion pumps and pH regulators, because these systems sit upstream of excitability control and transmitter clearance.

The striatum is abnormally sensitive to condition that simultaneously perturb pH, oxidative balance, and DA handling. The synaptosome study demonstrating that acidification decreases DA uptake alongside NKA impairment provides a mechanistic bridge from metabolic/acid stress to modified extracellular DA dynamics (Barrier et al. 2004). In vivo striatal lactacidosis experiments further report that acid stress can induce DA accumulation together with glutamate release and oxidative signals, suggesting a plausible route to feed-forward injury: ionic stress → transmitter homeostatic collapse → excitotoxic/oxidative cascades (Remblier et al. 1999).

In PD, lactate may rise as a compensatory substrate and signal (Sects. "Lactate as an alternative energy substrate" through "HCAR1 signaling in PD- relevant striatal circuits: plausible but unproven"), but if lactate dynamics are accompanied by extracellular acidification—via MCT-linked proton flux and/or weakened clearance—then the system can cross into a regime where pH-driven pump and transporter dysfunction dominate circuit behavior (Geistlinger et al. 2023). The strongest evidence for these acidotoxic cascades currently comes from ischemia and experimental lactacidosis models instead of chronic PD, so it is premature to claim that striatal acidification is a main driver of PD progression. The more defensible, testable proposition is that PD-associated metabolic insufficiencydecreases the threshold at which lactate-associated acid/ion disturbances destabilize striatal computation, especially under stressors that elevate glycolytic flux or weaken mitochondrial proton handling (Payne et al. 2024).

Overall, acidification is best framed as a conditionally dominant mediator of lactate-associated harm: it can convert metabolic compensation into ionic collapse when ATP supply is constrained, and the PD striatum—by virtue of its dopaminergic terminal metabolic insufficiencyand the tight coupling of DA handling to oxidative and transporter function—may sit tighter to this threshold than numerous other brain regions.

### Oxidative stress, mitochondrial dysfunction, and neuroinflammation related to lactate homeostatic collapse

A rigorous discussion of “lactate homeostatic collapse” in PD must delineate signal from symptom. Lactate accumulation can be a readout of limited oxidative metabolism (for instance, when mitochondrial capacity is weakened and glycolytic flux compensates), but lactate can also become a causal modulator of redox tone, mitochondrial electron transport, and immune signaling. The pathophysiological challenge is that these functions are entangled: the same upstream insult that elevates lactate (mitochondrial dysfunction) also increases reactive oxygen species (ROS) and can sensitize microglia, making it easy to misattribute causality. A PD-relevant synthesis hence needs to specify which lactate-linked variable is operating (absolute lactate, lactate/pyruvate ratio, lactate kinetics, or lactylation), where (neurons, astrocytes, microglia, endothelium), and when (acute stress versus chronic remodeling).

#### Lactate as a marker of mitochondrial constraint in PD-relevant states

Mitochondrial dysfunction—notably complex I–linked vulnerability in dopaminergic neurons—remains a central pillar of PD biology, although its causal position in comparison with α-syn pathology and other main catalysts is still controversial (Wright 2022). Within this framework, lactate elevation is expected whenever cytosolic NADH reoxidation and pyruvate oxidation become rate-limiting, because conversion of pyruvate to lactate by LDH preserves glycolytic throughput by restorative NAD⁺.

Consistent with this expectation, striatal lactate elevations have been uncovered in toxin-based PD models, encouraging lactate as an early metabolic signature of energetic stress instead of only a late consequence of neurodegeneration. High-field 1H-MRS in MPTP-treated mice demonstrated an increase in striatal lactate/creatine ratio that the authors interpreted as consistent with a mitochondrial energy crisis preceding dopaminergic neurotoxicity (Koga et al. 2006). In freely moving rats exposed to MPTP, in situ microdialysis revealed progressive increases in extracellular lactate and in lactate/pyruvate and lactate/glucose ratios alongside declining DA and pyruvate, again consistent with a shift toward a more decreased cytosolic redox state and limited oxidative metabolism (Bazzu et al. 2013). These studies support an important boundary condition for later pathophysiological claims: in PD-like states, lactate elevation is often downstream of mitochondrial dysfunction, but it may still feed back into mitochondrial and immune pathways.

#### Oxidative stress: hormesis versus overload under lactate homeostatic collapse

One reason lactate can act dually is that lactate oxidation interfaces directly with redox biology. A main study demonstrated that L-lactate (and pyruvate) can induce a mild ROS burst that activates buffering programs, including NRF2-linked antioxidant responses and unfolded protein response components, thereby increasing resistance to subsequent oxidative insults—a classic hormesis signature (Tauffenberger et al. 2019). This work provides strong evidence that lactate can be pro-resilience under specific dosing and cellular contexts.

However, PD is specifically the condition where a hormetic ROS increment is most likely to cross into overload. When respiratory chain function is compromised and antioxidant reserve is strained, even modest increases in electron transport activity can disproportionately elevate ROS generation. This is mechanistically salient because lactate is not only oxidized; emerging evidence suggests lactate itself can stimulate mitochondrial electron transport chain activity under conditions of extracellular lactate accumulation, increasing ATP synthesis and shifting substrate utilization (Cai et al. 2023). While this phenomenon may be adaptive in bioenergetically limited cells, it also raises a PD-relevant risk: if lactate-driven ETC stimulation occurs in a redox-fragile environment, it could amplify ROS and propagate mitochondrial damage instead of rescue it. An additional redox mechanism should be separated from the older view of lactate as only a mitochondrial fuel. Recent work reports that neuronal mitochondria can generate lactate as a venting mechanism that limits oxidative stress, and mitochondrial lactate can track circulating lactate with high fidelity. This suggests that a rise in neuronal or mitochondrial lactate may sometimes reflect redox pressure relief rather than increased respiratory fuel supply. For PD, this point is important: when dopaminergic loss and mitochondrial complex-I stress reduce redox buffering capacity, failure of lactate venting—or conversion of an initially protective vent into sustained lactate accumulation—could help explain why lactate is adaptive in acute exercise-like pulses but maladaptive during chronic mitochondrial-inflammatory collapse (Mao et al. 2024; Rauseo et al. 2026). The lactate literature hence supports a dose–state interaction model: lactate can engage hormetic protection in metabolically competent cells, but may amplify oxidative injury when mitochondrial reserve is limited—an interpretation aligned with the observation that lactate elevation in PD models often co-occurs with redox stress signatures (Koga et al. 2006).

#### Neuroinflammation: lactate as an immunometabolic regulator

The relationship between lactate and inflammation has moved beyond correlation: lactate can remodel inflammatory signaling via receptor-induced pathways, metabolic reprogramming, and covalent modifications.

In immune cells, lactate has been reported to inhibit pro-inflammatory outputs in several contexts. for instance, lactate can suppress macrophage pro-inflammatory responses through GPR81/HCAR1-induced signaling that engages AMPK/LATS and decreases NF-κB activity (Yang et al. 2020). In the CNS context, lactate treatment has been demonstrated to modulate microglial inflammatory responses and alleviate ischemic injury, with pathophysiological involvement of HIF-1α and inhibition of CCL7/NF-κB signaling (Zhang et al. 2025). These findings emphasize that lactate is not intrinsically pro-inflammatory and can, under several conditions, act as a brake on inflammatory cascades.

A parallel literature shows that glycolytic reprogramming toward lactate production can be permissive—or even necessary—for inflammasome activation. In macrophages, decreasing lactic acid fermentation by inhibiting LDH decreased caspase-1 activation and IL-1β maturation in response to multiple NLRP3 agonists, supporting the conclusion that lactic acid fermentation is required for robust NLRP3 inflammasome activation in those paradigms (Lin et al. 2021). Notably, very recent work extends this logic by proposing a direct pathophysiological coupling: endogenous lactate can promote NLRP3 inflammasome activation and pyroptosis through lactylation of NLRP3, with the modification promoting inflammasome complex formation (Liu et al. 2026). Even if this specific mechanism remains to be validated in brain-resident microglia in vivo, it strengthens the plausibility that lactate excess could feed forward into innate immune activation via both metabolic and post-translational routes.

#### PD-specific convergence: mitochondrial ROS–inflammasome loops and DA loss as a disinhibitor

PD provides a pathophysiological environment where lactate-linked metabolic shifts are abnormally likely to couple to neuroinflammation. First, mitochondrial dysfunction and oxidative stress are not only parallel features; they can proactively induce innate immune signaling. In a PD model context, MPTP/ATP treatment was reported to facilitate NLRP3 inflammasome assembly and activation in microglia via mitochondrial ROS production, and NLRP3 deficiency decreased microglial recruitment, IL-1β production, and caspase-1 activation in the substantia nigra (Lee et al. 2019). This establishes a direct mitochondrial ROS → inflammasome route in PD-relevant pathology, creating a mechanistic bridge via which lactate-driven redox shifts could modulate neuroinflammation.

Second, DA itself appears to restrain certain inflammatory programs. In primary human microglia, DA signaling has been demonstrated to inhibit canonical and α-syn–mediated activation of the NLRP3 inflammasome, suggesting that DA depletion could remove an endogenous repressive tone and thereby lower the threshold for inflammasome activation (Pike et al. 2022). This is especially pivotal to a lactate-centric hypothesis because DA depletion can promote network states (and glial activation states) that increase glycolytic reliance—conditions under which lactate generation and lactate-linked signaling become more likely to engage.

#### Lactylation: a pathophysiological route from lactate accumulation to persistent inflammatory state

The discovery of histone lysine lactylation offered a direct molecular mechanism whereby lactate availability can be incorporated into gene regulation. In the foundational study, lactate-derived histone lactylation was reported to stimulate gene transcription, building lactate as a precursor for an epigenetic modification with potential to induce durable state transitions (Zhang et al. 2019). While the best-established functional data come from macrophage systems, the conceptual implication for PD is substantial: chronic lactate elevation in a neuroinflammatory environment could bias microglia (and possibly astrocytes) toward transcriptional programs that sustain inflammation or alter phagocytic/proteostatic functions, even after the initial trigger wanes. At present, however, the current framework remains limited by a lack of cell-type-resolved in vivo lactylation maps in PD-relevant BG microenvironments, and by limited causal tests linking lactylation to striatal circuit outcomes.

#### Evidence for harm from lactate excess in PD models—and why it does not negate lactate’s adaptive roles

A core PD-specific dataset proposes that lactate excess can be pathogenic instead of compensatory: one study concluded that “excessive lactate in the brain” contributes to PD development and demonstrated that restoring lactate homeostasis through SIRT1-induced deacetylation and inhibition of PKM2 delayed parkinsonism, in contrast to elevating lactate could recapitulate PD-like features in their experimental system (Lian et al. 2024). This study is valuable specifically because it counters the tendency to assume that lactate rises are intrinsically protective. It also suggests a pathophysiological intersection between glycolytic control (PKM2), lactate accumulation, and neurodegenerative vulnerability.

Critically, these findings do not invalidate lactate’s protective actions under acute metabolic stress (including hormetic signaling), but instead refine a central theme for PD: Kinetics and compartmentalization also determine directionality. Transient lactate pulses associated with intact clearance and mitochondrial handling may be adaptive; maintained elevation induced by metabolic reprogramming and inflammatory activation may be maladaptive, promoting ROS–inflammasome feedback and lactylation-linked state fixation (Tauffenberger et al. 2019).

#### A disciplined working model and core gaps

**Fig. 2:**
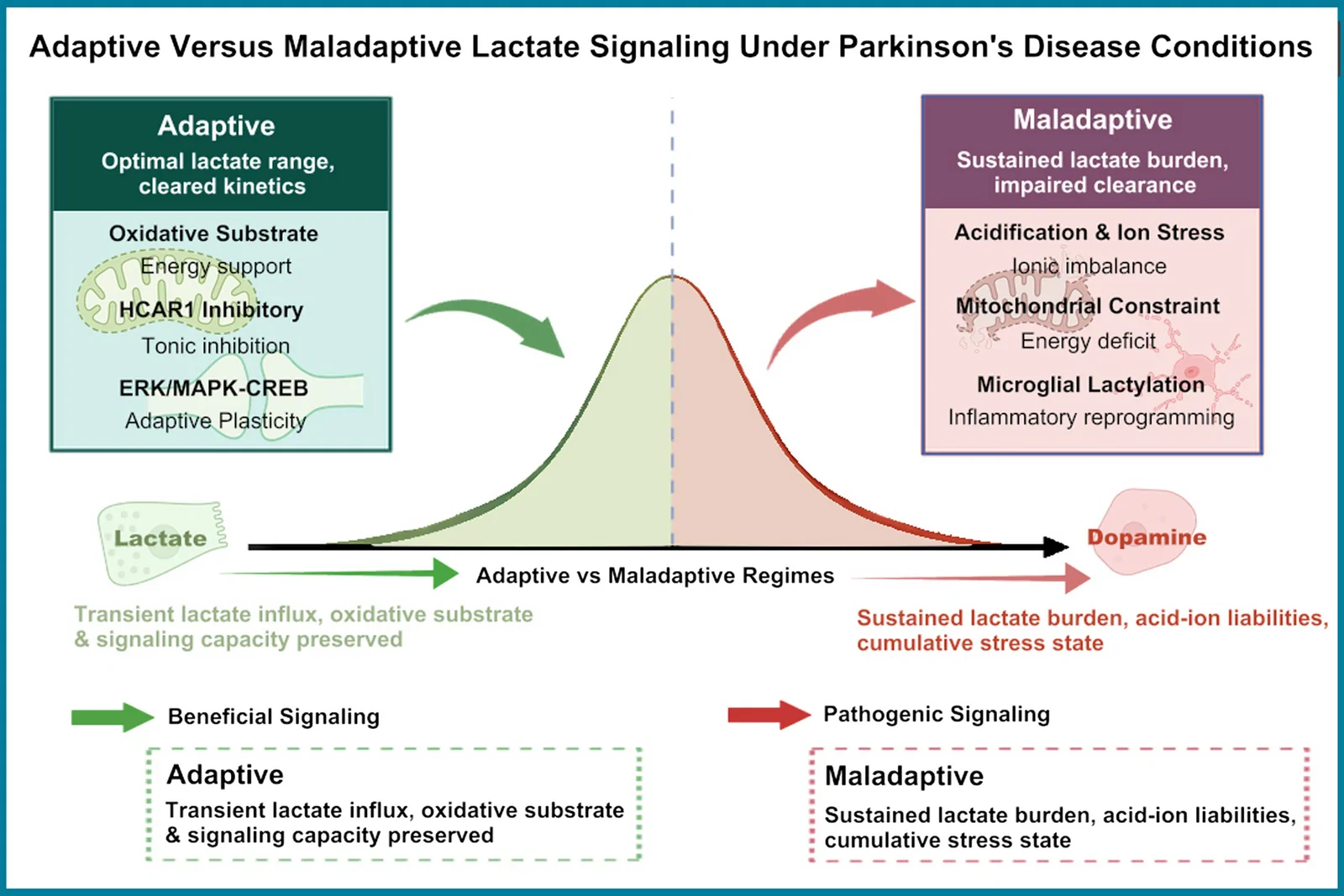
Adaptive and maladaptive regimes of lactate signaling in PD. Legend: Lactate actions in Parkinson’s disease (PD) are proposed to follow a state-dependent, two-regime framework rather than a uniformly beneficial or uniformly detrimental model. In an adaptive regime, modest or transient increases in lactate, coupled to preserved clearance and mitochondrial handling, may support oxidative metabolism, engage HCAR1-linked inhibitory signaling, and facilitate ERK/MAPK–CREB-associated plasticity programs. Under these conditions, lactate functions as both a substrate and a signal that can help stabilize neuronal activity and maintain synaptic responsiveness. By contrast, in a maladaptive regime, sustained lactate burden or lactate homeostatic collapse—typically coupled to mitochondrial constraint, impaired clearance, redox disequilibrium and inflammatory metabolic reprogramming—may shift lactate-associated signaling toward pathological outcomes. These include acid–ion imbalance, impaired transporter and membrane function, mitochondrial energetic failure, and microglial inflammatory state fixation, potentially reinforced by lactylation-linked transcriptional programs. In the striatum, such non-permissive conditions are predicted to reduce the threshold at which metabolic stress destabilizes dopaminergic transmission and receptor signaling, thereby promoting circuit dysfunction. The transition between adaptive and maladaptive regimes is therefore expected to be determined not simply by lactate abundance, but by its kinetics, duration, cellular source, compartmental distribution and the integrity of oxidative metabolism. Green arrows indicate putative adaptive pathways; red arrows indicate putative maladaptive pathways

The most defensible model compatible with current data is a two-regime framework (Fig. 2). Modest/transient lactate elevations support energetic flexibility and may engage hormetic ROS programs that increase resilience (Tauffenberger et al. 2019). In addition, maintained lactate homeostatic collapse (often implying mitochondrial constraint) amplifies oxidative stress and primes or sustains neuroinflammation via lactic fermentation dependence of inflammasome signaling and lactylation-enabled inflammatory state programs (Lin et al. 2021).

Several decisive gaps remain for a PD-striatum-specific claim: (1) What are the extracellular lactate and lactate/pyruvate dynamics in striatum across PD stages and during stressors (exercise, L-DOPA, inflammation)? Current MRS and microdialysis data support elevations, but are not yet cell- and synapse-resolved (Koga et al. 2006). (2) Do interventions that normalize lactate (or lactate/pyruvate ratio) reduce mitochondrial ROS and inflammasome activation in BG, and do they improve D2-MSNs network state and motor vigor (Lian et al. 2024)? (3) In PD tissue, what fraction of lactate’s impact on inflammation is induced by HCAR1 signaling versus metabolic reprogramming versus lactylation? The literature supports each route in principle, but the dominant pathway in striatal microenvironments is not established (Yang et al. 2020).

Overall, lactate homeostatic collapse sits at a plausible pathophysiological intersection of mitochondrial constraint, oxidative stress and innate immune activation in PD. The weight of evidence now supports treating lactate not only as a substrate and signal, but also as a state-setting metabolite whose chronic elevation can plausibly engage in self-reinforcing ROS–inflammasome loops—yet the current framework still needs striatum- and cell-type-resolved causal experiments to recognize when lactate is adaptive compensation versus pathological driver.

### Disruption of DA transmission and receptor function

Dopamine (DA) transmission in the striatum is abnormally vulnerable to metabolic perturbation because both release (vesicle cycling, Ca^2^⁺ handling) and clearance (dopamine transporter, DAT) are limited by ionic gradients and ATP availability, and because extracellular DA itself can amplify oxidative and inflammatory cascades when dysregulated. A central challenge in assigning lactate-specific causality is that numerous relevant findings arise in paradigms where lactate accumulation is tightly associated with acidification, redox shift (lactate/pyruvate), or bioenergetic failure. Accordingly, the most rigorous interpretation is that lactate homeostatic collapse perturbs DA transmission via three partially separable interfaces: (i) pH/ion-gradient dependence of DAT and vesicular processes, (ii) second-messenger crosstalk that reshapes DA receptor signaling efficacy, and (iii) lactate-linked epigenetic programs (including lactylation) that can bias transcriptional states affecting dopaminergic synapses and treatment response.

#### Homeostatic collapse of DA release and clearance

Early in vivo microdialysis work demonstrated that experimentally induced striatal lactacidosis (local lactic acid perfusion to reach ischemia-relevant pH) causes a biphasic increase in extracellular DA, and offered pharmacological evidence for glutamatergic and oxidative mechanisms contributing to this response (Remblier et al. 1999). A related study explicitly investigated whether striatal DA elevation during “lactic acidosis” is related to acidification or lactate production per se, stressing the field’s long-standing recognition that DA homeostatic collapse in these paradigms is fundamentally an acid–metabolic composite instead of a lactate-only effect (Remblier et al. 1998). While these experiments are ischemia-motivated, they build a proof-of-principle: when extracellular pH and lactate rise together in striatal tissue, DA homeostasis can fail rapidly, generating DA overflow capable of perturbing striatal computation and viability.

DAT-induced uptake is a Na⁺/Cl⁻-dependent process whose capacity depends on maintained ion gradients and membrane potential (Bu et al. 2021). Multiple main studies in striatal preparations report that severe acidosis decreases high-affinity DA uptake, including experiments where acidosis was induced either by lactic acid or by non-lactate acidification, implicating proton load (and associated oxidative damage) as a main driver instead of lactate as a ligand (Barrier et al. 2003). In rat striatal synaptosomes, “lactic acidosis” progressively weakened DA uptake and was linked mechanistically to dysfunction of Na⁺/K⁺-ATPase, consistent with a cascade where acid/oxidative stress compromises ion-pump support for transporter function (Barrier et al. 2004). This matters for PD because even small reductions in effective DAT function can transform DA signaling from specifically timed phasic transients to prolonged, spatially diffuse extracellular DA—degrading signal fidelity and shifting receptor engagement dynamics.

Beyond neuron-centric mechanisms, evidence indicates that astrocytes can be core contributors to ischemia-induced DA release in striatum, placing glial metabolism (including lactate handling) upstream of DA overflow during metabolic crisis (Oliva et al. 2013). Although ischemia is not PD, the pathophysiological inference is relevant: condition that push glia toward glycolytic dominance and lactate export may coincide with modified extracellular DA dynamics when clearance is compromised.

The strongest release/clearance interference data come from acute ischemia-like or experimentally acidified preparations instead of chronic PD (Remblier et al. 1999). For PD, the most defensible conclusion is conditional: if PD-related metabolic vulnerability decreases the threshold for local acidification or ion-gradient compromise, then lactate–acid co-elevation could disproportionately destabilize DA signaling in striatum by simultaneously increasing release/overflow and impairing uptake.

#### Effects on DA receptor function and expression

Because D1- and D2-family receptors decode extracellular DA dynamics on different timescales, any metabolic impairment that slows uptake is expected to remodel receptor engagement, desensitization pressure, and downstream transcriptional programs. This is especially salient in PD, where striatal DA loss forces greater reliance on pharmacologically induced DA surges, making uptake kinetics a determinant of both therapeutic benefit and side effects. A pathophysiological layer often overlooked in metabolic discussions is that D2 receptors can modulate DAT function, meaning reciprocal coupling between DA receptor signaling and DA clearance capacity (Bolan et al. 2007). In such a coupled system, metabolic condition that perturb uptake (via ion gradients) can indirectly alter receptor state, while modified receptor signaling can further modify uptake—creating potential feedback loops.

Although direct evidence that extracellular pH shifts change D1/D2 ligand binding in vivo is limited, main work shows that D2 receptor activation can stimulate Na⁺/H⁺ exchange and produce extracellular acidification, displaying that DA receptor signaling can itself influence local pH microenvironments (Neve et al. 1992). This observation supports a comprehensive systems view: DA signaling and pH regulation are not independent layers; they can be coupled, especially in densely innervated striatal microdomains where transporter and exchanger activities are high.

Direct, receptor-proximal evidence that lactate itself (independent of pH/redox changes) modifies DA receptor expression in striatal neurons remains sparse. Where lactate is most plausibly implicated today is indirectly: by modifying DA clearance (thereby changing receptor stimulation patterns) and by modulating the intracellular signaling milieu (cAMP/PKA and ERK pathways) that determines receptor sensitivity and downstream transcription (Bu et al. 2021).

#### Epigenetic interference via lactylation-dependent transcriptional changes

The discovery that lactate can cause histone lysine lactylation established an explicit molecular bridge from metabolic state to gene regulation (Merkuri et al. 2024). For PD, the most immediate implication is not that lactylation must directly modulate dopaminergic genes, but that chronic lactate elevation could buffer glial and immune transcriptional programs that secondarily remodel dopaminergic synapses and receptor responsiveness.

A study demonstrated that inhibiting glycolysis delayed microgliosis, neuroinflammation and dopaminergic neuron damage in PD mice by decreasing lactate accumulation, and drives histone lactylation as part of the pathway connecting glycolytic lactate to microglial activation (Qin et al. 2025). Meanwhile, a study argued that “excessive lactate in the brain” contributes to PD development and that restoring lactate homeostasis through SIRT1–PKM2 modulation alleviates parkinsonism in mice (Lian et al. 2024). These studies support a rigorous possibility: lactate homeostatic collapse can move from being a marker of mitochondrial constraint to a state-setting driver of neuroinflammatory milieu, which is known to influence DA terminal function, synaptic gain and pharmacological responsiveness.

Current lactylation evidence in PD is strongest for microglial programs and inflammatory outcomes instead of direct regulation of DAT/DRD1/DRD2 transcription in striatal neurons (Qin et al. 2025). A pathophysiological claim for “lactylation-driven DA receptor remodeling” will need cell-type-resolved lactylation maps in striatal SPNs and dopaminergic terminals, plus causal perturbations of lactylation writers/erasers that illustrate specific effects on DA signaling metrics.

#### Implications for L-DOPA–induced dyskinesia and treatment resistance

Levodopa-induced dyskinesia (LID) is induced by non-physiological, pulsatile dopaminergic stimulation in a denervated striatum and is tightly linked to pathological intracellular signaling in SPNs—notably exorbitant D1 receptor–dependent ERK1/2 activation and downstream transcriptional remodeling (Fieblinger et al. 2014). In this setting, any metabolic perturbation that increases DA peaks (e.g., weakened DAT clearance) or that amplifies ERK pathway gain could, in principle, worsen dyskinesia risk, while interventions that buffer DA kinetics or dampen ERK overactivation can reduce LID expression.

Two converging lines of evidence motivate (but do not yet prove) lactate involvement. Acid–lactacidosis paradigms can cause large extracellular DA elevations in striatum and engage oxidative/glutamatergic mechanisms (Remblier et al. 1999). In addition, in the DA-denervated striatum, D1R-induced ERK activation is a core molecular driver of LID (Fieblinger et al. 2014).

The critical gap is direct causality: no definitive study yet shows that manipulating lactate kinetics (or lactate-linked pH shifts) in vivo modifies LID severity through a specific molecular pathway. Hence, lactate should be framed as a candidate modifier of LID—most plausibly via its ability to remodel DA clearance and redox/ERK tone—instead of as an established driver.

Clinical treatment resistance is multifactorial (progressive denervation, receptor adaptations, network remodeling). The emerging lactate–lactylation–microglia literature supports an additional axis: chronic lactate homeostatic collapse could sustain neuroinflammation and thereby modify synaptic responsiveness and dopaminergic signaling efficacy (Qin et al. 2025). Again, the core need is pathophysiological resolution in BG: whether normalizing lactate (or lactate/pyruvate) improves DA signaling fidelity and decreases pathological plasticity under chronic dopaminergic therapy.

A rigorous test set for this section would include: (i) in vivo striatal lactate and pH measurements during L-DOPA dosing (ideally with genetically encoded sensors), (ii) targeted perturbation of lactate transport (MCT) or lactate homeostasis (e.g., LDH/PKM2 axis) during chronic L-DOPA to assess LID development, and (iii) linkage of behavioral outcomes to DAT kinetics and ERK-dependent transcriptional signatures in identified SPN populations. The existing evidence base supports plausibility and priority, but not closure, on these mechanisms (Barrier et al. 2004).

### Potential links between lactate homeostatic collapse and α-Syn aggregation

A link between lactate homeostatic collapse and α-Syn aggregation is plausible, but current evidence does not support a direct causal claim. The strongest PD-relevant evidence places lactate within microglial metabolic reprogramming and inflammatory state maintenance, rather than showing that lactate directly modifies α-Syn aggregation kinetics. Therefore, this section treats lactate as a potential amplifier of aggregation-prone microenvironments—through inflammation, proteostasis stress, oxidative injury, and possible lactylation-linked cell-state changes—rather than as an established upstream trigger of α-Syn pathology (Qin et al. 2025).

#### Lactate-associated microglial activation and inflammatory amplification

Microglia provide the clearest interface between lactate dysregulation and α-Syn pathology. α-Syn exposure can reprogram microglial metabolism toward glycolysis through PKM2, increasing lactate generation and promoting activated or migratory phenotypes (Qiao et al. 2019). Conversely, PD-model evidence suggests that glycolysis-derived lactate and microglial histone lactylation, including H3K9la-linked transcriptional programs, can reinforce inflammatory activation; glycolysis inhibition reduces lactate accumulation, microgliosis, and neuroinflammatory injury in these models (Qin et al. 2025). Together, these findings support a bidirectional model in which α-Syn can increase microglial lactate production, while lactate/lactylation may stabilize inflammatory states that indirectly favor oxidative stress, impaired clearance, and tissue conditions permissive for α-Syn propagation (Qiao et al. 2019).

#### Autophagy impairment and decreased proteostasis capacity

Proteostasis failure is a proximal route by which inflammatory and metabolic states could influence α-Syn burden. Microglial autophagy is relevant because microglia internalize and process extracellular α-Syn species. α-Syn fibrils can suppress ULK1-dependent microglial autophagy in PD-relevant models, while inflammatory stimulation can impair phagosome maturation, chaperone-mediated autophagy, mitochondrial function, and α-Syn PFF handling (Pei et al. 2024; Niskanen et al. 2025). Microglia can also redistribute fibrillar α-Syn through tunnelling nanotube networks, but this cooperative clearance mechanism is weakened by PD-linked LRRK2 G2019S, illustrating how genetic risk can reduce microglial proteostatic reserve (Scheiblich et al. 2021).

The role of lactate in this proteostasis axis remains unresolved. Non-neural evidence links lactate metabolism to lysosomal acidification and autophagy flux through LDHB-dependent mechanisms (Brisson et al. 2016). Lactylation can also affect autophagy regulators, including TFEB, and may increase lysosomal/autophagic activity in some settings (Huang et al. 2024). In other disease contexts, lactylation has been associated with mTORC1-linked suppression of autophagy, showing that the direction of effect is target- and context-dependent (Fan et al. 2025). Therefore, lactate/lactylation should not be presented as uniformly impairing α-Syn clearance. The defensible conclusion is that lactate homeostatic collapse may modulate autophagy–lysosome capacity, but the sign and relevance of this modulation in PD striatal microglia remain open questions (Qin et al. 2025).

#### Oxidative stress–driven protein misfolding and aggregation propensity

Oxidative stress provides a more established bridge to α-Syn aggregation. In vivo and molecular evidence indicates that oxidative injury can promote α-Syn aggregation and aggregation-related toxicity (Scudamore and Ciossek 2018). Lipid peroxidation products such as 4-hydroxy-2-nonenal can covalently modify α-Syn and alter its aggregation behavior, while oxidized membrane environments can facilitate α-Syn aggregate–membrane toxicity and ferroptosis-like injury in human stem-cell-derived models (Qin et al. 2007; Angelova et al. 2020). Lactate homeostatic collapse may intersect with this axis indirectly: elevated lactate can mark mitochondrial constraint, altered lactate/pyruvate redox state, or inflammatory glycolytic reprogramming. Under such conditions, lactate is not required to initiate α-Syn aggregation to be relevant; it may reflect or amplify the redox and inflammatory environment in which misfolding and impaired clearance become more likely (Qin et al. 2025).

#### Direct protein modification via lactylation: plausibility and hypotheses

Direct lactylation of α-Syn remains speculative. Current lactylation biology supports widespread histone and non-histone lysine lactylation, and methodological advances have clarified that glycolysis-induced L-lysine lactylation should be distinguished from related isomers that may complicate detection and interpretation (Zhang et al. 2025). Work on lactyl-CoA generation and acyltransferase-linked mechanisms also strengthens the plausibility that lactylation can occur through regulated biochemical routes rather than only through nonspecific chemical reactivity (Zhu et al. 2025). However, there is still no strong evidence that α-Syn itself is lactylated in vivo in PD brain, nor that α-Syn lactylation alters oligomerization, fibril formation, seeding competence, or clearance. Given the lysine-rich nature of α-Syn and its sensitivity to charge-altering post-translational modifications, this remains a testable hypothesis, but not an established mechanism (Zhang et al. 2025).

#### Feed-forward loops between inflammation, metabolism, and aggregation

The most reasonable integrative model is a feed-forward loop in which lactate reflects and potentially stabilizes an inflammatory metabolic state. α-Syn can promote microglial glycolysis and PKM2-dependent lactate production (Qiao et al. 2019) Lactate accumulation and lactylation-linked transcription may then reinforce pro-inflammatory microglial programs in PD models (Qin et al. 2025). Inflammatory activation can impair α-Syn handling by disrupting phagosome maturation, chaperone-mediated autophagy, and mitochondrial function (Niskanen et al. 2025). Oxidative stress can further increase aggregation propensity and membrane-associated toxicity (Scudamore and Ciossek 2018). Aggregated α-Syn can also feed back onto microglia, including through inflammasome-related pathways, while cooperative microglial clearance mechanisms may be weakened by PD-linked genetic risk such as LRRK2 G2019S (Scheiblich et al. 2021; Scheiblich et al. 2021). In this loop, lactate is best positioned as a state marker and possible amplifier of a pathological microenvironment, not as the sole or primary cause of α-Syn aggregation.

#### Summary and core evidence gaps

Current evidence supports four cautious conclusions. First, α-Syn can induce glycolytic reprogramming and lactate generation in microglia (Qiao et al. 2019). Second, PD-model evidence links glycolysis-derived lactate and microglial histone lactylation to inflammatory activation and dopaminergic injury (Qin et al. 2025). Third, microglial autophagy and clearance pathways influence α-Syn burden and can be impaired by α-Syn or inflammatory states (Pei et al. 2024). Fourth, oxidative stress can promote α-Syn aggregation and modify aggregation trajectories through lipid and protein damage (Scudamore and Ciossek 2018).

Several evidence gaps remain decisive. It is still unknown whether selectively normalizing microglial lactate or lactylation reduces α-Syn aggregation and spread independently of broad anti-inflammatory effects (Qin et al. 2025). The concentration, timing, cellular source, and compartmental distribution of lactate during α-Syn propagation in striatal microenvironments remain poorly defined. The direction of lactate/lactylation effects on autophagy–lysosome pathways may be stage- and target-dependent, including through regulators such as TFEB (Huang et al. 2024). Finally, direct lactylation of α-Syn or core α-Syn proteostasis machinery has not been established in vivo, and any proposed role in seeding, fibril polymorph selection, or clearance susceptibility requires site-resolved biochemical validation (Zhang et al. 2025).

##  D2-MSNs as a high-priority test bed for lactate-sensitive striatal mechanisms

D2-MSNs are proposed here as a high-priority experimental test bed, not as a demonstrated site of lactate-mediated protection. The rationale is anatomical and pathophysiological: indirect-pathway D2-MSNs sit at a major bottleneck for hypokinetic basal ganglia output and are sensitive to dopamine loss, glutamatergic drive, cAMP/PKA signaling, ERK-dependent plasticity and metabolic stress. However, most lactate mechanisms discussed in this Review have been established in hippocampal, cortical, vascular, glial, peripheral or mixed-cell systems. Therefore, all claims that lactate directly modifies D2-MSN excitability, ERK kinetics, synaptic plasticity or beta-linked recruitment should be treated as Tier 5 hypotheses until tested in genetically identified D2-MSNs in PD-relevant models.

###  Candidate lactate-sensitive mechanisms to be tested in D2-MSNs

A mechanistically useful way to frame candidate lactate effects on indirect-pathway D2 medium spiny neurons (D2-MSNs; iSPNs) is to separate exposure, capacity, and consequence. First, D2-MSNs must experience extracellular lactate transients in the relevant spatiotemporal window. Second, they must express the molecular machinery to import and/or sense lactate. Third, lactate must measurably shift D2-MSNs input–output properties, synaptic integration, or plasticity rules. At present, the first two steps are progressively well supported, in contrast to the third—cell-type-resolved illustrations in identified D2-MSNs in striatum—remains the major evidence gap that limits causal claims.

#### D2-MSNs are exposed to swift lactate transients in striatum

Real-time measurements show that striatal extracellular lactate fluctuates on synaptic timescales but do not identify D2-MSN-specific exposure or utilization. In rat striatum, stimulation of midbrain projections caused rapid glucose and lactate rises followed, at high intensity, by below-baseline depletion, consistent with demand-driven supply–use dynamic (Forderhase et al. 2020). These results establish that striatal lactate is not only a slow metabolic drift variable, but a fast, activity-coupled extracellular signal/substrate. Whether such transients are preferentially sensed or used by D2-MSNs remains unresolved and requires cell-type-resolved lactate imaging or perturbation.

#### Import capacity: MCT2 kinetics support “threshold-like” lactate uptake in neurons

For lactate to act as a swift substrate, neuronal uptake must be sufficiently fast and concentration-sensitive. A structural and functional analysis of human MCT2 (SLC16A7)—a high-affinity neuronal monocarboxylate transporter—revealed a cooperative transport mechanism and a steep dependence of transport activity on substrate concentration, consistent with a “switch-like” uptake response over a narrow concentration range (Zhang et al. 2020). Although not striatum-specific, this property is conceptually important for D2-MSN physiology: it predicts that small increments in extracellular lactate—such as those uncovered during striatal activation—could produce disproportionately large changes in neuronal lactate influx once local concentrations cross a kinetic threshold.

Meanwhile, advances in genetically encoded lactate sensors have substantially tightened the measurement problem. Ultrasensitive in vivo-compatible sensors reveal that lactate dynamics can be highly compartmentalized and rapid at the cellular level, encouraging the feasibility of lactate microdomains that could be experienced differentially across neuronal subtypes (Li et al. 2023). The core limitation for D2-MSNs biology is that analogous cell-type-identified lactate imaging in striatal microcircuits remains comparatively sparse, leaving uncertainty about whether D2-MSNs preferentially import lactate versus rely mainly on local glucose metabolism under specific behavioral states.

#### Candidate excitability control: lactate can engage Gi-coupled inhibitory signaling, but striatal D2-MSNs evidence remains indirect

D2-MSN excitability is strongly shaped by Gi/o-coupled signaling (notably via D2 receptors), which constrains cAMP/PKA tone and downstream channel and synapse regulation. A mechanistically attractive hypothesis is that lactate can recruit an auxiliary Gi brake via the lactate receptor HCAR1 (GPR81), thereby damping excitability and buffering network state when extracellular lactate rises.

Primary evidence supports the inhibitory competence of HCAR1 signaling at cellular and network levels: in mouse primary cortical neurons, HCAR1 activation decreased neuronal network activity and decreased excitatory synaptic transmission via Giα- and Gβγ-dependent pathways (de Castro Abrantes et al. 2019). These data provide a proof-of-principle that lactate can directly reduce neuronal excitability through a specific receptor pathway. Nevertheless, translating this to striatal D2-MSNs needs two missing demonstrations: (i) robust receptor expression/localization in the relevant striatal compartments and (ii) direct electrophysiological evidence that lactate (or selective HCAR1 agonism) shifts the intrinsic excitability of genetically identified D2-MSNs in acute slices or in vivo.

A further constraint is physiological: HCAR1 activation typically needs extracellular lactate in the millimolar range. Consequently, if HCAR1 contributes to D2-MSNs control, it is most likely to operate as a state-dependent brake engaged during high-demand epochs (for instance, intense network recruitment, stress, or systemic lactate surges), instead of as a tonic regulator under basal conditions (de Castro Abrantes et al. 2019).

#### Synaptic transmission: lactate can dampen neurotransmission, but region- and state-dependence are critical

Even when lactate acts as a fuel, it can simultaneously alter synaptic transmission. In hippocampal slice preparations, lactate was demonstrated to attenuate neurotransmission at both glutamatergic and GABAergic synapses and to alter network rhythms, with the authors stressing that lactate-only fueling can be less effective and possibly detrimental during high-energy-demand oscillations—interpreted as a consequence of omitting obligatory fast glycolysis at synapses (Hollnagel et al. 2020).

This finding is not striatum-specific, but it is mechanistically pivotal to D2-MSNs because indirect-pathway computation depends on the fine balance of cortical excitation, feedforward inhibition (e.g., from fast-spiking interneurons), and intrinsic dendritic integration. If lactate dampens presynaptic release probability or modifies postsynaptic responsiveness in striatal synapses, it could shift the probability that D2-MSNs ensembles are engaged into suppressive network states. The core uncertainty is direction: a modest depression of excitatory drive could be buffering (reducing hyperexcitability), in contrast to comprehensive inhibition of both excitation and inhibition could degrade the selectivity and timing precision required for action selection (Hollnagel et al. 2020). These alternative outcomes underscore the need for striatal synapse-resolved tests (corticostriatal vs thalamostriatal; synapse onto D2-MSNs vs D1-MSNs; excitatory vs inhibitory terminals) instead of relying on extrapolation from hippocampal rhythms.

#### Plasticity: lactate could bias D2-MSNs learning rules, but decisive experiments are pending

D2-MSNs display distinctive corticostriatal plasticity machinery, making them plausible targets for lactate-dependent plasticity modulation. In a landmark study, selectively increasing activity in indirect-pathway MSNs have larger NMDAR currents and selectively express endocannabinoid-induced LTD (eCB-LTD) that needs D2 receptor activation (Kreitzer and Malenka 2007). DA depletion hence destabilizes a D2-dependent plasticity gate, contributing to pathological learning rules in PD. Separately, One study using dopamine receptor transgenic mice showed that dopaminergic control of striatal synaptic plasticity is not simply unidirectional by cell type, stressing that plasticity outcomes depend on the interaction of receptor state, stimulation pattern, and intracellular signaling context (Shen et al. 2008).

Where can lactate enter? Two routes are most defensible: lactate-driven redox–NMDAR–ERK gain could shift plasticity thresholds, and larger NMDAR currents in indirect-pathway MSNs may increase sensitivity to this shift; however, this remains inferential until tested directly in identified D2-MSNs (Kreitzer and Malenka 2007). Second, lactate may exert metabolic enabling of energy-intensive consolidation processes: lactate availability and monocarboxylate uptake can be required for activity-dependent protein synthesis in other brain circuits (Sect. " Synapse-specific modulation and energy-demanding cellular processes"), raising the possibility that lactate shapes D2-MSN plasticity by enabling translation-dependent phases of LTD/LTP consolidation, with the core unknown being whether corticostriatal plasticity in D2-MSNs is similarly lactate-sensitive at the level of translation and local dendritic metabolic support, especially under DA-depleted condition where energetic reserve may be reduced.

Existing data justify a narrower hypothesis: lactate transients may alter D2-MSN plasticity through synaptic drive, HCAR1/D2R-linked cAMP/PKA tone, and NMDAR/ERK coupling (de Castro Abrantes et al. 2019). This should be tested with controlled lactate application in genetically identified D2-MSNs, matched pH/osmolarity, MCT and HCAR1 perturbations (de Castro Abrantes et al. 2019), in vivo lactate sensing with D2-MSN calcium/voltage imaging during indirect-pathway recruitment (Forderhase et al. 2020), and plasticity assays for eCB-LTD or ERK-dependent signatures (Kreitzer and Malenka 2007).

Overall, the current literature supports a strong plausibility scaffold—rapid striatal lactate transients, threshold-like neuronal uptake capacity, and a validated inhibitory lactate receptor pathway—but still lacks decisive, D2-MSN-specific illustrations that lactate directly re-parameterizes excitability, synaptic gain, and corticostriatal plasticity rules in the striatum.

### Candidate routes linking lactate-sensitive signaling to D2-MSN ERK/MAPK programs

#### Hypothesized routes by which lactate could influence ERK/MAPK signaling in D2-MSNs

ERK1/2 (MAPK) signaling in striatal medium spiny neurons (MSNs) is a canonical integrator of glutamatergic input, neuromodulator tone and activity history, and it is especially pivotal to PD because it integrates transient synaptic events to longer-lived changes in gene expression and synaptic efficacy. In D2-MSNs (iSPNs), ERK activation is demonstrably reachable in vivo: D2 receptor blockade (e.g., haloperidol) selectively induces ERK phosphorylation and immediate-early gene expression in D2R-expressing striatal output neurons, providing proof that the ERK module is not limited to D1-MSNs (Bertran-Gonzalez et al. 2008). Nevertheless, a core limitation for the present review is that direct, cell-type-resolved evidence that lactate itself activates ERK in identified D2-MSNs in striatum is still sparse. Accordingly, this section lays out the most plausible pathophysiological routes—grounded in main data from lactate signaling and striatal ERK biology—and specifies what would constitute decisive evidence in D2-MSNs.

The most mechanistically limited pathway whereby lactate engages ERK in neurons is metabolic-redox coupling upstream of NMDARs. Yang and colleagues demonstrated that L-lactate induces plasticity-related immediate-early genes through an NMDAR-dependent mechanism that needs ERK1/2 signaling, and linked this effect to intracellular redox changes produced during lactate-to-pyruvate conversion (Yang et al. 2014). Subsequent pathophysiological work nuanced the receptor-level step: L-lactate can exert bidirectional control over NR2B-containing NMDAR signaling depending on glutamate regime, and the potentiating node is associated with NADH formation and NR2B modulation (Jourdain et al. 2018). For D2-MSNs, this route is appealing because the NMDAR → ERK coupling machinery is already well defined. RasGRF1 binds the NR2B subunit and is required for efficient NMDAR-dependent ERK activation in neurons, offering a direct molecular bridge from NR2B gating to Ras–ERK engagement (Krapivinsky et al. 2003). In a striatal context, this predicts a concentration- and state-dependent effect: lactate should most effectively amplify ERK in D2-MSNs when (i) extracellular lactate crosses the uptake/oxidation threshold, (ii) synaptic glutamate is in a permissive (subthreshold-to-moderate) regime instead of commonly excitotoxic drive, and (iii) NR2B/RasGRF coupling is intact (Jourdain et al. 2018). The core experimental implication is straightforward: in genetically identified D2-MSNs, any lactate-evoked ERK phosphorylation should be blocked by NMDAR antagonism and decreased by avoiding lactate uptake/processing, instead of persisting under condition that eliminate Ca^2^⁺ entry via NMDARs (Yang et al. 2014). This mechanism is best supported in cortical/hippocampal preparations and does not yet constitute direct evidence in striatum. In addition, lactate manipulations can co-vary with pH and osmolarity; therefore, a D2-MSNs claim needs matched-pH controls and explicit separation of lactate transport/metabolism from extracellular acid effects (Jourdain et al. 2018).

A second pathophysiological entry point is receptor-induced signaling via HCAR1 (GPR81). In primary cortical neurons, HCAR1 activation down-modulates neuronal network activity via signaling that needs Giα and Gβγ subunits, and it can reduce excitatory transmission and intrinsic excitability—establishing that lactate can trigger specific GPCR signaling in neurons (de Castro Abrantes et al. 2019). Meanwhile, lactate–HCAR1 signaling in brain tissue has been related to downstream kinase activation (including ERK1/2 and Akt phosphorylation) in contexts related to vascular adaptation (Morland et al. 2017). If HCAR1 contributes to ERK regulation in D2-MSNs, two non-exclusive outcomes are plausible. First, Gβγ-dependent pathways can recruit kinase cascades upstream of ERK, allowing ERK phosphorylation despite overall Gi-induced inhibition of cAMP (de Castro Abrantes et al. 2019). Second, β-arrestin scaffolding could bias the compartmental fate of activated ERK: β-arrestins can enhance GPCR-induced ERK activation, but can also retain phospho-ERK in the cytosol, thereby limiting ERK-dependent transcription while preserving local cytosolic signaling (Tohgo et al. 2002). This distinction is possibly decisive for PD: in D2-MSNs, cytosolic ERK could modulate local excitability and synaptic machinery, in contrast to nuclear ERK would be expected to engage transcriptional programs that could either support adaptive plasticity or promote pathological state changes depending on context. The central uncertainty is anatomical and cell-type specific: robust, functional HCAR1 expression in D2-MSNs compartments in vivo has not been established at the same level of confidence as in cortex/hippocampus. Hence, HCAR1 → ERK should be framed as a high-priority hypothesis instead of a settled mechanism in D2-MSNs, and it demands striatum- and cell-type-specific perturbation (conditional Hcar1 deletion in iSPNs) to move beyond plausibility (de Castro Abrantes et al. 2019).

D2-MSNs are embedded in a distinctive second-messenger environment specific by antagonistic convergence of D2 receptors (Gi/o) and adenosine A2A receptors (Gs/olf). In vivo, locomotion drives iSPN PKA signaling through acute adenosine accumulation, and the balance of DA and adenosine receptor engagement sculpts iSPN signaling state (Ma et al. 2022). At the molecular level, A2A receptor stimulation in vivo modulates both DARPP-32 phosphorylation and ERK1/2 phosphorylation, confirming that the A2A → cAMP axis can access ERK in striatal tissue (Botsakis et al. 2010). In addition, D2R blockade triggers ERK activation selectively in D2 neurons, consistent with a model where removal of D2-induced inhibition unmasks A2A/cAMP-dependent signaling routes that can converge on ERK (Bertran-Gonzalez et al. 2008). Within this architecture, lactate could influence ERK in D2-MSNs even without being a main ERK trigger, by shifting the gain of upstream modulators. A Gi-coupled lactate receptor (HCAR1) would be expected to reduce cAMP tone and possibly oppose A2A-driven ERK engagement, in contrast to MCT-dependent lactate uptake could amplify NMDAR-driven ERK engagement via redox–NMDAR coupling (Route 1) (de Castro Abrantes et al. 2019). These opposing tendencies imply a core prediction: lactate may not simply “activate ERK” in D2-MSNs; it may reparameterize which upstream channel dominates ERK (cAMP/A2A versus NMDAR/redox), with different downstream consequences for nuclear transcription versus local synaptic effects.

ERK is redox-sensitive, and lactate can induce a mild ROS burst that activates buffering programs (hormesis) in neuronal models (Tauffenberger et al. 2019). In metabolically competent cells, such ROS signaling could plausibly reinforce ERK activation downstream of synaptic Ca^2^⁺ signals, effectively functioning as a gain control. In PD-like metabolic constraint, however, the same lactate-linked ROS increment could become maladaptive, amplifying oxidative stress and thereby modifying ERK dynamics (duration, localization) in ways that favour pathological plasticity (Tauffenberger et al. 2019). Whether this effect in D2-MSNs remains untested, but it motivates including redox-state controls (e.g., NADH/NAD⁺ metrics; antioxidant manipulations) in any attempt to attribute ERK activation to lactate.

To establish a D2-MSN-specific mechanism, studies should quantify ERK in genetically identified D2-MSNs, separate lactate effects from pH/osmolarity and co-varying neuromodulators, and test NMDAR and HCAR1 necessity under matched conditions (Jourdain et al. 2018). They should also vary synaptic drive because lactate effects on NR2B-containing NMDARs are glutamate-regime dependent (Jourdain et al. 2018). Taken together, the strongest current pathophysiological scaffold for lactate → ERK signaling in D2-MSNs is MCT-dependent redox modulation that potentiates NR2B–NMDAR signaling with RasGRF-induced coupling to ERK, in contrast to HCAR1-induced signaling remains a plausible parallel route that may preferentially gate ERK localization and downstream output instead of simply amplifying nuclear transcription (Yang et al. 2014).

#### Synergy between lactate signaling and NMDAR-dependent plasticity

A core reason lactate is mechanistically attractive in indirect-pathway biology is that it can, in principle, amplify specifically the receptor-dependent Ca^2^⁺ signals that gate long-term synaptic change. In numerous neuron types, NMDAR activation is a coincidence detector for presynaptic glutamate release and postsynaptic depolarization; downstream, it drives ERK/MAPK–CREB programs that consolidate plasticity. In striatum, NMDAR-dependent corticostriatal plasticity is a major substrate for motor learning and habit formation, and it remains operationally relevant even when overall MSN firing is sparse (Zhou et al. 2018).

The most direct evidence for lactate–NMDAR synergy comes from primary neuronal systems demonstrating that L-lactate potentiates NMDAR-induced currents and Ca^2^⁺ signaling and, via this, drives ERK1/2 activation and immediate-early gene induction. In the original pathophysiological study, lactate stimulated expression of plasticity-related genes (including Arc, c-Fos and Zif268/Egr1) through a pathway requiring NMDAR activity and downstream ERK1/2 signaling; the authors further linked these effects to lactate-to-pyruvate conversion and associated redox shifts (Yang et al. 2014). An important refinement is that lactate’s interaction with NMDARs appears state-dependent, instead of a uniform potentiator. A study focused on NR2B-containing NMDARs demonstrated a “dual action” framework: under lower glutamatergic drive, lactate potentiates NMDAR signaling through an NADH/redox-linked mechanism involving NR2B; under stronger glutamatergic stimulation, a distinct pathway (more consistent with energetic/pyruvate-linked protection) can dominate (Jourdain et al. 2018). This non-monotonicity is central when translating to BG: the same lactate transient that enhances adaptive plasticity during moderate, patterned corticostriatal activity could plausibly amplify pathological signaling if it coincides with excessive glutamatergic drive or weakened homeostatic buffering. Mechanistically, NR2B has a privileged position in coupling NMDAR activation to ERK. One seminal study showed that RasGRF1 binds NR2B and that disrupting this interaction impairs NMDAR-dependent ERK activation, offering a molecular bridge from receptor subunit composition to kinase coupling (Krapivinsky et al. 2003). Together, these data support a plausible synergy motif: lactate boosts NR2B-dependent NMDAR signaling; NR2B integrates effectively to Ras–ERK; ERK drives transcriptional and synaptic consolidation.

For the synergy hypothesis to be pivotal to D2-MSNs, NMDAR-dependent plasticity must be a meaningful control point in corticostriatal synapses. This is supported by direct striatal physiology: theta-burst stimulation can reliably induce corticostriatal LTP, accompanied by enhanced presynaptic and postsynaptic NMDAR activity; disrupting α2δ−1–NMDAR interactions (pharmacologically or genetically) abolishes this LTP and impairs striatum-dependent learning tasks (Zhou et al. 2018). This study is important because it reframes corticostriatal LTP as a synchronized pre/post NMDAR process with specific molecular coupling—specifically the kind of mechanism that could be sensitive to lactate-driven regulation of NMDAR gain. A second, disease-relevant layer is that NMDAR subunit composition at corticostriatal synapses is plastic and DA-sensitive. A striatal slice study demonstrated that manipulating NR2A/NR2B balance modifies MSN spine morphology, and that D1 receptor stimulation shifts NMDAR subunit composition in a manner that depends on NR2B for downstream structural effects; the authors explicitly link NMDAR subunit remodeling to experimental parkinsonism and corticostriatal dysfunction (Vastagh et al. 2012). Although this work does not test lactate, it provides a core constraint: the receptor substrate that lactate is most directly implicated in modulating (NR2B-linked signaling) is itself dynamically regulated in striatal disease-relevant states, making synergy plausible, but also possibly unstable across PD stages.

At the cell-type level, D2/iSPNs have corticostriatal synaptic features that could make them especially sensitive to shifts in NMDAR gain. In an influential study, excitatory synapses onto indirect-pathway MSNs exhibited higher release probability and larger NMDAR currents than those onto direct-pathway MSNs; the same study demonstrated that endocannabinoid-induced LTD is selectively expressed in indirect-pathway MSNs and needs D2 receptor activation (Kreitzer and Malenka 2007). These properties imply that, under comparable cortical input, iSPNs may experience stronger NMDAR-dependent Ca^2^⁺ signaling—creating a plausible substrate for lactate’s NMDAR-potentiating effects to be functionally consequential. However, it is important not to over-extend this logic. The best-supported iSPN plasticity specialization in that work is D2-dependent eCB-LTD, which is not necessarily identical to NMDAR-dependent LTP mechanisms; moreover, NMDAR-dependent plasticity rules can differ by striatal region, developmental stage, stimulation pattern, and neuromodulatory state (Zhou et al. 2018). Hence, the most defensible claim is conditional**:** if D2-MSNs learning rules in a given striatal domain depend substantially on NMDAR-dependent Ca^2^⁺/ERK coupling (as is true for corticostriatal LTP), then lactate has a credible route to modulate those rules by modifying NMDAR gain and ERK engagement.

Synergy should not be assumed to be unidirectional “enhancement.” D2-MSNs sit at the intersection of multiple second-messenger systems that can gate plasticity thresholds. Lactate can potentiate NMDAR signaling via redox/NADH mechanisms, which would be expected to increase Ca^2^⁺-dependent ERK activation under permissive glutamate patterns (Yang et al. 2014). Meanwhile, lactate can also signal via Gi-coupled HCAR1 in neurons (in systems where it is functionally expressed), which—by decreasing cAMP/PKA tone—could oppose several forms of NMDAR- or A2A-driven plasticity depending on pathway dominance. The net result in D2-MSNs may hence be better described as reweighting of upstream channels into ERK, instead of simple amplification (Jourdain et al. 2018). This is one reason the “dual action” lactate framework is especially useful for striatum: it predicts that lactate’s impact on NMDAR-dependent plasticity will be nonlinear with respect to glutamatergic drive and cellular metabolic state (Jourdain et al. 2018). In PD, where DA depletion modifies both synaptic gain and NMDAR subunit composition, these nonlinearities could become decisive—either enabling beneficial recalibration during structured exercise-related input patterns or, In contrast, promoting pathological signaling when network dynamics are pathological.

What is still missing for a strong D2-MSNs (iSPN) claim is not the general idea that lactate can potentiate NMDAR → ERK coupling—this is well supported in other neuronal contexts—but rather direct, cell-type–resolved causality in genetically identified iSPNs under controlled lactate dynamics (Yang et al. 2014). Key experiments are now feasible: combine D2-MSN ERK reporters with controlled lactate transients, matched pH/osmolarity, NMDAR blockade, and MCT inhibition (Yang et al. 2014); test whether lactate preferentially amplifies NR2B-linked signaling and whether NR2B → RasGRF1 disruption blunts ERK engagement (Krapivinsky et al. 2003); and determine whether lactate shifts corticostriatal LTP thresholds or persistence in D2-MSNs under PD-relevant NMDAR remodeling (Zhou et al. 2018).

Current main evidence supports a coherent synergy model: lactate can potentiate NMDAR currents and Ca^2^⁺ signaling and induce ERK-linked plasticity gene programs, with NR2B and RasGRF offering an established molecular bridge from NMDAR activation to ERK (Yang et al. 2014). Striatum provides a biologically credible target for this synergy because corticostriatal LTP depends on synchronized NMDAR activity and is required for striatum-dependent learning (Zhou et al. 2018). In addition, indirect-pathway MSNs display larger NMDAR currents and distinctive plasticity gates, conceptualizing D2-MSNs as a plausible locus where lactate could meaningfully shift plasticity thresholds (Kreitzer and Malenka 2007). The critical caveat remains that D2-MSN-specific lactate → NMDAR → plasticity causality has not been definitively demonstrated, and PD-related remodeling of NMDAR composition introduces a moving target that could flip the sign of “synergy” across disease states (Vastagh et al. 2012).

#### ERK-dependent functional outputs in D2-MSNs

In indirect-pathway D2 medium spiny neurons (D2-MSNs; iSPNs), ERK1/2 signaling is best viewed as a state-setting module that links moment-to-moment synaptic/neuromodulatory integration to longer-lived changes in neuronal excitability, synaptic structure, and activity-dependent gene expression. Although much of the striatal ERK literature has historically emphasized D1-MSNs–biased outputs in dyskinesia, there is now strong genetic evidence that ERK is not optional in D2-MSNs: it is required to maintain core electrophysiological and structural properties that recognize how the indirect pathway constrains movement (Hutton et al. 2017).

The clearest causal evidence comes from cell-type-specific genetic ablation. In a study that eliminated ERK/MAPK signaling in each MSN class, loss of ERK/MAPK signaling in D2R-MSNs produced a marked hyperlocomotor phenotype, accompanied by suppressed intrinsic excitability, decreased dendritic spine density, and suppressed activity-associated gene expression even after pharmacological stimulation (Hutton et al. 2017). This combination is mechanistically coherent: dampening the excitability and synaptic integration capacity of iSPNs weakens indirect-pathway suppression, biasing BG output toward disinhibition of movement (hyperlocomotion) despite decreased firing competence at the single-cell level (Hutton et al. 2017). Two implications follow for lactate–ERK hypotheses in PD. First, ERK in D2-MSNs is positioned upstream of both swift and long-term outputs: by maintaining membrane excitability and spine architecture, it constrains the “operating range” within which neuromodulators and metabolic cues (including lactate) can tune indirect-pathway gain (Hutton et al. 2017). Second, ERK-targeted interventions—whether pharmacological inhibitors or metabolic interventions that indirectly shift ERK tone—should be evaluated for bidirectional risk: chronic decrease of ERK may inhibit pathological signaling, but it may also erode D2-MSNs structural/functional integrity that is required for stable action selection (Hutton et al. 2017).

A second class of ERK-dependent outputs concerns immediate-early genes (IEGs) and the transcriptional programs that encode recent activity history. Acute D2R antagonism provides a tractable probe of this machinery. In vivo, haloperidol (D2R blockade) activates phosphorylation of ERK (and associated signaling markers, such as MSK1 and histone H3 in that paradigm) selectively in D2R-expressing striatal neurons, and induces IEGs, such as c-fos and zif268 primarily in that population (Bertran-Gonzalez et al. 2008). These results build an important point for D2-MSNs biology: ERK activation is not a “D1-only” phenomenon; it can be consistently engaged in the striatopallidal compartment and can couple to IEG induction under specific neuromodulatory conditions (Bertran-Gonzalez et al. 2008). However, the translation of phospho-ERK into chromatin-level transcriptional “writing” is context- and cell-type-dependent. In striatopallidal MSNs, haloperidol induces a rapid, maintained phosphorylation of histone H3 that depends on A2A receptor signaling, Gαolf, and PKA/DARPP-32, while ERK phosphorylation—although present—was not implicated as the driver of H3 phosphorylation in that setting; the study also highlights lower MSK1 levels in striatopallidal than striatonigral MSNs and reports unaltered H3 phosphorylation in MSK1 knockout mice (Bertran-Gonzalez et al. 2009). The critical inference is that ERK activation in D2-MSNs does not automatically imply nuclear transcriptional remodeling via canonical ERK → MSK1 → H3 routes; rather, D2-MSNs nuclear outputs can be shaped by parallel second-messenger logic (A2A–cAMP–DARPP-32–PP1) that may operate with, alongside, or even independently of ERK (Bertran-Gonzalez et al. 2009). A related example of D2-MSNs-biased transcriptional output is the induction of Nur77 following haloperidol, observed as occurring primarily in striatopallidal MSNs and linked to DARPP-32/PP1 regulation (Sánchez et al. 2014). Together, these studies support a layered model: ERK participates in D2-MSNs activity-dependent transcription, but the dominant transcriptional route may be altered depending on the balance of D2R inhibition, A2A activation, and glutamatergic co-drive.

The D2-MSNs deletion phenotype (reduced spine density) implies that ERK signaling contributes to the maintenance of excitatory synaptic structure (Hutton et al. 2017). This is consistent with the general role of ERK in coordinating cytoskeletal regulation, local translation, and synapse-to-nucleus signaling. Yet a specific gap remains for a “lactate → ERK → plasticity” claim in D2-MSNs: we still lack systematic mapping of which corticostriatal plasticity rules (eCB-LTD vs NMDAR-dependent LTP/LTD) are ERK-dependent within identified iSPNs across striatal subregions and disease states (Hutton et al. 2017). This gap is especially important in PD, where DA depletion reshapes plasticity thresholds and circuit state, possibly converting ERK engagement from adaptive consolidation into pathological state-locking.

A final point of rigour is to prevent conflating “striatal ERK” with “D2-MSN ERK” in PD contexts. In hemiparkinsonian mice, L-DOPA induces ERK phosphorylation (and downstream markers, such as MSK1 and histone H3 phosphorylation) primarily in striatonigral (D1R) MSNs, with no apparent ERK signaling activation in striatopallidal (D2R) MSNs in that model; D1R antagonism blocks this effect (Santini et al. 2009). This does not diminish the importance of ERK in D2-MSN physiology (as reported by genetic deletion), but it does imply that therapeutically relevant ERK outputs may be cell-type- and stimulus-specific: dyskinesia-linked ERK programs may be D1-biased, in contrast to D2-MSN ERK may be more central for baseline indirect-pathway competence, A2A/D2-driven transcriptional responses, and possibly metabolic modulators, such as lactate.

The strongest established ERK-dependent outputs in D2-MSNs are (i) maintenance of intrinsic excitability and dendritic spine architecture that calibrate indirect-pathway gain, and (ii) engagement of activity-dependent gene expression modules under specific neuromodulatory conditions, with nuclear epigenetic outputs often gated by parallel A2A–cAMP–DARPP-32 pathways (Hutton et al. 2017). For this review, the key endpoints are not pERK levels alone, but lactate-linked changes in D2-MSN excitability, synaptic structure, and transcriptional programs that gate indirect-pathway hypokinesia (Hutton et al. 2017). Broad ERK suppression should therefore be treated cautiously because it may weaken iSPN structural competence.

#### Therapeutic potential and the balance between adaptive and pathological plasticity

Any proposal to therapeutically harness lactate–ERK/MAPK signaling in D2-MSNs must confront a central paradox: ERK is simultaneously a substrate for adaptive plasticity and a driver of pathological plasticity when engaged in the wrong cell type, at the wrong time, or with the wrong kinetics. In the striatum, ERK sits at the convergence of NMDAR-induced Ca^2^⁺ signals, dopamine/adenosine second-messenger states, and metabolic/redox constraints. Lactate can plausibly modulate each of these inputs—through MCT-dependent redox shifts that potentiate NMDAR signaling (Yang et al. 2014), and via HCAR1-dependent Gi signaling that can reweight cAMP/PKA tone and synaptic release probability (de Castro Abrantes et al. 2019). The therapeutic question is hence not whether lactate “activates ERK”, but whether lactate can be used to bias ERK outputs toward restorative, circuit-stabilizing programs without crossing into pathological reinforcement.

A critical constraint comes from pathway- and cell-type-specific genetics. In mice with selective disruption of ERK/MAPK signaling in D2R-expressing MSNs, investigators uncovered marked alterations in D2-MSN excitability, dendritic spine density, and activity-regulated gene expression, accompanied by marked changes in locomotor behavior (notably hyperlocomotion) consistent with a loss of indirect-pathway suppressive efficacy (Hutton et al. 2017). This result is easy to miss in PD discussions that focus on ERK as a “pathology readout”: in D2-MSNs, chronic ERK inhibition is itself predicted to destabilize circuit function, possibly decreasing the fidelity of action inhibition and selection even if it dampens several pathological gene programs. The therapeutic implication is rigorous: non-selective ERK inhibition is unlikely to be safe as a long-term strategy, and any metabolic intervention that chronically depresses ERK in D2-MSNs risks eroding the structural and electrophysiological substrate required for normal indirect-pathway computation (Hutton et al. 2017).

In DA-depleted striatum, L-DOPA consistently activates ERK signaling and histone H3 phosphorylation specifically in striatonigral (D1) MSNs, and this pattern is tightly associated with dyskinetic states; D1 receptor blockade prevents the ERK activation (Santini et al. 2009). This establishes a clear disease-relevant hazard: any intervention that globally amplifies NMDAR → ERK coupling in the striatum could, in principle, potentiate D1-MSN ERK programs that induce pathological plasticity underlying L-DOPA–induced dyskinesia (LID). This risk is mechanistically credible for lactate because the best-supported lactate-to-ERK route in neurons involves potentiation of NMDAR signaling and downstream ERK1/2 activation (Yang et al. 2014). If lactate transients occur during L-DOPA dosing or during periods of abnormal corticostriatal drive, lactate could plausibly act as a gain factor that increases the likelihood that NMDAR-triggered ERK signals reach transcriptional thresholds in D1-MSNs, thereby accelerating pathological consolidation. The present evidence does not prove this interaction in vivo; however, the cell-type selectivity of L-DOPA–ERK signaling makes it a non-trivial safety boundary that must be explicitly addressed in any lactate-based therapeutic narrative (Santini et al. 2009).

Transient lactate pulses (e.g., during exercise) may act as instructive signals that support adaptive remodeling, in contrast to sustained lactate elevation (e.g., from chronic glycolytic reprogramming, inflammation, or weakened clearance) may help buffer pathological metabolic–inflammatory states. Main datasets illustrate why this distinction matters: pulse-like lactate signaling can be beneficial because exercise (and lactate) activates HCAR1, increasing cerebral VEGFA and promoting angiogenesis in an HCAR1-dependent manner, offering a receptor-defined route whereby biological lactate transients can trigger longer-term support programs even outside a striatum-specific context (Morland et al. 2017). In PD models, chronic lactate dysregulation may be harmful: SIRT1–PKM2-mediated restoration of lactate homeostasis delayed parkinsonism, whereas brain lactate elevation recapitulated PD-like phenotypes (Lian et al. 2024); independently, glycolysis-derived lactate promoted microglial H3K9la activation and dopaminergic injury, while glycolysis inhibition reduced lactate accumulation and improved outcomes (Qin et al. 2025). Applied to D2-MSN ERK–focused therapeutics, this proposes for a narrow optimization target: use controlled, time-limited lactate signaling to bias D2-MSN plasticity toward adaptive recalibration, while avoiding chronic lactate elevation that may promote neuroinflammation, lactylation-dependent state fixation, and non-selective (or persistently engaged) ERK outputs (Lian et al. 2024; Qin et al. 2025).

Lactate interventions should be time-limited and synchronized with structured corticostriatal activation, because lactate-engaged ERK signaling is most relevant when NMDAR-dependent plasticity programs are active; continuous or poorly timed exposure could overlap with ERK-sensitized dyskinetic states (Yang et al. 2014; Santini et al. 2007). ERK output in D2-MSNs is also tightly gated by D2R–A2A second-messenger balance: acute D2R antagonism with haloperidol can selectively induce ERK phosphorylation and immediate-early gene induction in D2R-expressing (striatopallidal) neurons, while A2A/cAMP/DARPP-32 signaling shapes downstream nuclear responses—underscoring how neuromodulatory disinhibition can dramatically raise ERK gain in iSPNs and hence must be accounted for alongside concurrent pharmacology (D2R antagonists/partial agonists, A2A antagonists, L-DOPA regimens) (Bertran-Gonzalez et al. 2008, 2009). Finally, “balance” need not mean “more plasticity”: HCAR1 activation can suppress neuronal network activity via Gα and Gβγ-dependent mechanisms and shows functional interactions with other inhibitory GPCRs, motivating a coherent “brake-first” agenda where HCAR1-biased lactate signaling is used to damp exorbitant excitatory drive and limit ERK overshoot in vulnerable states, possibly complementing (instead of only amplifying) redox–NMDAR potentiation (de Castro Abrantes et al. 2019).

Exercise can reduce dyskinesia severity and modulate striatal ERK1/2 phosphorylation in PD mouse models (in studies explicitly examining aerobic training and LID), suggesting that behavioral interventions can reparameterize ERK-linked plasticity in the DA-depleted striatum (Zhou et al. 2024). Nevertheless, these data do not isolate lactate as the mediator; exercise changes numerous variables (dopamine dynamics, adenosine tone, neurotrophins, inflammation). For a pathophysiological claim, lactate causality would need demonstrating that blocking lactate transport/sensing selectively prevents the ERK and behavioral benefits of exercise, without broadly impairing training capacity.

The therapeutic promise of lactate–ERK/MAPK modulation in D2-MSNs is best framed as precision control of a plasticity gateway: support D2-MSNs structural and excitability competence (which needs intact ERK signaling) (Hutton et al. 2017), while avoiding non-specific amplification of dyskinesia-linked ERK programs in D1-MSNs (Santini et al. 2009) and avoiding chronic lactate homeostatic collapse that can buffer neuroinflammatory states through lactate/lactylation pathways (Lian et al. 2024). This balance suggests that timing, dose, and cellular compartment—instead of absolute lactate elevation—will determine whether lactate-driven signaling yields adaptive rehabilitation or pathological state locking.

#### Summary

Collectively, the evidence reviewed in Sects. "Hypothesized routes by which lactate could influence ERK/MAPK signaling in D2-MSNs" through "Therapeutic potential and the balance between adaptive and pathological plasticity" supports a mechanistically coherent, but incompletely tested framework where lactate can interface with D2-MSNs signaling via ERK/MAPK as a central integrator. The strongest molecular scaffold is the MCT-dependent metabolic/redox route, whereby lactate uptake and conversion to pyruvate modifies intracellular redox state to potentiate NR2B-linked NMDAR signaling, thereby increasing the probability and/or persistence of downstream Ras–ERK coupling and activity-regulated gene programs (Yang et al. 2014). A second, plausible, but less firmly localized route is HCAR1-induced GPCR signaling, which can suppress network activity via Gi/Gβγ mechanisms and may bias ERK compartmentalization and output instead of simply increasing nuclear transcription (de Castro Abrantes et al. 2019). These inputs are embedded in the distinctive D2-MSNs second-messenger landscape, where D2R–A2A antagonism and adenosine dynamics gate cAMP/PKA tone and can strongly gate ERK responsiveness and transcriptional outcome (Ma et al. 2022).

ERK signaling in D2-MSNs is functional, not merely descriptive: cell-type-specific ERK/MAPK disruption alters intrinsic excitability, spine structure, and behavior (Hutton et al. 2017). Thus, broad ERK suppression may reduce pathological signaling but also impair indirect-pathway competence. Meanwhile, the DA-depleted striatum expresses a well-established hazard regime where L-DOPA drives prominent ERK programs in D1-MSNs linked to dyskinesia; therefore, lactate-driven amplification of NMDAR–ERK gain could be beneficial or harmful depending on timing, compartment, and the relative engagement of D1- versus D2-MSNs ensembles (Santini et al. 2009).

Accordingly, the most defensible therapeutic framing is a window model: transient, state-coupled lactate signaling (for instance during structured exercise) may help recalibrate D2-MSN plasticity and support circuit stabilization, in contrast to maintained lactate homeostatic collapse—linked to neuroinflammation and lactylation-dependent state fixation in PD models—may push the system toward pathological plasticity and treatment-refractory network states (Qin et al. 2025). The decisive next step is methodological instead of conceptual: demonstrate, in genetically identified D2-MSNs, that physiologically realistic lactate transients engage ERK with specific kinetics and compartmentalization, and report that perturbing lactate transport/sensing selectively shifts D2-MSN plasticity rules and PD-relevant behavior without exacerbating D1-MSN ERK programs.

### Crosstalk between lactate signaling and DA D2 receptor function

The potential crosstalk between lactate signaling and dopamine D2 receptor (D2R) function should be framed as a hypothesis-generating interface rather than as an established basal ganglia mechanism. The rationale is limited but biologically coherent: D2Rs are canonical Gi/o-coupled receptors in striatal indirect-pathway neurons and dopaminergic terminals, whereas HCAR1 is a lactate-sensitive Gi/o-coupled receptor. Both pathways can influence cAMP/PKA-dependent gain control, but they encode different biological variables. D2Rs encode dopamine transmission with high spatial and temporal specificity, whereas HCAR1 is more plausibly a slower metabolic-state sensor engaged during elevated extracellular lactate. Therefore, any proposed convergence between lactate–HCAR1 and dopamine–D2R signaling should be treated as a testable analogy, not as evidence that HCAR1 substitutes for D2R signaling in PD.

#### Shared inhibitory logic via Gi/o-coupled signaling

A limited analogy can be drawn between lactate–HCAR1 signaling and D2R signaling because both engage Gi/o-coupled GPCR pathways. D2Rs operate postsynaptically on indirect-pathway SPNs and presynaptically as autoreceptors on dopaminergic terminals, whereas lactate deorphanization studies established GPR81/HCAR1 as a lactate-sensitive receptor capable of Gi-dependent inhibition of adenylyl cyclase (Gerfen and Surmeier 2011; Liu et al. 2009). At the pathway level, both receptor systems can decrease cAMP signaling and thereby influence PKA-dependent phosphorylation programs that regulate membrane excitability, synaptic function, and striatum-dependent learning (Bonito-Oliva et al. 2014; Kheirbek et al. 2009). D2R signaling has established basal ganglia relevance, including effects on iSPN excitability, presynaptic dopamine release, and phasic receptor activation (Hernandez-Lopez et al. 2000; Benoit-Marand et al. 2001; Marcott et al. 2014). HCAR1 activation can also suppress neuronal activity through Giα- and Gβγ-dependent mechanisms in non-basal-ganglia preparations (de Castro Abrantes et al. 2019). However, this shared intracellular logic does not demonstrate that HCAR1 is functionally engaged in identified D2-MSNs or that lactate recruits a D2R-like brake in PD striatum.

Despite partial convergence at cAMP-linked signaling nodes, D2R and HCAR1 differ in what they encode. D2R signaling is tuned to dopamine’s local spatiotemporal profile and can show region-specific coupling properties (Marcott et al. 2018). By contrast, lactate is more likely to act as a volume or metabolic-state signal, with HCAR1 engagement depending on extracellular lactate concentration, receptor localization, and exposure duration (Morland et al. 2015). Thus, HCAR1 should not be described as a surrogate D2 receptor. It is better described as a candidate metabolic input that could, under defined conditions, modulate signaling in circuits already shaped by D2R/A2A balance.

#### Synchronized inhibition: increasing signal fidelity and stability

The idea of synchronized inhibition should be presented as a model to test, not as a demonstrated feature of basal ganglia physiology. D2 autoreceptors provide rapid negative feedback on dopamine release, and in vivo measurements show that this feedback can shape dopamine transients on subsecond timescales (Benoit-Marand et al. 2001). Optical and photopharmacological studies further indicate that synaptically released dopamine interacts with D2 autoreceptors within a brief and spatially constrained window (Condon et al. 2021). HCAR1 operates on a different scale: receptor-site mapping supports the presence of lactate-sensitive signaling sites in brain tissue, and HCAR1 activation can reduce cAMP signaling and neuronal activity in systems where the receptor is functionally engaged (de Castro Abrantes et al. 2019; Lauritzen et al. 2014). Genetically encoded dopamine sensors such as dLight further emphasize that dopaminergic information is carried by rapid, spatially resolved transients, whereas lactate signaling is more likely to report slower metabolic state (Patriarchi et al. 2018).

This distinction narrows the claim. Lactate–HCAR1 signaling might modulate circuit gain during elevated-lactate states, but it is unlikely to encode the same information as phasic dopamine. If HCAR1 is functionally expressed in relevant striatal compartments, transient lactate elevations could in principle add a slower inhibitory or neurovascular context signal. Conversely, chronic lactate elevation could reduce dynamic range or worsen hypokinetic network states. Direct support for either possibility in PD basal ganglia is still absent. The necessary experiments are clear: measure striatal lactate, dopamine transients, and D2-MSN activity together, then test whether HCAR1 perturbation changes dopamine signal fidelity, beta synchronization, or motor output.

#### State dependence in PD and during exercise interventions

PD changes the signaling context in which any lactate–HCAR1 effect would occur. Dopamine depletion weakens D2R-dependent inhibitory control in indirect-pathway circuits and shifts basal ganglia output toward hypokinetic states (Kravitz et al. 2010). At the ensemble level, both direct- and indirect-pathway SPNs can be recruited around action initiation, indicating that PD disrupts coordinated action selection rather than simply removing a movement-facilitating signal (Cui et al. 2013). Within this altered state, a lactate-sensitive Gi/o pathway could theoretically influence cAMP/PKA tone or synaptic gain, but this remains inferential unless HCAR1 is shown to be functionally engaged in identified striatal cell types.

Exercise provides a useful but still indirect setting for this hypothesis. Lactate–HCAR1 signaling can mediate selected exercise-linked CNS adaptations such as VEGFA induction and angiogenesis in mice (Morland et al. 2017), and human physiology shows that circulating lactate can enter and be oxidized by the brain when blood lactate rises during infusion or exercise (Hall et al. 2009). Clinical and translational PD studies support the broader relevance of exercise intensity and striatal adaptation: SPARX showed feasibility and a motor signal for high-intensity treadmill exercise in de novo PD (Schenkman et al. 2018), Park-in-Shape demonstrated exercise-related changes in putaminal connectivity (Johansson et al. 2022), and PD mouse studies indicate that aerobic training can alter striatal beta activity, MSN firing, D2-MSN-related abnormalities, and D2 receptor expression (Wang et al. 2024). These findings are compatible with lactate involvement, but they do not show that lactate or HCAR1 mediates exercise-induced striatal rescue.

The most defensible interpretation is therefore conditional. Transient, well-cleared lactate elevations during exercise may provide substrate, receptor, vascular, or redox-linked signals that interact with dopaminergic state. Chronic lactate elevation in PD, by contrast, may reflect mitochondrial constraint, inflammatory glycolytic reprogramming, or impaired clearance, and could become maladaptive. To close the evidence gap, future studies should determine whether exercise-induced striatal lactate reaches HCAR1-relevant concentrations, whether HCAR1 perturbation alters exercise-driven normalization of D2-MSN activity or beta synchrony, and whether these effects can be separated from broader training adaptations. Combining lactate sensors, dopamine sensors such as dLight, and cell-type-specific perturbations would allow this model to be tested directly (Patriarchi et al. 2018).

#### Toward combined metabolic–neurotransmitter co-therapies

The translational implication of lactate–D2R crosstalk should remain conservative. PD treatment already targets neurotransmitter state dynamically through levodopa dosing, management of OFF/ON fluctuations, and modulation of indirect-pathway signaling. Locomotion itself can recruit striatal PKA signaling through coordinated dopamine and adenosine dynamics, emphasizing that D2R/A2A/cAMP balance is state dependent (Ma et al. 2022). Clinically, A2A receptor antagonism provides proof that non-dopaminergic modulation of indirect-pathway signaling can reduce OFF time when used as add-on therapy to levodopa (Lewitt et al. 2008). This supports the broader principle that indirect-pathway signaling can be therapeutically modulated, but it does not imply that HCAR1 agonism or lactate elevation can substitute for dopaminergic or A2A-directed therapy.

A lactate-informed co-therapy framework should therefore be framed as a monitoring and experimental strategy rather than a ready treatment. HCAR1 activation can downmodulate neuronal activity in non-basal-ganglia systems (de Castro Abrantes et al. 2019), and lactate–HCAR1 signaling can support selected neurovascular adaptations (Morland et al. 2017). These observations justify testing whether lactate kinetics influence the response to exercise or rehabilitation, but they do not establish HCAR1 as a PD therapeutic target. At the same time, chronic lactate dysregulation may contribute to inflammatory or lactylation-linked microglial states in PD models (Qin et al. 2025), and restoration of lactate homeostasis through upstream glycolytic control has been reported to alleviate parkinsonian phenotypes (Lian et al. 2024). These findings argue against indiscriminate lactate elevation.

A practical translational model should therefore separate two aims: first, use controlled exercise or rehabilitation to generate time-limited lactate exposure while monitoring clearance and clinical response; second, prevent or reverse chronic lactate homeostatic collapse when it reflects inflammatory or mitochondrial stress. This approach preserves the potential value of lactate as a state and response marker without treating HCAR1 as a proven basal ganglia therapy. Any future HCAR1-directed intervention would need to demonstrate receptor expression and function in defined striatal cell types, causal effects on D2-MSN physiology or dopamine signaling, and motor benefit without worsening hypokinesia, dyskinesia, or cognitive flexibility.

## Toward precision intervention: exercise intensity, lactate threshold, and PD

### Exercise intensity–dependent lactate kinetics

#### Low-intensity exercise: steady-state lactate clearance and metabolic coordination

Low-intensity exercise (typically below the first lactate threshold and well below maximal lactate steady state (MLSS) is defined by a near steady-state relationship between lactate appearance (production/release into blood) and lactate disappearance (uptake/oxidation or conversion), such that blood lactate concentration remains close to resting values or rises only modestly. The core pathophysiological point is that stable blood lactate does not imply low lactate flux: even when concentration changes are small, lactate turnover can increase substantially because both appearance and disappearance rates are upregulated Meanwhile with metabolic rate and blood flow. This distinction—concentration versus flux—is central to interpreting low-intensity training, where the dominant adaptation signal may be improved lactate handling instead of large lactate excursions (Stanley and Brooks 1987). A main tracer study in humans displays this steady-state logic directly. utilizing a primed continuous infusion of radiolabeled lactate during graded exercise, Stanley and colleagues quantified systemic lactate kinetics and demonstrated that near steady-state values of lactate appearance and disappearance emerged at the lower work rates, with both rates rising above resting levels while blood lactate remained comparatively constrained. Importantly, the lactate metabolic clearance rate increased from rest during these lower intensities, confirming that the organism proactively raises its capacity to remove lactate as exercise begins—well before any marked lactatemia is uncovered (Stanley and Brooks 1987).

mechanistically, low-intensity exercise recruits lactate clearance via synchronized increases in perfusion, transporter-induced exchange (MCT-dependent shuttling), and mitochondrial oxidation capacity across multiple “recipient” tissues. In humans, tracer-based work utilizing labeled lactate has emphasized that oxidation is the major fate of lactate removal during exercise, and—crucially—that blood lactate concentration alone is a poor proxy for the magnitude of lactate disposal and oxidation. This supports a reframing of low-intensity exercise: it is a condition where lactate is continuously produced and continuously consumed, but the system remains below the tipping point at which appearance outpaces disappearance (Mazzeo et al. 1985). A complementary dual-tracer study at moderate-but-still submaximal intensity offers additional quantitative insight into this “high flux, low concentration” regime. During ~ 43% VO₂max cycling, lactate appearance increased, clearance roughly doubled by ~ 25 min, and blood lactate remained low; about 20% of glucose utilization entered lactate formation, confirming lactate as a routine aerobic intermediate (Stanley et al. 1988). Although this experiment sits tighter to “moderate” than “very low” intensity, it clarifies a principle that applies at low intensities: lactate shuttling and recycling are integral to aerobic metabolism, not exceptional events limited to severe effort.

Low-intensity steady state also depends on the capacity of non-muscle beds to remove lactate and to integrate lactate with systemic glucose homeostasis. Classic human work measuring splanchnic exchange during prolonged exercise shows that the splanchnic bed takes up lactate and pyruvate during exercise, encouraging the view that lactate is a major gluconeogenic precursor and a circulating intermediary linking working muscle to hepatic glucose output—notably as exercise duration increases (Rowell et al. 1966). For pathophysiological interpretation, this matters because low-intensity exercise is often maintained long enough for these slower endocrine–metabolic coordination mechanisms (hepatic uptake, gluconeogenesis, substrate repartitioning) to turn into quantitatively meaningful, even without large lactate accumulation.

A robust conclusion from training physiology is that endurance adaptation often manifests more strongly as enhanced lactate clearance than as decreased lactate generation at a given workload. In a seminal study where lactate kinetics were quantified in trained versus untrained conditions, endurance training enhanced metabolic clearance of lactate during exercise, encouraging the concept that “fitness” shifts the system toward greater lactate disposal capacity (via oxidative use and/or other clearance routes), thereby decreasing lactate accumulation as intensity rises (Donovan and Brooks 1983). This aids clarify why low-intensity training can be metabolically meaningful even when lactate remains low: repeated exposure to a steady-state regime preferentially trains the removal architecture (transport, oxidation, perfusion matching) that determines where the lactate curve bends upward at higher intensities.

For PD, low-intensity exercise should be interpreted with disciplined expectations. Because lactate typically remains near baseline in this regime, lactate-as-a-signal (HCAR1 engagement; large lactate pulses) is less likely to be the main pathophysiological driver during the exercise bout itself. Instead, the most defensible lactate-related contribution is indirect: low-intensity exercise consistently recruits the biological machinery that maintains lactate steady state—increasing systemic clearance capacity and oxidative coordination—thereby increasing metabolic headroom and possibly decreasing the likelihood that stressors push BG tissue into pathological lactate/acid–redox regimes (Stanley and Brooks 1987). Meanwhile, this is specifically why low intensity is attractive as a foundation for personalized exercise prescription: it is generally tolerable, supports high-volume repetition, and trains clearance and coordination without imposing large perturbations that could be problematic in individuals with decreased metabolic reserve or fluctuating medication states.

The lactate steady-state concept is sometimes operationalized utilizing MLSS-derived frameworks, but MLSS is a high steady-state boundary specific by the highest constant workload at which lactate does not continue to rise over time (for instance, ≤ 1 mmol·L⁻^1^ increase over the last 20 min of a constant-load bout in a commonly used definition) (Heck and Wackerhage 2024). Low-intensity exercise sits far below this boundary. Therefore, it is methodologically inappropriate to infer pathophysiological engagement of “threshold-like” lactate signaling from low-intensity prescriptions unless lactate (or at least intensity in comparison with individual thresholds) is actually measured.

Overall, low-intensity exercise is best conceptualized as a high-coordination metabolic state: lactate production persists, lactate clearance is proactively upregulated, and multiple tissue beds cooperate to keep lactate concentration stable. The pathophysiological value of this regime—notably for PD translation—is less about lactate peaks and more about training the clearance and oxidative sink that determines whether higher-intensity bouts (and daily-life demands) are experienced as adaptive, well-buffered challenges or as destabilizing metabolic stress (Stanley and Brooks 1987).

#### Moderate-intensity exercise: threshold crossing and adaptive lactate signaling

Moderate-intensity exercise is the regime where lactate transitions from a mainly “buffered” intermediate to a measurable systemic signal. Operationally, it spans workloads that approach and cross the first lactate/ventilatory breakpoint and expand toward (but do not necessarily exceed) the highest workload compatible with lactate steady state. Classic gas-exchange physiology formalized an “anaerobic threshold” concept as the intensity at which respiratory variables shift in a manner consistent with enhanced non-oxidative carbohydrate flux and acid–base disturbance (Wasserman et al. 1973). Because terminology and detection methods vary (blood lactate breakpoints, ventilatory thresholds, fixed-concentration criteria), “threshold crossing” is best framed as a phenomenological transition—a loss of strict proportionality between lactate appearance and lactate disappearance—instead of as a single invariant biological event.

As intensity increases into the moderate domain, blood lactate commonly rises from near-resting values to low millimolar plateaus. Below the first breakpoint, lactate concentration can remain stable despite high flux; above it, the system progressively relies on glycolysis in active fibers and on distributed shuttling/oxidation across tissues, such that lactate concentration becomes more sensitive to small mismatches between generation and clearance. A historically influential operational definition—the “4 mmol·L⁻^1^ lactate threshold”—was proposed and well-substantiated as a pragmatic marker for endurance performance contexts (Heck et al. 1985). Importantly, this fixed-concentration threshold is not equivalent to maximal lactate steady state (MLSS) in all individuals or sports; rather, it is a population-level heuristic that can misclassify the true steady-state boundary (Beneke and Duvillard 1996). MLSS is defined by constant-load tests showing that blood lactate remains stable at or below a given workload but rises progressively above it (Beneke and Duvillard 1996). Mechanistically, moderate intensity is best described by kinetics: sustained lactate elevation, often ~ 2–5 mM depending on protocol and fitness, may engage signaling without entering the severe acid–ion stress regime.

Moderate-intensity exercise offers a lactate exposure profile that is well suited to signaling for three reasons. Circulating lactate becomes available to the brain in a quantitatively meaningful way. In conscious humans, elevating arterial lactate (via infusion and/or exercise) switches the brain from net lactate generation to net lactate uptake and oxidation, with lactate contributing an increasing fraction of cerebral energy expenditure as blood lactate rises (Hall et al. 2009). Although the largest effects occur under higher lactatemia, the core implication for moderate intensity is permissive: even modest systemic lactate elevations can increase brain lactate availability and, therefore, the opportunity for lactate to act as a substrate and a signal. In addition, lactate can activate dedicated receptor pathways that are consistent with a homeostatic “metabolic governor.” Lactate receptor sites have been mapped in brain, and the receptor is proposed to function as a volume-transmitted link among neurotransmission, substrate availability, and neurovascular coupling (Lauritzen et al. 2014). In neuronal preparations, HCAR1 activation downmodulates network activity via Giα and Giβγ signaling and can interact functionally with other inhibitory GPCR systems, encouraging the plausibility of a buffering brake that is engaged when extracellular lactate is elevated (de Castro Abrantes et al. 2019). Moderate intensity—by generating maintained low-millimolar lactate—sits tighter to the receptor’s expected operating regime than low intensity, while typically avoiding the large acid loads that dominate at very high intensities. Furthermore, lactate is progressively supported as an “exerkine-like” mediator for plasticity-linked gene programs. In mice, lactate produced during exercise crosses the BBB, induces hippocampal Bdnf expression, and promotes learning and memory, with pathophysiological coupling to TRKB signaling (Hayek et al. 2019). Complementing this, an acute rodent study demonstrated that prolonged low-speed uphill exercise—despite low running speed—can elevate lactate in blood and brain and enhancement BDNF protein in cortex and hippocampus, displaying that moderate metabolic load (e.g., recruitment of glycolytic fibers) can be sufficient to produce central lactate/BDNF signatures. These studies do not establish that moderate-intensity lactate signals are the dominant mediator of PD motor benefit, but they provide main evidence that lactate can act upstream of neurotrophic pathways under physiologically plausible exposure patterns.

Two cautions are essential when importing this “adaptive lactate signaling” window into PD exercise prescription. Threshold definitions are not pathophysiological guarantees. Lactate breakpoints derived from incremental tests (including fixed 4 mmol criteria) were developed for performance diagnostics and can diverge from MLSS, ventilatory thresholds, and—most importantly—brain exposure (Heck et al. 1985). Hence, prescribing “moderate intensity” without direct lactate measurement can miss the intended signaling regime, especially in PD where autonomic function, medication state, and movement economy can shift biological responses. In addition, more lactate is not necessarily better in PD. PD models progressively suggest that chronic lactate homeostatic collapse can be associated with neuroinflammation and pathological transcriptional programs, meaning that the same metabolite may be adaptive when delivered as a controlled pulse, but pathological when persistently elevated (Hall et al. 2009). This strengthens the case for moderate intensity as a possibly safer signaling window than severe intensity in several patients: it may deliver maintained lactate exposure sufficient for receptor/transcription engagement while minimizing the probability of entering acid–ion and oxidative overload regimes.

Taken together, moderate-intensity exercise is best conceptualized as a threshold-sensitive coordination state: lactate becomes a maintained systemic variable that can (i) increase brain lactate availability (Hall et al. 2009), (ii) plausibly recruit inhibitory and neurovascular lactate receptor pathways (Lauritzen et al. 2014), and (iii) engage neurotrophic gene programs in vivo under appropriate exposure patterns (Hayek et al. 2019). The decisive PD-specific step—still mainly missing—is to demonstrate, with striatal readouts, that operating near personalized lactate thresholds generates lactate kinetics that causally normalize D2-MSNs state and corticostriatal plasticity without amplifying pathological dopaminergic plasticity in the DA-depleted striatum.

#### High-intensity exercise: peak lactate load and tolerance remodeling

High-intensity exercise (HIE)—operationally, work rates at or above the maximal lactate steady state (MLSS) and/or interval formats that consistently approach the “red zone” of cardiorespiratory strain—pushes lactate kinetics into a qualitatively different regime: lactate appearance exceeds lactate disappearance for maintained periods, so blood lactate no longer stabilizes, but accumulates increasingly during the bout and peaks during early recovery. MLSS itself is generally specific as the highest constant-load intensity at which blood lactate does not rise by more than ~ 1 mmol·L⁻^1^ over the final ~ 20 min of a 30-min constant-load test; by definition, HIE exceeds this boundary and hence induces non–steady-state lactate behavior (Heck and Wackerhage 2024).

In supramaximal efforts lasting ~ 30–120 s (e.g., Wingate-type tests and all-out sprint bouts), peak blood lactate concentrations of roughly ~ 15–25 mM are generally uncovered, typically 3–8 min post-exercise instead of immediately at cessation—implying emerging efflux from working muscle and distribution kinetics (Goodwin et al. 2007). This delayed peak is not a methodological footnote; it defines the temporal exposure of central tissues (including brain) to lactate pulses and hence the plausible window for lactate-sensitive receptor and transcriptional systems to be engaged after high-intensity work. A critical interpretive caveat—notably pivotal to the “lactate harms” sections—is that high lactate generally co-varies with acid–base disturbance and catecholaminergic drive. Hence, “peak lactate load” should be framed as a composite marker of a high glycolytic/turnover state instead of as a single causal variable; pathophysiological attribution needs pH-matched controls and/or transporter/receptor-specific perturbations (as discussed in Sects. "Acidification and disruption of ionic homeostasis" through "Disruption of DA transmission and receptor function") (Goodwin et al. 2007).

Human physiology offers direct evidence that raising arterial lactate into the mid–single-digit millimolar range shifts the brain from net lactate generation to net uptake and oxidation, and that this uptake increases further during exercise. In an integrated lactate-infusion plus cycling paradigm, arterial lactate was experimentally clamped from ~ 0.9 mM (rest) to ~ 3.9 mM (infusion) and ~ 6.9 mM during exercise, with substantial cerebral lactate uptake and oxidation (Hall et al. 2009). While these data are not specific to supramaximal sprint peaks, they build an essential enabling point: the brain can metabolically “see” systemic lactate rises well within the scope that overlaps moderate-to-high intensity exercise. Meanwhile, lactate’s signaling competence under high-intensity condition is supported by causal receptor genetics: high-intensity interval exercise and systemic lactate exposure increase brain VEGFA and capillary density in wild-type mice, but not in Hcar1 knockout mice, identifying lactate–HCAR1 as an initiating signal for a durable neurovascular adaptation program (Morland et al. 2017). This provides a mechanistically credible route whereby repeated high lactate pulses could cause long-term “tolerance remodeling” in the CNS—distinct from immediate fuel provision.

The hallmark of high-intensity training is not simply repeated lactate peaks; it is adaptive remodeling that modifies lactate kinetics and acid handling for the same external workload. Sprint training and high-intensity programs can increase monocarboxylate transporter expression (MCT1 and/or MCT4) in skeletal muscle, and MCT content has been mechanistically linked to faster post-supramaximal lactate removal (Bickham et al. 2006). The evidence is strongest for MCT1 increase and more variable for MCT4 depending on protocol, tissue, and sampling timing—a reminder that “lactate tolerance” is not a single uniform phenotype (Benítez-muñoz et al. 2024). Repeated sprint training can increase bicarbonate buffering capacity and improve high-intensity exercise tolerance, consistent with the idea that HIE trains not only substrate turnover, but also the biological systems that defend pH and ionic stability during glycolytic surges. These buffering adaptations are mechanistically pivotal to PD translation because they may reduce the probability that lactate-associated episodes cross into the acid/ion homeostasis failure regime emphasized in (Sect. "Acidification and disruption of ionic homeostasis") (小田啓之, 西脇雅人, 黒部一道, et al. 2023). A core conceptual refinement follows: successful high-intensity training tends to convert “peak lactate load” from a destabilizing metabolic stressor into a tolerable, time-bounded signal, by increasing distribution, oxidation, and clearance pathways and by increasing buffering headroom. In other words, the adaptive goal is not the elimination of lactate peaks, but the decrease of their pathological correlates (prolonged acidosis, ion pump compromise, inflammatory drift).

High-intensity endurance exercise is feasible in early, unmedicated PD and has reported a clinical signal in a phase 2 randomized trial (SPARX), motivating high-intensity regimens as candidate disease-modifying or symptom-modifying approaches (Schenkman et al. 2018). A large phase 3 study (SPARX3) has been registered to test whether high- versus moderate-intensity treadmill training modifies PD development over 12 months, implying maintained clinical interest in intensity as a signaling modulator (Moore et al. 2013; Schenkman et al. 2018; Patterson et al. 2022). Nevertheless, a lactate-centric PD model makes a disciplined prediction: HIE is most likely to be beneficial when lactate appears as repeated, well-cleared pulses associated with adaptive remodeling, and more likely to be harmful when PD-related mitochondrial constraint and neuroinflammation shift lactate into a persistent homeostatic collapse regime (Sects. "Oxidative stress, mitochondrial dysfunction, and neuroinflammation related to lactate homeostatic collapse" through "Potential links between lactate homeostatic collapse and α- Syn aggregation"). The practical implication is that HIE in PD should be framed as precision dosing—grounded in individual thresholds, recovery capacity, and disease stage—instead of a one-size-fits-all escalation of intensity.

Overall, high-intensity exercise builds the clearest biological setting for peak lactate exposure and hence the strongest opportunity to engage lactate-sensitive CNS pathways; yet it is also the regime where the distinction between adaptive signaling and metabolic overload becomes most consequential. The pathophysiological opportunity for PD is to exploit HIE’s capacity for tolerance remodeling—increasing clearance, buffering, and neurovascular support—while utilizing personalized monitoring to prevent crossing into acid–ion and inflammation-amplifying regimes (Goodwin et al. 2007).

### Lactate threshold as a benchmark for personalized exercise prescription

The appeal of lactate-threshold–based prescription is that it operationalizes exercise intensity utilizing an internal metabolic state instead of an external workload (speed, watts) or a population-normalized surrogate (for instance, %HR_\text{max} or %VO₂peak). Conceptually, lactate threshold (LT) marks the transition from a domain where lactate appearance and disappearance can be tightly matched to one where the system becomes progressively sensitive to small mismatches, yielding sustained lactate elevations and—at higher intensities—progressive accumulation. The original “anaerobic threshold” framework integrated gas-exchange breakpoints with blood lactate rise during incremental exercise, formalizing the idea that a biological transition can be detected non-invasively (Wasserman et al. 1973). Importantly, however, “lactate threshold” is not a single measurement, but a family of related concepts with different operational definitions and levels of validity, and these differences have direct consequences for both pathophysiological inference and clinical translation (Faude et al. 2009).

#### Why thresholds outperform “percent of maximum” for individualization

Using %HR_\text{peak} or %VO₂peak to prescribe intensity can produce large inter-individual variance in blood lactate and ventilatory responses at the “same” nominal intensity, which becomes especially problematic in clinical populations where autonomic control, medication, and movement economy are altered. A recent synthesis explicitly highlights that exercising at the same %HR_\text{peak}/%VO₂peak yields greater inter-individual variance in acute biological responses than exercising at (a fraction of) ventilatory threshold (VT) or lactate threshold (LT), motivating threshold-based prescription as a more physiologically anchored approach (Hansen et al. 2025).

For PD this point is not theoretical. Autonomic dysfunction and modified cardiovascular responses to exercise are common, and CPET-based analyses in PD link autonomic burden to decreased peak heart rate and modified cardiorespiratory responses (Veldkamp et al. 2025). Accordingly, HR-based targets can mis-dose the intended internal load—notably when the goal is to reproducibly engage lactate-linked mechanisms proposed in this review.

#### Which “threshold” should be used

There is no universal LT. LT1/VT1 marks the first sustained rise above baseline and is useful for sustainable training with limited lactate excursions (Faude et al. 2009). LT2/VT2, often near MLSS, marks the transition toward progressive accumulation and stronger lactate-signaling exposure, but also higher acid–ion risk (Beneke 2003). Fixed 4 mmol·L − 1 OBLA is pragmatic but not personalized and can misclassify steady-state boundaries (Heck et al. 1985). IAT was developed to address inter-individual threshold variability (Stegmann et al. 1981). Thus, LT validity depends on the intended endpoint: performance, MLSS, training-zone demarcation, or pathophysiological exposure (Faude et al. 2009).

#### What “gold standard” means: MLSS and why it matters

If the objective is to anchor intensity to the highest sustainable steady state (critical for long-duration prescriptions and for defining the heavy–severe boundary), then maximal lactate steady state (MLSS) remains a reference concept. MLSS is specific as the highest workload at which blood lactate remains effectively steady during prolonged constant-load exercise (often operationalized as ≤ 1 mmol·L − 1{−1} − 1 rise over a specific interval) (Beneke 2003). The methodological drawback—multiple constant-load sessions on separate days—limits clinical practicality, but its conceptual value is high: it separates “threshold” from any single incremental-test artifact and directly measures whether lactate dynamics stabilize (Beneke 2003).

In practice, numerous LT2/IAT methods aim to approximate MLSS more efficiently. The evidence base includes both supportive and critical findings: Stegmann and Kindermann demonstrated that work rates corresponding to an individual anaerobic threshold did not lead to gradual lactate accumulation or exhaustion during prolonged tests, in contrast to fixed 4 mmol prescriptions were less reliably steady-state across individuals—an empirical argument for personalized thresholds over fixed heuristics (Stegmann and Kindermann 1982). In contrast, McLellan and Jacobs explicitly challenged whether IAT, as defined, is always substantiated as the “highest steady state,” stressing that validity must be demonstrated, not assumed (Mclellan and Jacobs 1993).

#### Practical determination in PD: feasibility and methodological caution

Clinically, threshold prescription requires both a valid test and reliable threshold identification. Standard CPET does not consistently identify estimated lactate threshold or respiratory compensation point in every dataset, limiting routine implementation (Keltz et al. 2023). In PD, autonomic dysfunction and medication can weaken %HRmax-based zones (Veldkamp et al. 2025), while motor limitations and altered movement economy can decouple external workload from internal metabolic strain, increasing the value—but also the difficulty—of individualized testing (Veldkamp et al. 2025).

Collectively, these bottlenecks motivate a pragmatic hierarchy: when available, CPET with gas-exchange analysis (to derive VT1/VT2) with optional lactate sampling offers the most mechanistically interpretable anchors for intensity; when lactate sampling is not feasible, gas-exchange thresholds can serve as surrogates grounded in the same biological transition logic originally formalized by Wasserman and colleagues (Wasserman et al. 1973).

#### Why LT matters specifically for a lactate-mechanism PD review

A lactate-centric hypothesis builds an unusual demand on exercise prescription: it is not enough to say “moderate” or “vigorous”; the intervention must deliver a reproducible lactate exposure profile (amplitude × duration × recovery kinetics) that is comparable across individuals and across sessions. Threshold anchoring offersthe cleanest way to do this because it maps exercise intensity to the metabolic transition where lactate becomes a maintained systemic variable (Sect. " Moderate-intensity exercise: threshold crossing and adaptive lactate signaling") (Faude et al. 2009). This is especially relevant given that major PD trials have used HR-based intensities—e.g., SPARX (phase 2) and SPARX3 (phase 3) prescribe treadmill exercise at 80–85% vs 60–65% HR_\text{max} (Schenkman et al. 2018). HR-based prescriptions are pragmatic and scalable, but they cannot guarantee comparable lactate exposure across participants, especially in PD where autonomic function varies; LT anchoring could reduce this variance and refine pathophysiological interpretation.

#### A practical, testable prescription framework

**Fig. 3:**
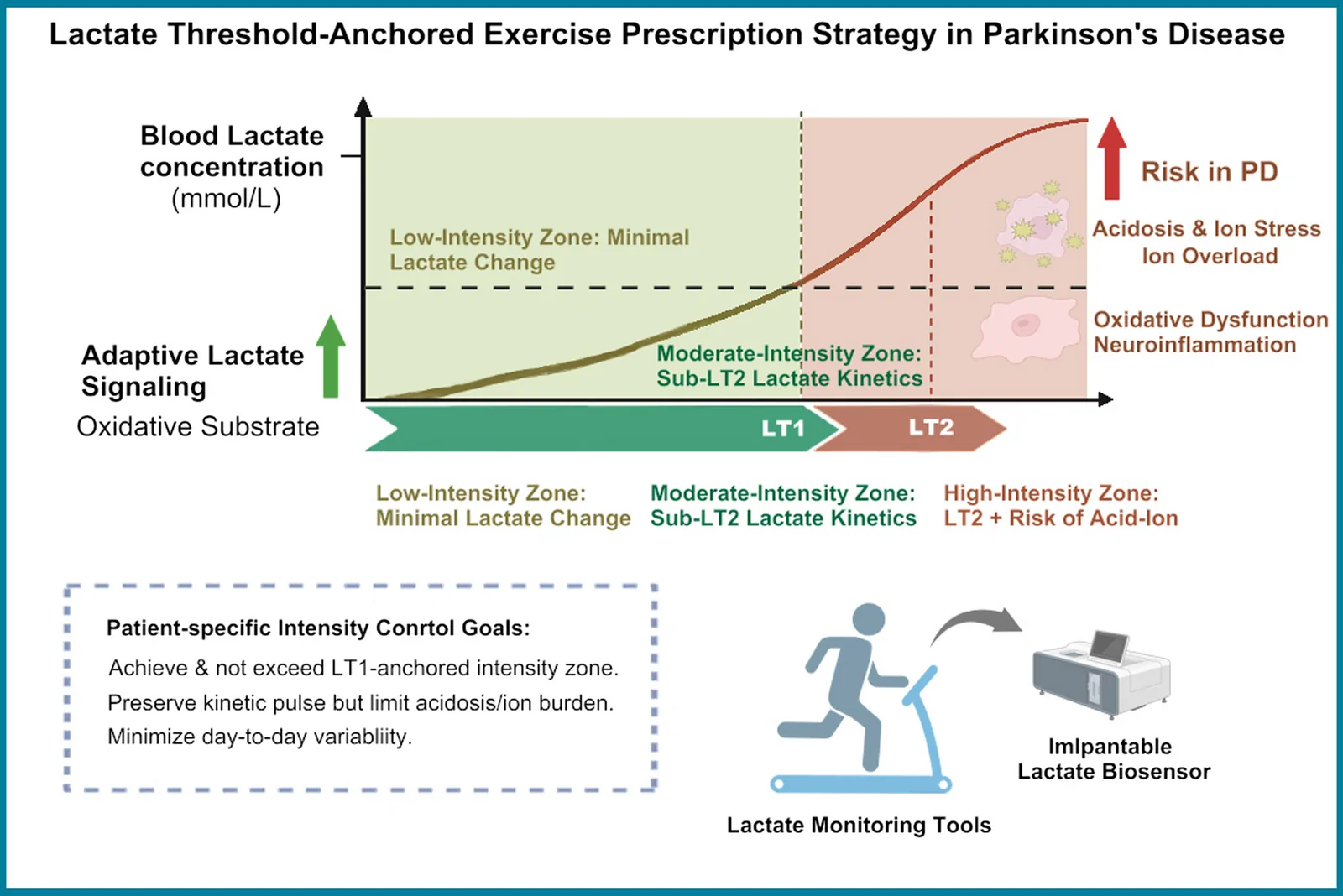
A lactate-threshold–anchored framework for personalized exercise dosing in PD. Legend: Exercise prescription in Parkinson’s disease (PD) may be improved by anchoring training intensity to individualized lactate-related internal load markers rather than relying exclusively on external workload or fixed percentages of maximal heart rate. In this framework, the first lactate or ventilatory threshold (LT1/VT1) marks the transition from a low-intensity steady-state domain, in which lactate concentration usually remains near baseline and the dominant adaptation is improved clearance and oxidative coordination, to a moderate domain in which lactate becomes a more reproducible systemic signal. The zone between LT1 and LT2 is proposed as a practical signaling window for many PD-oriented interventions, because it may allow sustained lactate elevation without progressive accumulation, thereby increasing the likelihood of adaptive substrate and signaling effects while limiting acid–ion liabilities. By contrast, workloads at or above the second lactate threshold (LT2), and especially near or above maximal lactate steady state (MLSS), are predicted to generate the largest lactate peaks but also the highest risk of drift toward maladaptive regimes, including acidification, ionic stress, oxidative strain and neuroinflammatory burden. The figure therefore illustrates a pragmatic three-domain strategy for PD: low intensity below LT1 to build tolerance and clearance capacity; moderate intensity between LT1 and LT2 to achieve reproducible lactate exposure with constrained accumulation; and sparing, carefully monitored excursions above LT2 when larger lactate pulses are specifically desired. Because PD is associated with autonomic variability, medication effects and altered movement economy, lactate-threshold anchoring is presented as a method for internal-load standardization rather than as a universal fixed-concentration rule. Monitoring tools shown here, including intermittent blood lactate assessment and emerging continuous biosensing platforms, are conceptual examples of how individualized lactate kinetics could be tracked to support precision exercise dosing

A practical PD framework has three domains: below LT1/VT1 for safe volume and adherence; LT1–LT2 for sustained lactate elevation without progressive accumulation, a candidate signaling window (Beneke 2003); and near/above LT2 or MLSS for occasional high-pulse exposure with careful recovery because acid–ion risk is greatest (Beneke 2003). Because thresholds depend on protocol, repeated testing may be needed (Faude et al. 2009), and 4 mmol·L⁻^1^ heuristics should remain rough, not personalized, anchors (Heck et al. 1985). A practical lactate-threshold–anchored framework for individualized exercise dosing in PD is summarized in Fig. 3.

## Summary: why LT is the right benchmark—if used with evidence-tier discipline

Lactate threshold is best framed as a benchmark for internal load standardization, not a single “magic point.” The foundational physiology linking lactate rise to systemic transition remains influential (Wasserman et al. 1973), but contemporary evidence and critique converge on a stricter message: threshold concepts are useful when they are clearly defined, methodologically consistent, and validated against the intended endpoint (steady-state sustainability, performance, pathophysiological exposure) (Faude et al. 2009). In PD, where autonomic and motor factors can distort conventional HR-based dosing, threshold-based prescription provides a plausible route to reduce inter-individual variance and to deliver reproducible lactate exposure profiles—an essential requirement if lactate is to be framed as a pathophysiological mediator instead of a correlational by-product of “exercise intensity” (Hansen et al. 2025). The current human, preclinical and translational evidence relevant to exercise-, lactate- and lactate-homeostasis-based interventions in PD is summarized in Table 3.

**Table 3:** Human and preclinical evidence relevant to exercise-, lactate- and lactate-homeostasis-based interventions in PD

Evidence tier	Study / model	Intervention or exposure	Lactate context	PD-relevant readout	Main finding	What it supports	Major limitation / inference boundary	References
Human clinical trial	**De novo PD, SPARX phase 2**	High-intensity treadmill exercise (versus comparator/usual care)	Lactate not measured directly, but high-intensity exercise is biologically the condition most likely to generate substantial systemic lactate excursions	Motor outcomes; feasibility; safety	High-intensity treadmill training was feasible and safe and met a predefined nonfutility signal for motor benefit	Supports the idea that **exercise intensity matters** in early PD and is compatible with a lactate-mediated contribution	Does **not** prove lactate mediation; exercise produces many parallel signals beyond lactate	Schenkman et al. 2018)
Human clinical imaging study	**Park-in-Shape neuroimaging subset**	Aerobic exercise (stationary cycling) versus stretching	Lactate not measured	Putamen-centred functional connectivity; cognitive control	Aerobic training enhanced anterior putamen–sensorimotor cortex connectivity and improved cognitive control relative to stretching	Supports a **striatal circuit-level benefit** from aerobic exercise, anatomically relevant to this review’s framework	Still indirect for lactate; no metabolic exposure profiling	Johansson et al. 2022)
Human dopaminergic imaging association	**Early/mild PD**	Six months of high-intensity exercise	Lactate not measured	DAT availability in substantia nigra / putamen	High-intensity exercise increased DAT availability in substantia nigra in most participants, with more variable putaminal change	Suggests exercise may strengthen **dopaminergic signaling capacity** in PD-relevant systems	Association only; not randomized proof of lactate mechanism	Laat et al. 2024)
Human enabling physiology	**Healthy human exercise / infusion physiology**	Lactate infusion plus cycling; exercise-induced hyperlactatemia	Arterial lactate rose from near-rest values to clearly elevated levels during infusion and exercise; brain switched toward lactate uptake and oxidation	Cerebral lactate uptake and oxidation	Human brain can import and oxidize circulating lactate when plasma lactate is elevated, including during exercise	Defines a physiological route for exercise-derived lactate to reach the brain	Not performed in PD; shows feasibility of exposure, not therapeutic effect	Hall et al. 2009; Boumezbeur et al. 2010)
Preclinical exercise–circuit evidence	**PD mouse model**	Aerobic training	Lactate not directly quantified	Striatal beta-band power; MSN firing; D2-MSNs overactivity; D2R expression	Exercise reduced beta power and MSN firing, with especially prominent normalization in D2-MSNs; D2R expression increased after training	Supports exercise-sensitive rescue of a D2-MSN-centered striatal bottleneck	Molecular mediator remains unresolved; lactate is plausible but unproven	Wang et al. 2024)
Preclinical dopaminergic exercise evidence	**MPTP mice**	Intensive treadmill training	Lactate not directly quantified	Striatal D2R expression; dopaminergic signaling markers	Intensive treadmill exercise increased striatal D2 receptor expression; related human work suggests improved residual dopaminergic function	Supports the broader idea that exercise can reshape **DA-linked striatal state**	Still cannot separate lactate from other training-induced adaptations	Vučković et al. 2010; Laat et al. 2024)
Preclinical lactate-homeostasis intervention	**PD model, SIRT1–PKM2 axis**	restoring lactate homeostasis through upstream glycolytic regulation	Directly lactate-relevant	Parkinsonian pathology / phenotype burden	Improving brain lactate homeostasis alleviated parkinsonism-like pathology, whereas excessive lactate accumulation appeared harmful	Strong support for the view that **lactate effects are state-dependent**, and that chronic homeostatic collapse may be pathogenic	Does not show that all lactate elevations are harmful; disease context and kinetics remain decisive	Lian et al. 2024)
Preclinical neuroinflammatory mechanism	**PD model, microglial lactylation**	Glycolysis-derived lactate / histone lactylation pathway	Directly lactate-relevant	Microglial activation; inflammatory reprogramming	Enhanced glycolysis-derived lactate promoted microglial activation through histone lactylation	Supports the maladaptive arm of your framework: lactate may act as a **state-setting inflammatory effector**	Primarily neuroimmune / model-based evidence; not yet circuit- or human-validated in detail	Qin et al. 2025)
Transport feasibility in PD model	**MPTP mouse**	Observation of MCT/GLUT transport-related proteins	Indirect lactate utilization context	BBB / striatum / SN transport capacity	One model reported preserved MCT1/MCT2 and GLUT1 expression, suggesting lactate transport capacity may remain available	Supports the feasibility that PD brain may still be able to **use incoming lactate**	Model- and timepoint-specific; preserved transporter expression is not the same as preserved flux	Puchades et al. 2013)
Translational prescription framework	**PD-oriented exercise dosing logic**	LT/VT-anchored intensity prescription; three-domain framework (below LT1, LT1–LT2, above LT2/near MLSS)	Lactate is treated as an internal-load variable requiring reproducible amplitude, duration and clearance kinetics	Standardization of exercise exposure; risk control	Threshold anchoring may standardize lactate exposure while limiting acid–ion risk	Supports the move from vague “moderate/high intensity” labels toward **lactate-aware precision rehabilitation**	Primarily a **testable framework**, not yet definitive clinical proof in PD	Wasserman et al. 1973; Faude et al. 2009; Beneke 2003; Stegmann and Kindermann 1982; Mclellan and Jacobs 1993; Keltz et al. 2023)

Bold type within the table body indicates keyclassification labels or descriptors used to organize the table and does not indicate statistical significance

## Future directions and translational prospects

### Advances in real-time lactate monitoring and modulation technologies

A lactate-centered framework for PD requires measurement tools that can capture kinetics, compartment, and context rather than isolated lactate values. This is a practical bottleneck: conventional blood draws, MRS readouts, and tissue assays are often episodic or spatially coarse, whereas lactate biology is dynamic and state-dependent. Recent progress in wearable sensing, microneedle sampling, genetically encoded reporters, and metabolic imaging is beginning to make lactate kinetics experimentally and translationally tractable.

#### Wearable and minimally invasive lactate monitoring: from trend tracking to clinically meaningful kinetics

Sweat- and textile-based platforms offer a field-compatible route for monitoring lactate trends during exercise. For example, electrochemical textile systems can combine lactate oxidase-based sensing with pH co-monitoring and wireless data capture (Napier et al. 2024). The pH channel is important because lactate changes cannot be interpreted cleanly without considering acid–base state. However, sweat lactate is affected by sweat rate, skin contamination, gland physiology, and diffusion delay, so it should be used mainly for within-person kinetics rather than as a direct blood surrogate.

Interstitial fluid (ISF) microneedle sensing has a clearer near-term path for PD exercise dosing because ISF is more closely coupled to blood than sweat and can be monitored continuously with minimal invasiveness. Human-validated microneedle lactate patches can track real-time lactate dynamics and show agreement with venous lactate up to approximately 13 mmol/L (Ming et al. 2022). Integrated intradermal ISF platforms further support progress toward stable, wearable lactate monitoring (Wang et al. 2024). These tools could help individualize exercise intensity in PD, especially when heart-rate targets are confounded by autonomic dysfunction or medication effects.

Continuous intravascular lactate monitoring is less relevant to routine PD care but useful conceptually. Critical-care systems based on intravascular microdialysis show that lactate clearance trajectories can be operationalized as clinically meaningful kinetic variables rather than single time-point measurements (Daurat et al. 2020). This supports a broader principle for PD trials: lactate rise, peak, and recovery may be more informative than resting lactate alone.

#### High-resolution lactate sensing in neural tissue: resolving compartment and causality

Peripheral traces are insufficient for testing whether lactate acts directly in PD-relevant basal ganglia circuits. The key mechanistic need is to resolve whether lactate changes occur in extracellular space, astrocytes, neurons, mitochondria, or specific striatal microdomains, and whether these changes precede or follow circuit dysfunction.

Genetically encoded sensors are central to this goal. Laconic and Pyronic established FRET-based strategies for measuring lactate and pyruvate flux, including the use of MCT perturbation to infer directionality of metabolite movement (San Martín et al. 2013, 2014). Such tools helped define experimental logic for testing whether lactate is exported, imported, oxidized, or redistributed. Subsequent sensor-based work shows that astrocytic lactate dynamics can be rapidly recruited by neuronal activity-linked signals, including extracellular K⁺ (Sotelo-Hitschfeld et al. 2015). Newer eLACCO/iLACCO and related single-wavelength or FLIM-compatible sensors expand the toolkit for extracellular, intracellular, and potentially in vivo lactate imaging (Nasu et al. 2021, 2023). Tools such as LiLac and CanlonicSF further expand the experimental choice set, with trade-offs in affinity, pH sensitivity, dynamic range, photostability, and compatibility with multiplexed in vivo imaging (Koveal et al. 2022; Aburto et al. 2022). For PD, the decisive application is not simply detecting lactate elevation, but mapping which cell type and compartment generate or consume lactate during dopamine loss, exercise, or inflammatory activation.

Label-free chemical imaging provides another useful route. CMOS-based multichemical sensors can simultaneously visualize lactate and H⁺ dynamics, directly addressing the lactate–pH confound that complicates interpretation of toxicity versus signaling (Doi et al. 2025). Hyperpolarized ^13C metabolic MRI offers a complementary flux-based approach by monitoring pyruvate-to-lactate conversion and related metabolic products in vivo (Jørgensen et al. 2022). Human-brain applications are improving, including spatial and kinetic modeling for cerebral metabolism (Blazey et al. 2025). For PD, the value would be to map whether glycolytic bias emerges in basal ganglia regions such as the putamen and whether interventions normalize these flux signatures. The limitation is practical: hyperpolarized imaging still requires specialized tracers, hardware, and workflows, and current spatial resolution may restrict fine-grained basal ganglia interpretation (Page et al. 2020).

#### From monitoring to modulation: toward lactate-aware closed-loop control

The broader biosensing field is moving from passive measurement toward closed-loop systems that combine sensing, adaptive algorithms, and responsive intervention (Paci et al. 2025). For PD, the most realistic near-term application is lactate-aware exercise dosing. Continuous ISF or sweat lactate monitoring could allow training intensity to be adjusted in real time to maintain a personalized lactate zone: below LT1 for clearance-focused work, between LT1 and LT2 for sustained metabolic signaling, or controlled excursions above LT2 for pulse-like stimulation. This remains to be standardized clinically, but existing wearable and microneedle platforms are mature enough to support controlled PD rehabilitation trials (Ming et al. 2022).

More speculative applications include lactate-informed pharmacological, buffering, anti-inflammatory, or neuromodulatory loops. These should not be built around lactate alone. Because lactate can reflect adaptive substrate flux, receptor signaling, redox change, or acid–ion stress, closed-loop rules would need multi-parameter inputs such as lactate, pH, activity context, and possibly glucose, oxygen, or inflammatory markers. Simultaneous lactate–pH monitoring is therefore especially important for distinguishing adaptive pulses from chronic homeostatic collapse (Doi et al. 2025).

#### Critical challenges and the similarly “next inflection point”

Three constraints are most relevant to PD. First, technology choice should match the question: sweat devices are best suited for adherence and trend tracking, ISF microneedles for personalized dosing, MRS or hyperpolarized imaging for regional metabolic flux, and genetically encoded sensors for mechanism. Second, lactate should be paired with pH and, where possible, glucose or oxygen proxies, because lactate-only signals cannot distinguish adaptive flux from acid–redox stress (Napier et al. 2024). Third, lactate kinetics must be linked to PD-relevant endpoints, including D2-MSN excitability, ERK signaling, beta synchrony, MCT/HCAR1 perturbation, gait metrics, and motor scores.

Overall, real-time lactate monitoring is becoming a practical tool for testing—not assuming—the role of lactate in PD. Its strongest translational use is likely precision exercise dosing and pharmacodynamic readout, whereas its strongest mechanistic use is compartment-resolved validation of whether lactate acts as support, signal, or dysregulation in basal ganglia circuits. These tools are summarized in Table 4.

**Table 4:** Translational tools for measuring and modulating lactate dynamics in PD

Tool / approach	What it measures or controls	Spatial / temporal profile	Main strength	Main limitation	Most relevant PD use-case	Maturity for PD translation	References
Intermittent blood lactate testing	Peripheral lactate concentration during or after exercise	Systemic; intermittent; minute-scale sampling	Low cost, clinically accessible, already familiar in exercise physiology	Poor CNS specificity; single time-point values are often weakly informative in isolation	Exercise dosing, threshold determination, internal-load standardization	**High** for exercise use; **limited** as stand-alone PD biomarker	Goodwin et al. 2007; Wasserman et al. 1973; Faude et al. 2009)
Lactate / ventilatory threshold testing (LT1, LT2, VT1)	Individualized internal-load breakpoints that shape lactate appearance, accumulation and clearance	Whole-body integrative physiology; test-session based	Standardizes lactate exposure more precisely than vague intensity labels	Indirect for brain lactate; requires testing infrastructure and repeat calibration	Personalized exercise prescription in PD	**High** as a practical framework; still needs PD-specific validation as a lactate-guided strategy	Wasserman et al. 1973; Beneke 2003; Stegmann and Kindermann 1982; Mclellan and Jacobs 1993; Keltz et al. 2023)
Continuous sweat lactate sensors / wearable patches	Peripheral lactate trends during ongoing activity	Scalable, non-invasive, near-real-time	High convenience and adherence potential; suitable for repeated field monitoring	Sweat lactate is indirect and affected by local physiology; mechanistic interpretability is limited	Home-based dosing, adherence tracking, broad rehabilitation monitoring	**Moderate**; promising for deployment but limited for causal inference	Napier et al. 2024; Ming et al. 2022)
Interstitial-fluid (ISF) lactate sensing / microneedle platforms	Continuous lactate kinetics in interstitial fluid	More proximal to tissue milieu than sweat; near-real-time	Better physiological interpretability than sweat; compatible with continuous monitoring logic	More invasive than wearables; calibration, stability and user tolerance remain challenges	Personalized calibration, real-time exercise control, pharmacodynamic tracking	**Moderate** and rising	Napier et al. 2024; Ming et al. 2022)
Intracranial / implantable biosensors	Local extracellular lactate dynamics in defined brain regions	Highest local specificity; real-time	mechanistically decisive for linking lactate kinetics to circuit activity	Highly invasive; not broadly clinically deployable	Experimental closure in PD-relevant models; rare specialized human settings	**Low clinically**, **high mechanistically **	Ming et al. 2022; Doi et al. 2025)
Genetically encoded lactate sensors	Cell- and compartment-resolved lactate dynamics	High spatial specificity; real-time in experimental systems	Enables direct mapping of lactate flux in neurons, astrocytes and microdomains	Preclinical tool; not a routine human translational platform	Establishing necessity/sufficiency and cell-type specificity in PD models	**High for mechanism**, **low for near-term clinical translation **	Ming et al. 2022; Doi et al. 2025)
CSF lactate measurement	Bulk CNS-adjacent lactate level	Coarse CNS integrator; intermittent	Closer to CNS metabolism than blood; can be interpreted alongside tau/Aβ/DA-related measures	Invasive; small effect sizes; not region-specific or PD-specific	Stratification feature, pathophysiological correlate	**Low to moderate **	Liguori et al. 2022)
Blood metabolomics panels including lactate-related features	Broader glycolysis / energy-metabolism signature	Systemic; sampling-based	Better than lactate alone for stratification; scalable across cohorts	Brain specificity remains limited; composite models may not isolate lactate’s role	Peripheral stratification, challenge-based biomarker panels	**Moderate **	(Mallet et al. 2022; Lope et al. 2024)
Hyperpolarized [1-13C]pyruvate MRS/I	Dynamic conversion to [1-13C]lactate and bicarbonate, reflecting glycolytic vs oxidative flux balance	Regional imaging; dynamic but infrastructure-intensive	Strong conceptual bridge from “lactate concentration” to “metabolic flux phenotype”	Specialized hardware/workflows; limited scalability and fine-grained BG resolution	Regional bioenergetic phenotyping, intervention response mapping	**Emerging / moderate **	Jørgensen et al. 2022; Blazey et al. 2025; Page et al. 2020)
31P-MRS and related bioenergetic imaging	ATP, Pi, phosphocreatine and energetic state rather than lactate directly	Regional imaging; non-invasive	Useful for defining the mitochondrial/energetic backdrop that gates whether lactate is adaptive or pathological	Does not directly quantify lactate flux	Contextualizing lactate findings within PD bioenergetic phenotype	**Moderate **	Payne et al. 2024)
Closed-loop lactate-aware exercise control	Uses continuous lactate kinetics to adjust exercise intensity in real time	Real-time feedback control	Most immediate and ethically straightforward path from monitoring to intervention	Needs robust sensor stability, algorithms and validated decision rules	Precision rehabilitation and zone-targeted exercise dosing	**Emerging but testable now **	Napier et al. 2024; Paci et al. 2025)
Multi-analyte closed-loop systems (lactate + pH + activity context ± glucose/oxygen proxies)	Integrates lactate with contextual variables to avoid overinterpreting lactate alone	Real-time, multimodal	Better suited to lactate’s dual beneficial/pathological role than lactate-only systems	Greater technical complexity and validation burden	Distinguishing adaptive lactate pulses from chronic homeostatic collapse	**Conceptually strong, early-stage **	Napier et al. 2024; Doi et al. 2025; Paci et al. 2025)
Targeted modulation of MCTs / HCAR1	Controls lactate transport or extracellular lactate signaling	Mechanism-targeted rather than measurement-focused	Directly aligned with the two main therapeutic target classes discussed in the manuscript	Cell-type and compartment specificity remain major unresolved issues	Mechanistic perturbation and future therapeutic development	**Preclinical / exploratory **	Morland et al. 2017; Buscemi et al. 2021)

Bold type within the table body indicates keyclassification labels or descriptors used to organize the table and does not indicate statistical significance

### Therapeutic approaches targeting lactate transport (MCTs) and signaling (HCAR1)

Therapeutic interest in lactate has shifted from treating it as a passive metabolic by-product to engineering a flux and signaling system whose outputs can be beneficial (substrate support, inhibitory stabilization, neurovascular adaptation) or harmful (acid–ion stress, inflammatory state-locking, pathological plasticity). Two target classes dominate the current translational landscape: proton-linked monocarboxylate transporters (MCTs; SLC16 family) that recognize where lactate (and accompanying H⁺) moves, and the lactate GPCR HCAR1 (GPR81) that converts extracellular lactate into Gi/o-coupled signaling. A central principle—often underemphasized in “drug target” discussions—is that manipulating lactate transport is rarely a lactate-only intervention: because MCT1–4 co-transport lactate with a proton, changing lactate flux will also rewire pH microdomains, redox coupling and ion homeostasis, which are especially consequential in PD-relevant striatal tissue (Sect. " Lactate-related disruption of striatal function: detrimental mechanisms") (Pierre and Pellerin 2005).

#### Targeting MCTs: from “blocking lactate” to reshaping compartmental flux

CNS isoforms display strong cellular partitioning that immediately constrains therapeutic plausibility: MCT1 is prominent at barriers and astrocytes (and also present in other CNS cell types), MCT4 is often described as astrocyte-enriched and adapted for lactate export, and MCT2 is enriched in neurons and associated with lactate uptake/oxidation capacity (Pierre and Pellerin 2005). This distribution is not just descriptive; it predicts that the same inhibitor can shift flux in opposite directions depending on which cellular interface is rate-limiting (astrocyte → neuron, neuron → extracellular space, blood → brain). A second, druggability-relevant constraint is that MCT membrane localization and function depend on accessory proteins (notably basigin/CD147 and embigin), which act as chaperones and gate transporter availability at the surface. Cryo-EM structures of MCT1 in complex with basigin-2, captured with lactate and prototypical inhibitors (including AZD3965), and recent structural work on the MCT2–embigin complex (including inhibitor-bound states) provide a progressively precise blueprint for isoform-selective and complex-selective modulation (Wang et al. 2021).

Positive strategy may be to inhibit lactate transport to inhibit pathological lactate/H⁺ efflux. The most mature pharmacology for MCTs comes from oncology, where **blocking lactate export** is intended to trap lactate and H⁺, collapse pH homeostasis and constrain tumor metabolism. Syrosingopine was reported to inhibit both MCT1 and MCT4, avoiding lactate and H⁺ efflux and generating synthetic lethality with mitochondrial complex I inhibition (metformin) in highly glycolytic contexts (Benjamin et al. 2018). For PD, the conceptual attraction of this approach is that it could counteract a chronic high-lactate inflammatory state (for instance, in glycolysis-shifted microglia). The dominant limitation is safety logic: in striatum and other neural tissue, trapping lactate + H⁺ is not only “lowering lactate signaling”—it risks local acidification and ion-pump compromise, specifically the injury cascade discussed in Sect. " Acidification and disruption of ionic homeostasis". Hence, a transport-blockade strategy is difficult to justify without tight compartment targeting (e.g., microglia-restricted delivery) and real-time pH/lactate monitoring to prevent converting “metabolic normalization” into acidotoxic stress.

It is also important to facilitate lactate transport and utilization to support circuit performance. A mechanistically opposite strategy—arguably tighter to the exercise-linked beneficial regime—would be to increase lactate availability to neurons when lactate is adaptive (transient pulses; adequate oxidative reserve). Here the principal risk is that neuronal lactate import is not optional in all contexts: disrupting neuronal MCT2 (and astrocyte-derived lactate supply) can weaken long-term memory and plasticity, supporting the idea that lactate import can become functionally required in demanding regimes (Suzuki et al. 2011). In motor systems pivotal to PD, astrocytic lactate export capacity also appears plasticity-relevant: astrocyte-specific MCT4 knockdown in main motor cortex weakened motor learning and decreased dendritic spine density, with decreased activity markers across motor regions including dorsal striatum (Lundquist et al. 2022). While this is not a striatal D2-MSN-specific experiment, it provides a rare causal link between MCT-induced lactate handling and motor-circuit plasticity, strengthening the rationale for exploring MCT4/MCT2 modulation in rehabilitation settings instead of viewing MCTs exclusively as “metabolic housekeeping”. The translational bottleneck is drug modality: “increasing” an SLC transporter is harder than inhibiting it. The most realistic near-term routes are (i) state-dependent delivery of exogenous lactate (or lactate-generating interventions, such as exercise) instead of transporter agonism, and (ii) gene- or cell-programming approaches that adjust transporter expression or trafficking in specific compartments—approaches that become credible only if lactate microdomain kinetics can be monitored (Sect. " Advances in real-time lactate monitoring and modulation technologies").

Selective inhibition as a pathophysiological probe—then precision translation. The clinical development path of MCT inhibitors illustrates both opportunity and warning. AZD3965 is a first-in-class MCT1 inhibitor advanced to human trials (in oncology), and the dose-escalation results confirm tolerability at doses achieving target engagement, while also revealing on-target liabilities including ocular findings and (at higher doses) metabolic acidosis—risks that are directly pivotal to any CNS program built on lactate trapping (Halford et al. 2023). In human oncology experience, MCT1 inhibition has also been associated with severe hyperlactatemia and malignant hyperlactatemic acidosis following AZD3965 exposure, underscoring that lactate/proton transport blockade carries direct systemic metabolic risks rather than only theoretical CNS concerns (Mcneillis et al. 2020). Even in preclinical settings, systemic MCT1 inhibition has been associated with measurable CNS effects (for instance, memory retention defects observed as partial and transient in mice in one preclinical safety characterization), reinforcing that MCT modulation is not metabolically neutral for brain function (Benyahia et al. 2021).

MCT inhibitors are currently best positioned as pathophysiological tools to test necessity/sufficiency of lactate flux for specific striatal phenotypes (D2-MSN excitability, beta synchrony, plasticity rules), instead of as ready-to-deploy therapeutics. Translation would need (i) isoform/cell-type selectivity, (ii) BBB-appropriate pharmacokinetics, and (iii) explicit safety gating on pH/ion homeostasis.

#### Targeting HCAR1: engineering a lactate-to-Gi signaling axis

HCAR1 provides a receptor-defined route for testing lactate signaling, but its therapeutic relevance in PD remains unproven. Unlike strategies that alter lactate production, transport, or clearance, HCAR1 targeting attempts to modulate how extracellular lactate is interpreted by cells. Neuronal work in primary cortical cultures showed that HCAR1 activation with 3-chloro-5-hydroxybenzoic acid decreases excitatory synaptic transmission and firing, and that these effects are lost in HCAR1 knockout preparations, supporting a receptor-mediated inhibitory action of lactate in non-basal-ganglia systems (de Castro Abrantes et al. 2019). This inhibitory logic is relevant to PD only as a candidate mechanism. It could, in principle, dampen excessive activity during transient lactate elevations, but chronic receptor engagement could also compress dynamic range, worsen hypokinesia, or blunt adaptive plasticity. HCAR1 should therefore be treated as a state-dependent lactate-sensing route, not as a constitutive protection receptor.

A recent structural advance strengthens the pharmacological tractability of this receptor without establishing PD efficacy. Cryo-EM structures of human HCAR1 in complex with Gi proteins, including agonist-bound and apo forms, provide a molecular basis for ligand recognition and receptor activation, supporting the rational design of selective agonists and potentially biased ligands (Pan et al. 2025). For CNS translation, however, several additional barriers remain: candidate ligands would need adequate BBB penetration, limited peripheral metabolic effects such as adipose lipolysis suppression, and a signaling profile that avoids excessive neuronal inhibition. At present, these structural data justify pharmacological exploration of HCAR1, but they do not demonstrate that receptor modulation will improve PD motor function.

One of the strongest causal examples of lactate–HCAR1 signaling in brain adaptation comes from exercise-linked neurovascular remodeling. Exercise or systemic lactate enhanced VEGFA expression and capillary density in mouse brain in wild-type, but not Hcar1 knockout animals, supporting HCAR1 as an initiating signal for lactate-linked neurovascular adaptation (Morland et al. 2017). In PD, this mechanism is attractive because improved neurovascular or metabolic support could increase circuit resilience during rehabilitation. However, it remains indirect: HCAR1-dependent angiogenesis would be expected to increase metabolic headroom rather than directly restore dopamine signaling or normalize basal ganglia output. A cautious translational strategy would therefore treat HCAR1 modulation, if pursued, as an adjunct to rehabilitation or metabolic-state monitoring, not as a stand-alone symptomatic therapy.

Importantly, HCAR1 agonism does not necessarily reproduce all effects of lactate. In a mouse ischemia–reperfusion paradigm, HCAR1 agonists, including 3,5-dihydroxybenzoic acid and CHBA, did not reproduce the protective effects observed with lactate administration (Buscemi et al. 2021). This negative result is important for PD translation because it argues against a simple equivalence between lactate benefit and HCAR1 activation. Lactate can act through substrate provision, redox-sensitive signaling, receptor activation, and longer-timescale inflammatory or lactylation-linked mechanisms; these routes must be separated experimentally before HCAR1 can be interpreted as a therapeutic target.

The opposite strategy, HCAR1 antagonism, would be rational only if PD were shown to involve persistent extracellular lactate elevations that chronically engage HCAR1 and impair striatal circuit function. This remains untested. A rigorous case for antagonism would require evidence that striatal lactate reaches receptor-relevant concentrations in vivo, that HCAR1 is functionally engaged in defined basal ganglia cell types, and that blocking this receptor beneficially alters D2-MSN activity, beta synchronization, motor output, or plasticity without removing an adaptive feedback signal. Until such data are available, HCAR1 agonists and antagonists should be viewed primarily as experimental tools for dissecting lactate signaling, rather than as ready therapeutic candidates for PD.

#### Convergent design principles for PD: precision, compartment, and kinetics

Three design principles follow. First, compartment-selective intervention is safer than systemic modulation because MCT blockade carries acidosis and CNS liabilities (Halford et al. 2023). Second, kinetics determine directionality: pulse-like lactate/HCAR1 signaling may be adaptive, whereas chronic lactate dysregulation may promote inflammatory and ion-homeostatic stress. Third, mechanisms must be validated by striatal cell type and disease stage because D2-MSNs, astrocytes, microglia, endothelial cells and DA terminals may respond differently to identical lactate levels (Xu et al. 2026).

### Personalized metabolic–neuromodulation treatment frameworks

Personalized metabolic–neuromodulation strategies should be framed as conservative state-management frameworks rather than as additive combinations of exercise, pharmacology, and device therapy. The central idea is to align intervention timing with measurable circuit and metabolic states: neurophysiological markers can index pathological synchronization, digital biomarkers can quantify motor fluctuations, and lactate kinetics can define metabolic exposure, clearance capacity, and recovery profile (Hu et al. 2024; Sun et al. 2024; de Neeling et al. 2026; Busch et al. 2025).

Three interaction rules are most relevant. First, neuromodulation can change metabolism by altering movement, autonomic tone, and cortico–BG dynamics. Second, metabolic state can change neuromodulation efficacy because lactate-related variables, pH, redox balance, and inflammation may alter synaptic gain and excitability thresholds. Third, kinetics determine directionality: pulse-like lactate exposure during rehabilitation may differ fundamentally from maintained lactate elevation associated with homeostatic collapse. These rules argue against treating lactate as a direct control signal for DBS at this stage. A safer near-term use is to use lactate kinetics as a contextual marker for scheduling and interpreting rehabilitation, medication state, and neuromodulation response.

The near-term research agenda should therefore remain incremental: calibrate lactate kinetics as an exercise-dosing variable in PD; test whether lactate-anchored intensity prescription reduces inter-individual variability compared with heart-rate-based dosing; integrate lactate kinetics with digital motor outcomes and neurophysiological markers; and determine whether metabolic-state stratification improves response to rehabilitation or neuromodulation. The standard should be falsifiable kinetic hypotheses linked to patient-relevant outcomes, including motor vigor, OFF time, dyskinesia burden, falls, fatigue, and objective gait or bradykinesia measures.

Overall, personalized metabolic–neuromodulation frameworks are best framed not as a single existing therapy, but as a control strategy: continuously infer the patient’s circuit-and-metabolic state, then apply neuromodulation, pharmacology and exercise as synchronized actuators to maintain stability while preserving the capacity for adaptive plasticity. The scientific standard for this field should be rigorous: pathophysiological endpoints, validated biomarkers, and explicit kinetic hypotheses that can be falsified—because PD is heterogeneous enough that “integrated” approaches will otherwise succeed only rhetorically.

### Translational positioning of lactate as a biomarker and response-monitoring variable in PD

Lactate is attractive in PD precisely because it lies at the intersection of glycolytic flux, cytosolic redox balance, mitochondrial reserve, inflammatory programming, and exercise responsiveness. That same integrative position, however, makes it difficult to deploy as a clinically useful biomarker without explicit contextualization. A translationally useful framing should therefore not ask whether lactate is “a biomarker of PD” in the abstract; it should ask which biological question lactate is intended to answer, in which compartment, under which perturbation, and for which clinical decision. Within this framework, lactate is currently best positioned not as a stand-alone diagnostic discriminator, but as (i) a pharmacodynamic readout for therapies targeting lactate handling or glycolysis-linked inflammation, (ii) a challenge–response variable for exercise-based interventions, and (iii) a component of multimodal metabolic stratification when interpreted alongside imaging, metabolomics, and clinical phenotyping.

#### What biological question is lactate meant to answer?

Three distinct biomarker functions should be separated: **(1) Prognostic**, which predicts future clinical development independent of treatment; **(2) Predictive**, which identifies the likelihood of response to a specific intervention; and **(3) Pharmacodynamic/response**, which quantifies whether a therapy engages its intended metabolic target. Today, lactate is most credible as a pharmacodynamic/state biomarker and as a node of multi-parameter prognostic models, instead of as a stand-alone diagnostic discriminator.

#### Peripheral lactate and blood-based metabolomics: high scalability, low specificity

Single-time-point blood lactate is rarely informative for PD in isolation. Nevertheless, metabolomics studies consistently implicate glycolysis-linked and comprehensive energy-metabolism signatures in PD cohorts. A large translational NMR metabolomics study spanning multiple animal models and de novo PD cohorts demonstrated glycolysis-linked metabolite perturbations (including pyruvate and lactate changes in preclinical models) and derived a composite serum biomarker (a multi-metabolite panel instead of lactate alone) that discriminated de novo PD from controls (Mallet et al. 2022). This work also explicitly notes the limitation that blood only partially mirrors brain-region metabolism and that specificity versus atypical parkinsonian syndromes is not guaranteed—an important constraint for clinical deployment (Mallet et al. 2022).

Recent blood metabolomics studies further indicate that PD is accompanied by coordinated metabolomic alterations, supporting the feasibility of peripheral metabolic stratification while cautioning that lactate may not be the dominant discriminant metabolite across all datasets (Lope et al. 2024). The practical implication is that lactate may be best used peripherally as a dynamic challenge marker (see below) or as one feature within comprehensive metabolic models, instead of as a static diagnostic test.

#### CSF lactate: tighter to CNS metabolism, but still a coarse integrator

CSF lactate is conceptually tighter to brain metabolism and less directly contaminated by muscle lactate dynamics than blood, but it remains a bulk measure that cannot localize striatal microdomains. The most informative human evidence comes from a CSF study in drug-naïve PD (101 patients; 60 controls) that demonstrated modestly higher CSF lactate in PD and stratified patients by Hoehn & Yahr stage, alongside measurements of Aβ42, tau proteins and (in a subset) DA and DOPAC (Liguori et al. 2022). This design is valuable because it tests lactate in the same biospecimen as established neurodegeneration markers and interprets lactate as a marker of cerebral metabolic integrity instead of as an isolated metabolite (Liguori et al. 2022).

Nevertheless, CSF lactate faces two critical limitations for “progression” claims. First, the expected effect sizes are small in comparison with biological variance introduced by sampling conditions and comorbidities. Second, CSF lactate is not PD-specific; it can rise in multiple conditions involving mitochondrial dysfunction or inflammation. Therefore, even supportive studies should be interpreted as conceptualizing CSF lactate as a candidate stratification feature or a pathophysiological correlate, not a stand-alone disease-development biomarker.

#### Brain energetic imaging: shifting from “lactate concentration” to “bioenergetic phenotype”

Because lactate biology is inseparable from mitochondrial capacity in PD hypotheses, a pragmatic biomarker strategy is to combine lactate-related measures with direct bioenergetic imaging.

A representative example is a multimodal study utilizing 31P-MRS to quantify ATP, Pi and phosphocreatine in putamen and midbrain in recent-onset PD, alongside deep phenotyping of mitochondrial/lysosomal role in patient-derived fibroblasts. The authors report enhanced variance in posterior putaminal Pi/ATP in PD (interpreted as weakened oxidative phosphorylation) and correlations between putaminal Pi/ATP and peripheral indices consistent with mitophagy/mitochondrial uncoupling; they also identify a relationship between midbrain phosphocreatine and predicted risk of swift disease progression (Payne et al. 2024). Although this is not a lactate measurement, it is directly pivotal to lactate as a biomarker because it defines a metabolic insufficiency axis that would be expected to gate whether lactate elevations are adaptive (oxidizable, transient) or pathological (persistent, associated with redox stress).

By contrast, 1H-MRS studies in PD have historically produced mixed results in BG and cortex, often focusing on NAA/Cr and NAA/Cho instead of reliably quantifying lactate peaks because lactate detection is technically demanding and sensitive to acquisition parameters and overlap. An illustrative STN-DBS follow-up study used 1H-MRS to track metabolite ratios in globus pallidus and fronto-basal cortex and demonstrated cortical NAA/Cho and NAA/Cr increases correlating with UPDRS improvement, demonstrating that MRS can capture therapy-linked metabolic shifts even when lactate itself is not the main readout (Llumiguano et al. 2008). The biomarker lesson is that lactate may be more tractable as part of an integrated metabolic imaging panel than as a lone MRS peak.

#### Lactate–lactylation axis as a biomarker of inflammatory state and therapeutic engagement

An especially promising, PD-relevant “lactate biomarker” perspective is not lactate concentration, but lactate-dependent molecular writing, especially in microglia. One study reports that inhibiting glycolysis with 2-deoxy-D-glucose decreased lactate accumulation, microgliosis and dopaminergic damage in PD mouse models, and identifies enhanced microglial histone lactylation (notably H3K9la) with pathophysiological linkage to transcriptional regulation (including SLC7A11) and functional rescue via pathway targeting (Qin et al. 2025). Meanwhile, a study proposes that “excessive lactate in the brain” contributes to PD development and that restoring lactate homeostasis through a SIRT1–PKM2 mechanism alleviates parkinsonism in mice (Lian et al. 2024). These results do not establish lactylation as a ready clinical biomarker, but they do support a concrete translational hypothesis: lactate homeostatic collapse may be most harmful when it preserves inflammatory transcriptional states, suggesting that lactate-linked epigenetic marks (in CSF cells, extracellular vesicles, or peripheral immune cells) could serve as pharmacodynamic biomarkers for therapies aimed at normalizing immunometabolism. The main evidence gap is human validation with cell-type specificity and longitudinal association with progression.

#### Treatment response: why lactate kinetics may outperform lactate levels

For treatment response, lactate should be treated as a challenge–response variable. In exercise, amplitude, time-to-peak, and clearance slope may index metabolic capacity; in SIRT1–PKM2-type interventions, lactate kinetics and lactate/pyruvate ratios are more mechanistically relevant than single concentrations (Lian et al. 2024). DBS can alter brain metabolic signatures, but whether lactate kinetics predict DBS response remains testable (Llumiguano et al. 2008). A methodological corollary is that using lactate as a response biomarker will require protocol standardization—fasting state, medication state, time of day, workload calibration, and, crucially, clear definition of the intended intensity domain—to ensure interpretability across trials.

#### A realistic lactate-informed biomarker qualification pathway

A lactate-informed PD biomarker should not be designed as a stand-alone diagnostic test. The near-term goal should be response monitoring and stratification. A practical panel should combine standardized lactate kinetics, lactate/pyruvate ratio with acid–base markers, inflammatory or immunometabolic markers, circuit/bioenergetic readouts, and clinical endpoints such as MDS-UPDRS III, gait variability, bradykinesia, and exercise tolerance.

Validation should proceed in stages. First, establish analytical reliability of lactate kinetics in PD under standardized exercise or infusion paradigms. Second, test biological validity by linking lactate kinetics to bioenergetic or neurophysiological endpoints. Third, test clinical relevance by asking whether baseline kinetics or clearance slopes predict training response, medication response, fatigue, dyskinesia risk or disease progression. Fourth, test specificity against atypical parkinsonism, metabolic comorbidities, diabetes, mitochondrial disorders and inflammatory disease. This qualification path matches the evidence base: lactate is unlikely to classify PD, but it may identify metabolic state, treatment engagement and individualized exercise dose.

## Conclusion

Lactate should no longer be framed as a passive by-product of glycolysis in PD. Across the evidence reviewed here, lactate emerges as a dynamic control variable at the interface of substrate supply, redox and pH homeostasis, neuromodulatory signaling, and transcriptional state. In the striatum—where computation is bioenergetically limited and DA shapes plasticity rules—this conceptualization is mechanistically consequential. A unifying conclusion is that the consequences of lactate are context dependent. Transient, well-buffered lactate elevations can underpin metabolic flexibility, engage plasticity-linked kinase programs (including ERK/MAPK–CREB), and recruit inhibitory/neurovascular pathways (notably HCAR1), thereby buffering circuits and extending metabolic headroom. In contrast, maintained lactate homeostatic collapse—often associated with acidification, weakened ion transport, mitochondrial stress and neuroinflammation—can amplify vulnerability via ionic collapse risk, oxidative injury, and lactylation-linked state-locking. PD similarly shifts the boundary between these regimes by impairing dopaminergic gain control, decreasing mitochondrial reserve and priming inflammation, decreasing the threshold at which lactate-related pH/redox perturbations become destabilizing. The cellular locus with the clearest translational leverage is the D2-MSNs/indirect-pathway axis, which provides a parsimonious substrate for hypokinesia and abnormal network dynamics. Lactate is well positioned to intersect this bottleneck as fuel, receptor ligand and redox modulator, and through partial convergence with D2R-linked inhibitory signaling logic. Yet a decisive limitation remains: cell-type- and compartment-resolved causal evidence in striatal D2-MSNs is still sparse, leaving a gap between plausibility and mechanism.

The translational outlook is nonetheless increasingly actionable because enabling technologies are converging. Continuous and compartment-resolved lactate sensing (wearables/ISF patches, genetically encoded reporters, flux-sensitive imaging) can convert static correlations into falsifiable kinetic hypotheses. Meanwhile, structural and pharmacological advances for MCTs and HCAR1 make it feasible to perturb lactate flux and signaling with increasing specificity—although safety bottlenecks are intrinsic because transport manipulation co-moves protons and can destabilize pH/ion homeostasis. These considerations argue for precision dosing instead of generic lactate elevation. Personalized exercise anchored to lactate thresholds provides a pragmatic route to deliver reproducible lactate kinetics while minimizing acid–ion and inflammatory liabilities. More broadly, the most credible future perspective is not a single “lactate drug”, but integrated metabolic–neuromodulation approaches that coordinate neurotransmitter control (dopaminergic therapy, A2A antagonism, adaptive DBS) with metabolic state management (exercise dosing, anti-inflammatory glycolytic reprogramming control, lactate flux/signaling modulation). Finally, lactate is unlikely to function as a stand-alone disease-development biomarker, but it has realistic potential as a state and pharmacodynamic biomarker when framed as kinetics (appearance/clearance and lactate–pyruvate/redox context) and interpreted alongside bioenergetic and inflammatory readouts. In translational terms, the key challenge is not to maximize lactate exposure, but to define the exposure profiles that are mechanistically informative, biologically tolerable, and clinically actionable in PD.

## Data Availability

No datasets were generated or analysed during the current study.

## Abbreviations

ANLS: Astrocyte–neuron lactate shuttle
ATP: Adenosine triphosphate
BBB: Blood–brain barrier
BG: Basal ganglia
CNS: Central nervous system
CREB: CAMP response element-binding protein
CSF: Cerebrospinal fluid
DA: Dopamine
DAT: Dopamine transporter
D2-MSNs: D2 medium spiny neurons
ERK: Extracellular signal-regulated kinase
FDG-PET: Fluorodeoxyglucose positron emission tomography
GPCR: G protein-coupled receptor
HCAR1: Hydroxycarboxylic acid receptor 1
iSPN: Indirect-pathway spiny projection neuron
LDH: Lactate dehydrogenase
LID: L-DOPA-induced dyskinesia
MCT: Monocarboxylate transporter
MRS: Magnetic resonance spectroscopy
MSN: Medium spiny neuron
NMDAR: N-methyl-D-aspartate receptor
OXPHOS: Oxidative phosphorylation
PD: Parkinson’s disease
ROS: Reactive oxygen species
SNc: Substantia nigra pars compacta
STN: Subthalamic nucleus
